# Supplementary figures and images for: Genetic basis of thermal plasticity variation in Drosophila melanogaster body size
Source: PLoS Genet. 2018 Sep 26;14(9):e1007686. doi: 10.1371/journal.pgen.1007686 (PMC6175520; doi:10.1371/journal.pgen.1007686)

**A**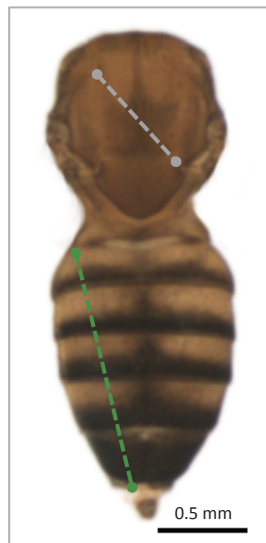**B**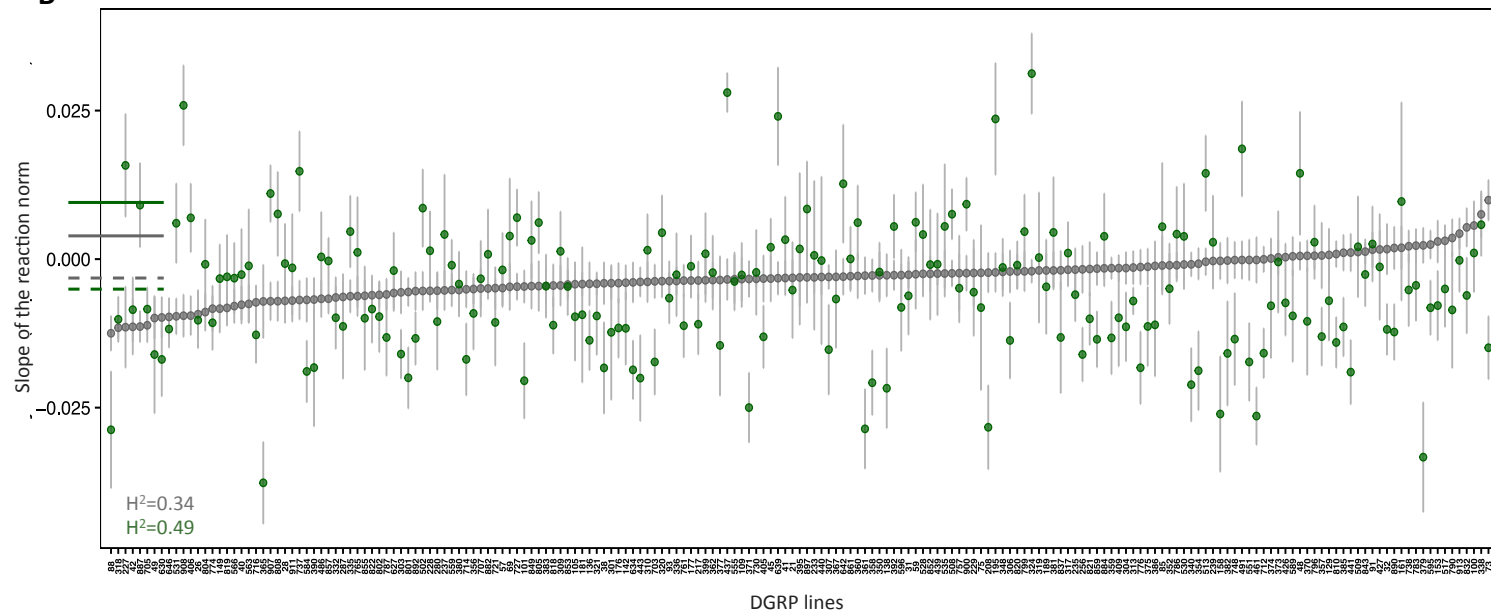**C**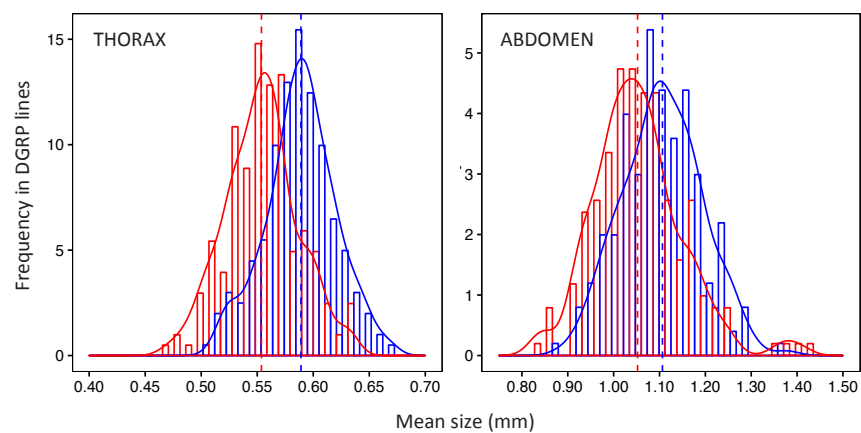**D**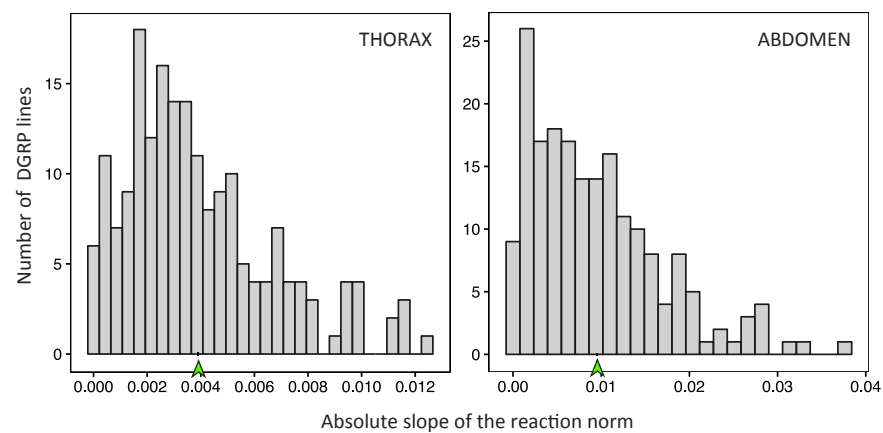

Supplement: S1 Fig — A. Image of an adult female D. melanogaster fly showing the thoracic and abdominal transects. B. Slope and confidence interval of the reaction norms in the DGRP lines, calculated as the regression model lm (Size ~ Temperature) in the thoraxes (grey) and abdomens (green) of each DGRP line (Y axis). Slopes are ranked by their value in the thorax. Horizontal bars represent the mean of all DGRP lines for the raw slope of the reaction norm (dashed bar) and the absolute slope of the reaction norm (solid bar) per body part. C. Histograms showing the frequency of the size measurements in thoraxes and abdomens of all DGRP lines reared at 17°C (blue) and 28°C (red). Dashed line represents the mean value for all DGRP lines at a given temperature. D. Histograms for the absolute slope of the reaction norms (calculated as the absolute value for the slope of the regression lm (Size ~ Temperature) in thoraxes and abdomens. Mean value for the absolute slope of all DGRP lines is indicated with a green arrowhead. (PDF) [file pgen.1007686.s001.pdf]

**A**

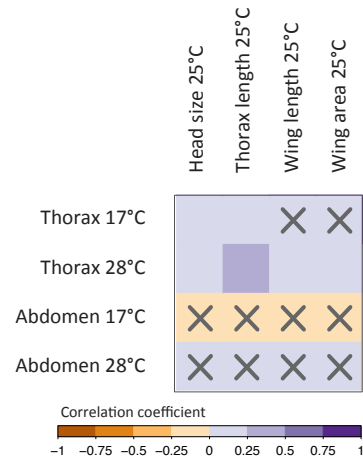

**B**

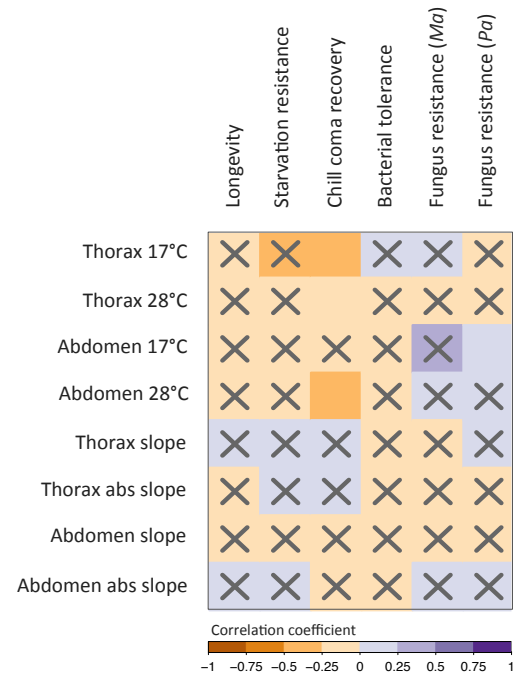

**C**

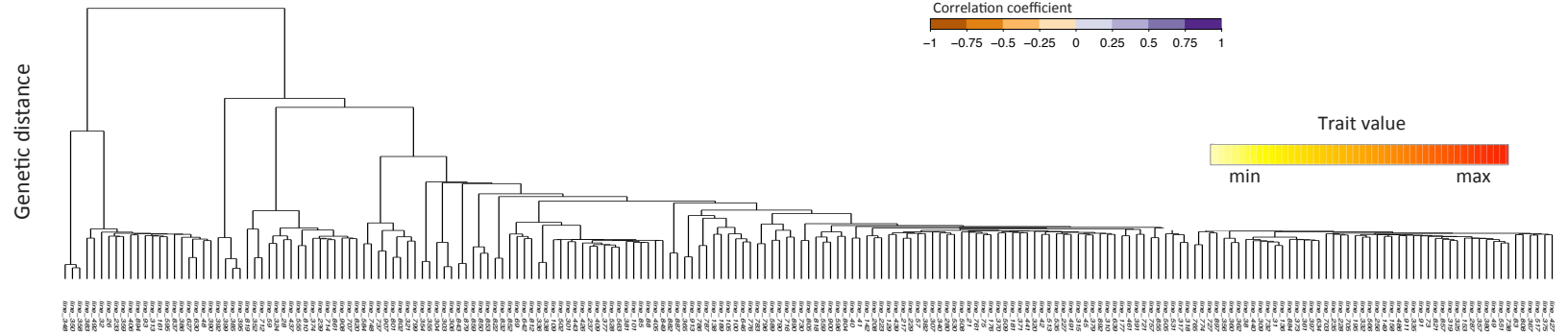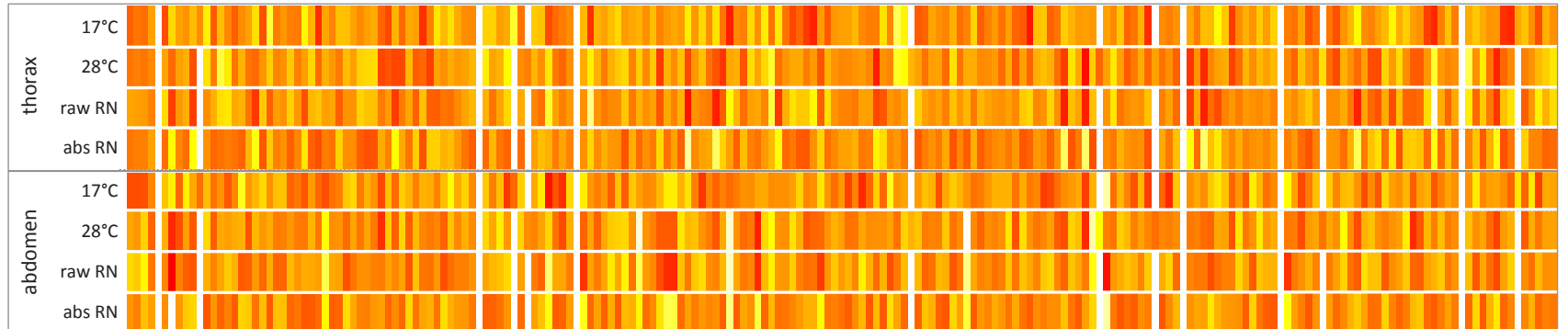

Supplement: S2 Fig — A. Heat map of Pearson’s correlation coefficients between our within-environment size measurements (17°C and at 28°C) and size measurements at 25°C. Non-significant correlations (p-value > 0.01) are indicated with an ‘X’. B. Heat map of Pearson’s correlation coefficients between our traits (mean size at each temperature and raw and absolute slopes of the reaction norms) and fitness-related traits. Non-significant correlations (p-value > 0.01) are indicated with an ‘X’. C. Dendogram of the genetic distance between DGRP lines. Corresponding trait values are shown as a heat map and scaled for each trait independently. Coefficients of Blomberg’s K phylogenetic signal were: K = 0.24; p-value = 0.17 (thorax at 17°C), K = 0.23; p-value = 0.37 (thorax at 28°C), K = 0.22; p-value = 0.78 (thorax raw slope), K = 0.24; p-value = 0.20 (thorax absolute slope), K = 0.24; p-value = 0.24 (abdomen at 17°C), K = 0.22; p-value = 0.67 (abdomen at 28°C), K = 0.23; p-value = 0.55 (abdomen raw slope) and K = 0.21; p-value = 0.89 (abdomen absolute slope). Pagel’s λ coefficient of phylogenetic signal was λ = 6.88e-05; p-value = 1, for all the traits in both body parts. (PDF) [file pgen.1007686.s002.pdf]

A

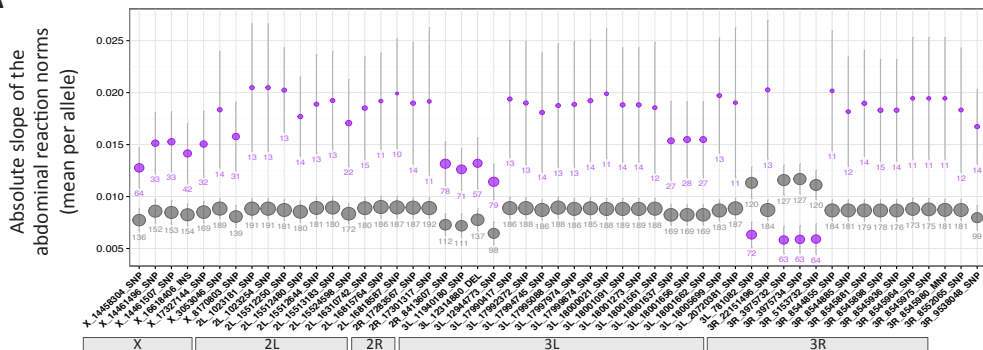

B

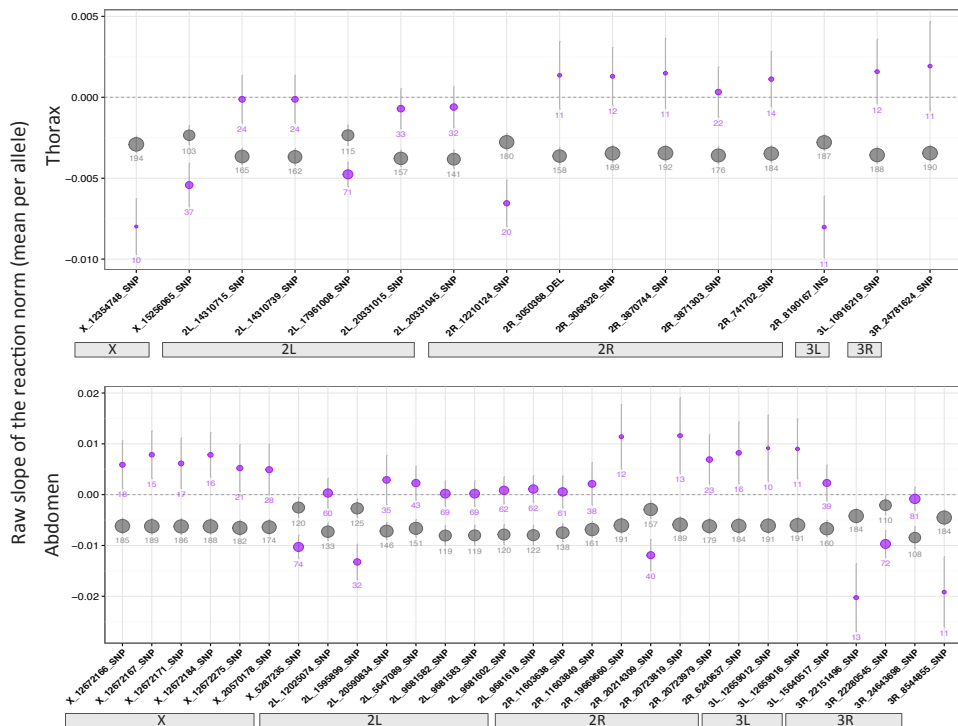

Significant (p-value &lt; 10e-5) SNP/InDel per chromosomal arm

Supplement: S5 Fig — A. Mean and confidence interval of the absolute slope of the reaction norms for abdomen size (Y axis) per allele (minor allele in grey, minor allele in magenta) at each candidate plasticity SNP/Indel (p-value < 10e-5) along the chromosomal arms (X axis). B. Mean and confidence interval of the raw slope of the reaction norms for size (Y axis) per allele (minor in grey, minor in magenta) at each candidate plasticity QTL (p-value < 10e-5) along the chromosomal arms (X axis) per body part. The position and identity of the polymorphisms in this figure is given by their annotation with Genome Release v.5. (PDF) [file pgen.1007686.s005.pdf]

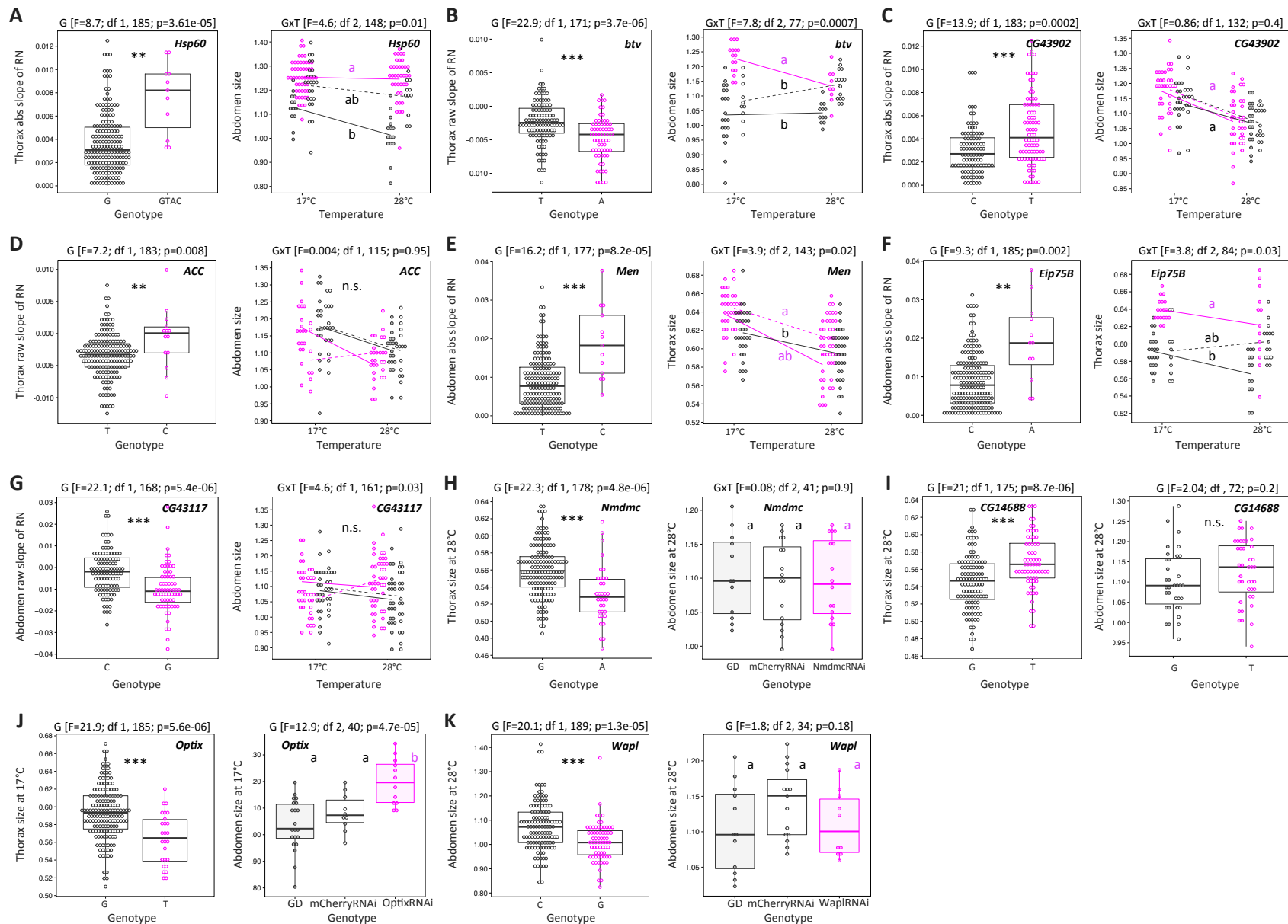

Supplement: S7 Fig — For each candidate QTLs, the effect of the minor and major alleles in the DGRPs are shown in the left panels and the pleitropic effect is shown in the right panels. For validations via mutant or RNAi, genotypes with impaired gene function are shown in magenta and control genotypes are shown in black. Similarly, for validations via Mendelian Randomization (MR), the two populations fixed for the minor allele are shown in magenta and the two populations for the major allele are shown in black. The identity of the SNP/Indels is given by their annotation with Genome Release v.6. as Chromosome:Position. Size measurements are given as length in mm. A. Left panel: slope of the reaction norms in the DGRP lines with the major and the minor alleles for insertion in position X:11108613, within gene Hsp60. Right panel: reaction norms in mutant Hsp60A/+ and controls Canton-S (filled circles, solid line) and Fm7a/Canton-S (empty circles, dashed line). B. Left panel: slope of the reaction norms in the DGRP lines with the major and the minor alleles for SNP in position 2L:17961008, within gene btv. Right panel: reaction norms for size in btv-RNAi/bab-Gal4 and control lines KK (filled circles, solid line) and mCherry-RNAi/bab-Gal4 (empty circles, dashed line). C. Left panel: slope of the reaction norms in the DGRP lines with the major and the minor alleles for SNP in position X:10192303, within gene CG43902. Right panel: reaction norms in the four MR populations corresponding to SNP in position X:10192303, within gene CG43902. D. Left panel: slope of the reaction norms in the DGRP lines with the major and the minor alleles for SNP in position 2R:7983239, within gene ACC. Right panel: reaction norms in the four MR populations for SNP in position 2R:7983239 within gene ACC. E. Left panel: slope of the reaction norms in the DGRP lines with the major and the minor alleles for SNP in position 3R:12720159 within gene Men. Right panel: reaction norms in Men mutants MenEx3/+ (filled circles, [file pgen.1007686.s007.pdf]
