# Supplementary material for: Genetic basis of thermal plasticity variation in Drosophila melanogaster body size
Source: PLoS Genet. 2018 Sep 26;14(9):e1007686. doi: 10.1371/journal.pgen.1007686 (PMC6175520; doi:10.1371/journal.pgen.1007686)
Supplement: S3 Fig — Manhattan plots corresponding to the four GWAS performed for variation in size plasticity: raw slopes of the reaction norms (grey dots) and absolute slope of the reaction norms (black dots) for thorax (left side) and abdomen (right side) size. For each trait and body part, the GWAS was done testing the model lm (Slope ~ Allele + (1|Wolb/DGRP)). The significance level for each SNP along the chromosomal arms is shown as the log10 p-value. Some of the genes associated to SNPs/Indels with a p-value < 10e-5 and that we consider as particularly interesting are shown. The position and identity of the polymorphisms in this figure is given by their annotation with Genome Release v.5. (PDF) [file pgen.1007686.s003.pdf]

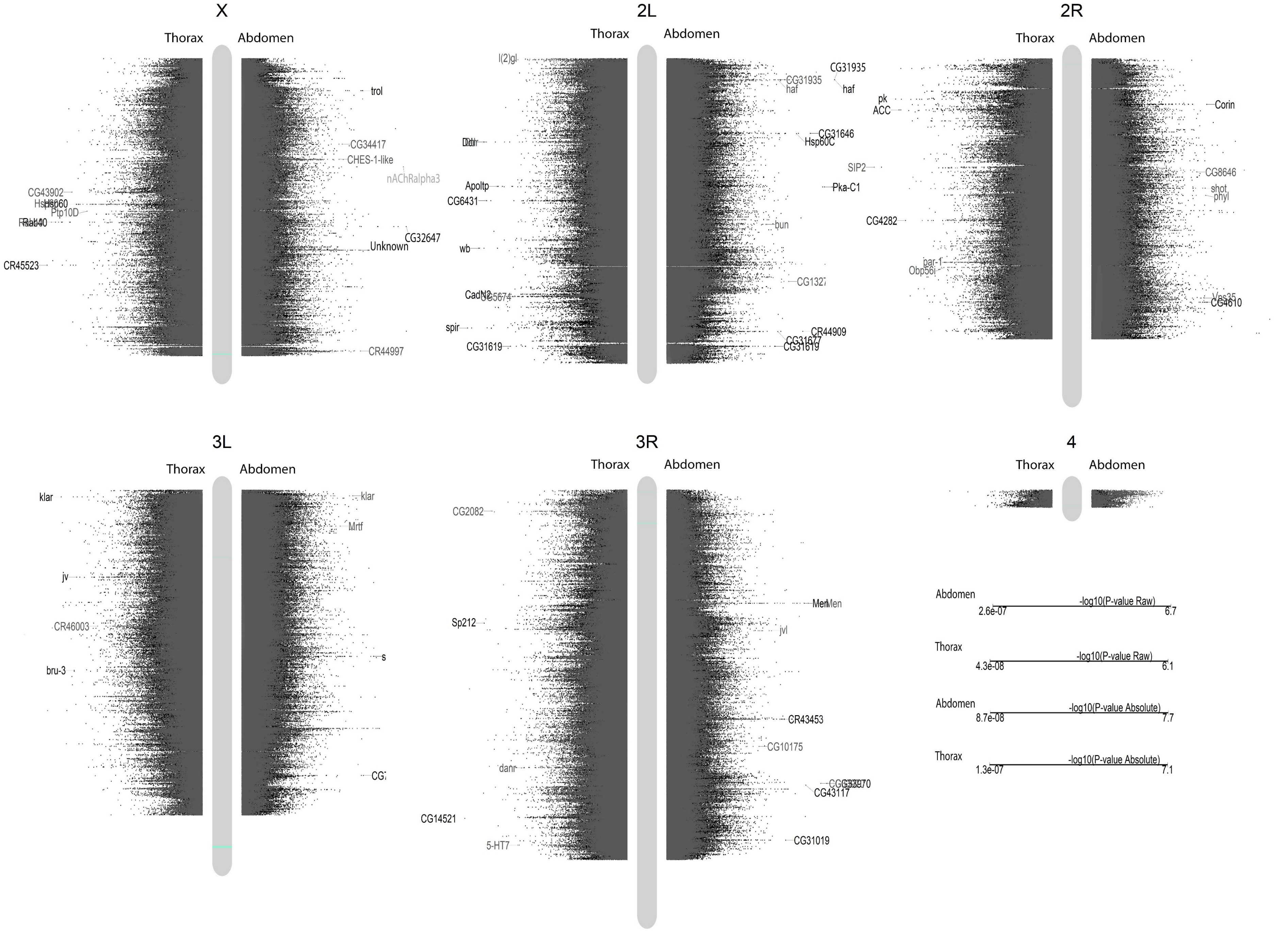
X
Thorax
Abdomen
trol
CG34417
CHES-1-like
nAChRalpha3
CG43902
Hsp60
Ptp10D
Rab40
CG32647
Unknown
CR45523
CR44997
2L
l(2)gl
CG31935
haf
Ddr
Hsp60C
CG31646
Apoltp
Pka-C1
CG6431
bun
wb
CG1327
CadN2
CG5674
spir
CR44909
CG31677
CG31619
2R
pk
ACC
Corin
SIP2
CG8646
shot
phyl
CG4282
par-1
Obp56i
Vps35
CG4610
3L
klar
Mrtf
jv
CR46003
s
bru-3
3R
CG2082
Men
Sp212
jvl
CR43453
CG10175
danr
CG43117
CG14521
5-HT7
CG31019
4
Abdomen
-log10(P-value Raw)
2.6e-07
6.7
Thorax
-log10(P-value Raw)
4.3e-08
6.1
Abdomen
-log10(P-value Absolute)
8.7e-08
7.7
Thorax
-log10(P-value Absolute)
1.3e-07
7.1
