# Supplementary material for: Genetic basis of thermal plasticity variation in Drosophila melanogaster body size
Source: PLoS Genet. 2018 Sep 26;14(9):e1007686. doi: 10.1371/journal.pgen.1007686 (PMC6175520; doi:10.1371/journal.pgen.1007686)
Supplement: S4 Fig — Manhattan plots corresponding to the four GWAS performed for within-environment variation in size: at 17°C (blue dots) and at 28°C (red dots) for thorax (left side) and abdomen (right side) size. For each trait and body part, the GWAS was done testing the model lm (Size ~ Allele + (1|Wolb/DGRP)). The significance level for each SNP along the chromosomal arms is shown as the log10 p-value. Some of the genes associated to SNPs/Indels with a p-value < 10e-5 and that we consider as particularly interesting are shown. The position and identity of the polymorphisms in this figure is given by their annotation with Genome Release v.5. (PDF) [file pgen.1007686.s004.pdf]

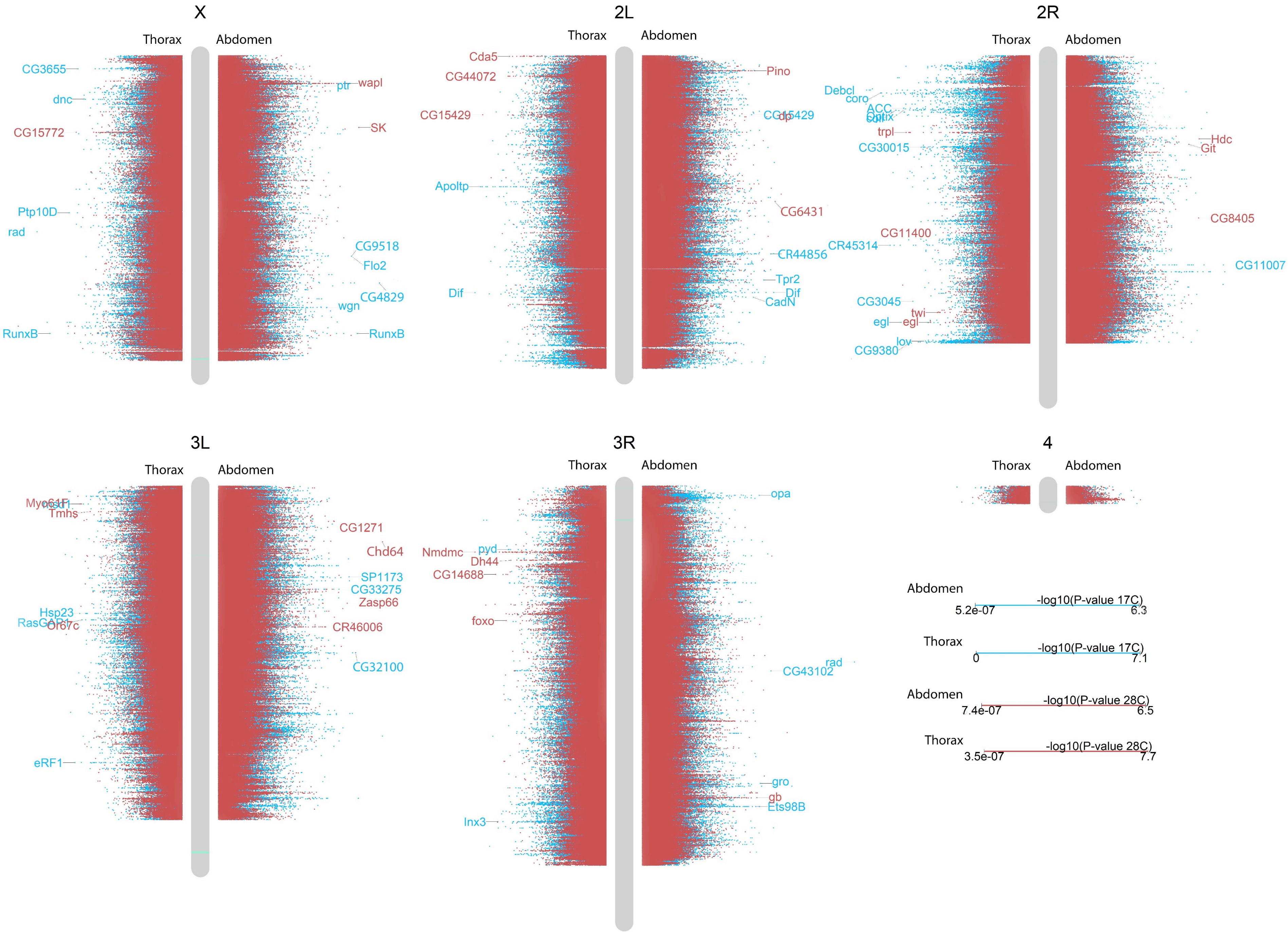

X
Thorax
Abdomen
CG3655
dnc
CG15772
Ptp10D
rad
RunxB
ptr
wapl
SK
CG9518
Flo2
CG4829
wgn
RunxB
2L
Thorax
Abdomen
Cda5
CG44072
CG15429
Apoltp
Dif
Pino
CG15429
dp
CG6431
CR44856
Tpr2
Dif
CadN
2R
Thorax
Abdomen
Debcl
coro
ACC
trpl
CG30015
CG11400
CR45314
CG3045
twi
egl
egl
lov
CG9380
Hdc
Git
CG8405
CG11007
3L
Thorax
Abdomen
Tmhs
Hsp23
Or67c
eRF1
CG1271
Chd64
SP1173
CG33275
Zasp66
CR46006
CG32100
3R
Thorax
Abdomen
Nmdmc
pyd
Dh44
CG14688
foxo
Inx3
opa
rad
CG43102
gro
gb
Ets98B
4
Thorax
Abdomen
Abdomen
-log10(P-value 17C)
5.2e-07
6.3
Thorax
-log10(P-value 17C)
0
7.1
Abdomen
-log10(P-value 28C)
7.4e-07
6.5
Thorax
-log10(P-value 28C)
3.5e-07
7.7
