# Supplementary material for: Genetic basis of thermal plasticity variation in Drosophila melanogaster body size
Source: PLoS Genet. 2018 Sep 26;14(9):e1007686. doi: 10.1371/journal.pgen.1007686 (PMC6175520; doi:10.1371/journal.pgen.1007686)
Supplement: S6 Fig — A-B. Gene ontology and network enrichment analyses for genes harboring allelic variants significantly associated with variation in size plasticity. All SNPs/Indels with a p-value < 10e-5 from the GWAS for the raw and absolute slopes of the reaction norms of thoraxes and abdomen were pooled to perform these analyses. A. Results of the gene-ontology enrichment analysis for genes harboring allelic variants significantly associated with variation in size reaction norms. B. Network enrichment analyses (KEGG gene enrichment analyses of the protein-protein interactions) for genes harboring allelic variants significantly associated with variation in size reaction norms. All our candidate QTLs for variation in size plasticity were pooled for this analysis. This corresponds to the QTLs from four independent GWAS: raw and absolute values of the slopes of the reaction norms for thoracic and abdominal size. C. Network enrichment analyses (KEGG gene enrichment analyses of the protein-protein interactions) for genes harboring allelic variants significantly associated with variation in size reaction norms. SNPs with p-value < 10e-5 from the GWAS for variation at 17°C and for variation at 28°C were pooled to perform this analysis. Note that for allelic variants associated with variation in size plasticity, the gene-ontology enrichment analyses showed no enriched GO terms. All our candidate QTLs for variation in size were pooled for this analysis. This corresponds to the QTLs from four independent GWAS: thoracic and abdominal size for flies reared at 17°C and at 28°C. (PDF) [file pgen.1007686.s006.pdf]

A

GO term	Description	p-value	FDR
GO:0006897	Endocytosis	3,25E-05	5,12E-02
GO:0006909	Phagocytosis	3,25E-05	2,56E-02
GO:0098657	Import into cell	3,25E-05	1,71E-02
GO:0016192	Vesicle-mediated transport	1,82E-04	7,19E-02
GO:0051049	Regulation of transport	4,44E-04	1,40E-01

B

Pathway (KEGG)	p-value	FDR
SNARE interactions in vesicular transport	2,33E-15	2,95E-13
Notch signaling pathway	1,96E-05	0,00125
Protein processing in endoplasmic reticulum	0,000141	0,00599
Wnt signaling pathway	0,000221	0,007
Hedgehog signaling pathway	0,000461	0,0117
Endocytosis	0,000562	0,0119
Ubiquitin mediated proteolysis	0,00309	0,056
Mismatch repair	0,0103	0,163
Progesterone-mediated oocyte maturation	0,0151	0,213
TGF-beta signaling pathway	0,0247	0,314
Phagosome	0,0297	0,325
Homologous recombination	0,0307	0,325
Ribosome	0,0426	0,416

C

Pathway (KEGG)	p-value	FDR
Protein processing in endoplasmic reticulum	2,71E-08	3,44E-06
Ribosome	1,12E-06	7,14E-05
Endocytosis	0,00324	0,137
RNA transport	0,0198	0,569
Wnt signaling pathway	0,0224	0,569
Propanoate metabolism	0,0352	0,672
TGF-beta signaling pathway	0,037	0,672
Citrate cycle (TCA cycle)	0,0758	1
Notch signaling pathway	0,0883	1
Protein export	0,0883	1
