# Supplementary material for: Genetic basis of thermal plasticity variation in Drosophila melanogaster body size
Source: PLoS Genet. 2018 Sep 26;14(9):e1007686. doi: 10.1371/journal.pgen.1007686 (PMC6175520; doi:10.1371/journal.pgen.1007686)
Supplement: S1 Table — (DOCX) [file pgen.1007686.s008.docx]

| **S1 Table. Variance components.**  Genetic variance = variance associated to genetic differences. Residual variance = variance associated to residual differences. GxT variance = variance associated to genotype-by-temperature interaction component. See materials and methods for details on the performed calculations. | | | | | |
| --- | --- | --- | --- | --- | --- |
| **Body part** | **Trait** | **Genetic variance** | **Residual variance ^6^** | **GxT variance** | **Total variance** |
|  |  |  |  |  |  |
| Thorax | 17°C | 7.98e-04 | 1.6e-03 | NA | NA |
|  | 28°C | 8.7e-04 | 1.6e-03 |  |  |
| Abdomen | 17°C | 6.6e-03 | 7.6e-03 |  |  |
|  | 28°C | 8.7e-03 | 7.9e-03 |  |  |
| Thorax | slope | 2.0e-04 | 1.6e-03 | 6.0e-04 | 2.3e-03 |
| Abdomen | slope | 1.0e-03 | 7.8e-03 | 6.2e-03 | 1.5e-02 |
