# Supplementary material for: Genetic basis of thermal plasticity variation in Drosophila melanogaster body size
Source: PLoS Genet. 2018 Sep 26;14(9):e1007686. doi: 10.1371/journal.pgen.1007686 (PMC6175520; doi:10.1371/journal.pgen.1007686)
Supplement: S2 Table — (DOCX) [file pgen.1007686.s009.docx]

| **S2 Table. Summary of phenotypic measurements for size and size plasticity in the DGRP.**  N = Number of phenotyped flies, SD= standard deviation, CV= coefficient of variation. Mendelian Randomization (MR) populations fixed for either the major or the minor allele of each candidate SNP; MR1 for SNP within gene *CG43902*, MR2 for SNP within gene *ACC*, MR3 for SNP within gene *CG43117*, and MR4 for SNP within gene *CG14688*. | | | | | | | | | | | | |
| --- | --- | --- | --- | --- | --- | --- | --- | --- | --- | --- | --- | --- |
| **DGRP line** | **Body part** | **N 17** | **Mean 17** | **SD 17** | **CV 17** | **N 28** | **Mean 28** | **SD 28** | **CV 28** | **Raw slope** | **Absolute slope** | **MR population** |
| line_100 | Abdomen | 9 | 1.0911 | 0.0775 | 7.1057 | 9 | 1.2500 | 0.0612 | 4.8990 | 0.0144 | 0.0144 | NA |
| line_100 | Thorax | 9 | 0.5678 | 0.0323 | 5.6920 | 9 | 0.6300 | 0.0548 | 8.6940 | 0.0057 | 0.0057 | NA |
| line_101 | Thorax | 14 | 0.5992 | 0.0318 | 5.2997 | 22 | 0.5484 | 0.0439 | 8.0005 | -0.0046 | 0.0046 | NA |
| line_101 | Abdomen | 14 | 1.2543 | 0.1264 | 10.0736 | 22 | 0.9400 | 0.0879 | 9.3472 | -0.0286 | 0.0286 | NA |
| line_105 | Abdomen | 11 | 1.1400 | 0.0756 | 6.6343 | 20 | 1.1680 | 0.0977 | 8.3684 | 0.0025 | 0.0025 | NA |
| line_105 | Thorax | 11 | 0.6410 | 0.0443 | 6.9165 | 22 | 0.5945 | 0.0548 | 9.2208 | -0.0042 | 0.0042 | NA |
| line_109 | Abdomen | 10 | 1.0700 | 0.1055 | 9.8612 | 6 | 0.9483 | 0.0256 | 2.7022 | -0.0111 | 0.0111 | NA |
| line_109 | Thorax | 10 | 0.6070 | 0.0343 | 5.6565 | 6 | 0.5700 | 0.0400 | 7.0175 | -0.0034 | 0.0034 | NA |
| line_129 | Abdomen | 10 | 1.1160 | 0.0606 | 5.4292 | 10 | 1.1380 | 0.0516 | 4.5340 | 0.0020 | 0.0020 | NA |
| line_129 | Thorax | 10 | 0.5522 | 0.0468 | 8.4830 | 10 | 0.5600 | 0.0472 | 8.4300 | 0.0007 | 0.0007 | NA |
| line_136 | Abdomen | 16 | 1.1244 | 0.0917 | 8.1576 | 11 | 0.9991 | 0.0549 | 5.4905 | -0.0114 | 0.0114 | NA |
| line_136 | Thorax | 15 | 0.5675 | 0.0382 | 6.7257 | 11 | 0.5218 | 0.0533 | 10.2061 | -0.0042 | 0.0042 | NA |
| line_138 | Thorax | 14 | 0.6285 | 0.0452 | 7.1999 | 8 | 0.5986 | 0.0261 | 4.3596 | -0.0027 | 0.0027 | NA |
| line_138 | Abdomen | 14 | 1.0886 | 0.0616 | 5.6612 | 8 | 0.9950 | 0.0701 | 7.0454 | -0.0085 | 0.0085 | NA |
| line_142 | Thorax | 10 | 0.6710 | 0.0431 | 6.4178 | 21 | 0.6274 | 0.0393 | 6.2606 | -0.0040 | 0.0040 | NA |
| line_142 | Abdomen | 10 | 1.1090 | 0.0633 | 5.7101 | 21 | 0.9810 | 0.0944 | 9.6221 | -0.0116 | 0.0116 | NA |
| line_149 | Thorax | 5 | 0.6480 | 0.0466 | 7.1888 | 16 | 0.5563 | 0.0398 | 7.1572 | -0.0083 | 0.0083 | NA |
| line_149 | Abdomen | 5 | 1.1220 | 0.0991 | 8.8321 | 16 | 1.0481 | 0.0799 | 7.6265 | -0.0067 | 0.0067 | NA |
| line_153 | Thorax | 7 | 0.6029 | 0.0359 | 5.9588 | 11 | 0.6355 | 0.0391 | 6.1500 | 0.0030 | 0.0030 | NA |
| line_153 | Abdomen | 7 | 1.0986 | 0.0708 | 6.4458 | 11 | 1.1345 | 0.0813 | 7.1646 | 0.0033 | 0.0033 | NA |
| line_158 | Thorax | 25 | 0.6017 | 0.0366 | 6.0755 | 6 | 0.5983 | 0.0343 | 5.7330 | -0.0003 | 0.0003 | NA |
| line_158 | Abdomen | 25 | 1.0979 | 0.0870 | 7.9220 | 6 | 1.0733 | 0.0698 | 6.4995 | -0.0022 | 0.0022 | NA |
| line_161 | Abdomen | 13 | 1.1054 | 0.0996 | 9.0135 | 12 | 1.0842 | 0.0652 | 6.0093 | -0.0019 | 0.0019 | NA |
| line_161 | Thorax | 13 | 0.6123 | 0.0425 | 6.9339 | 12 | 0.6333 | 0.0542 | 8.5516 | 0.0019 | 0.0019 | NA |
| line_176 | Abdomen | 19 | 1.0500 | 0.0760 | 7.2392 | 12 | 0.9150 | 0.0535 | 5.8484 | -0.0123 | 0.0123 | NA |
| line_176 | Thorax | 19 | 0.5578 | 0.0525 | 9.4179 | 12 | 0.5133 | 0.0419 | 8.1528 | -0.0040 | 0.0040 | NA |
| line_177 | Thorax | 18 | 0.6072 | 0.0388 | 6.3858 | 8 | 0.5675 | 0.0271 | 4.7796 | -0.0036 | 0.0036 | NA |
| line_177 | Abdomen | 18 | 1.1600 | 0.0582 | 5.0180 | 8 | 1.0700 | 0.0553 | 5.1674 | -0.0082 | 0.0082 | NA |
| line_181 | Abdomen | 15 | 1.0820 | 0.0658 | 6.0826 | 33 | 0.9894 | 0.0629 | 6.3570 | -0.0084 | 0.0084 | NA |
| line_181 | Thorax | 15 | 0.6153 | 0.0336 | 5.4549 | 33 | 0.5691 | 0.0365 | 6.4119 | -0.0042 | 0.0042 | NA |
| line_189 | Thorax | 12 | 0.5850 | 0.0309 | 5.2813 | 20 | 0.5640 | 0.0481 | 8.5207 | -0.0019 | 0.0019 | MR4 (minor) B |
| line_189 | Abdomen | 12 | 1.1358 | 0.0903 | 7.9500 | 20 | 0.8245 | 0.1113 | 13.5028 | -0.0283 | 0.0283 | MR4 (minor) B |
| line_195 | Abdomen | 10 | 0.9800 | 0.0750 | 7.6512 | 14 | 1.1014 | 0.0493 | 4.4742 | 0.0110 | 0.0110 | NA |
| line_195 | Thorax | 10 | 0.5830 | 0.0337 | 5.7773 | 14 | 0.5585 | 0.0460 | 8.2332 | -0.0022 | 0.0022 | NA |
| line_208 | Abdomen | 10 | 1.1320 | 0.0770 | 6.8021 | 6 | 1.1667 | 0.0361 | 3.0984 | 0.0032 | 0.0032 | NA |
| line_208 | Thorax | 10 | 0.6450 | 0.0327 | 5.0767 | 6 | 0.6200 | 0.0335 | 5.3978 | -0.0023 | 0.0023 | NA |
| line_21 | Abdomen | 13 | 1.1569 | 0.0605 | 5.2266 | 15 | 1.0167 | 0.0719 | 7.0701 | -0.0128 | 0.0128 | MR3 (minor) A |
| line_21 | Thorax | 14 | 0.5918 | 0.0379 | 6.4039 | 15 | 0.5571 | 0.0429 | 7.6936 | -0.0032 | 0.0032 | MR3 (minor) A |
| line_217 | Thorax | 7 | 0.5986 | 0.0273 | 4.5680 | 12 | 0.5590 | 0.0370 | 6.6106 | -0.0036 | 0.0036 | MR4 (minor) B |
| line_217 | Abdomen | 7 | 1.1567 | 0.0513 | 4.4309 | 12 | 1.0117 | 0.0727 | 7.1879 | -0.0132 | 0.0132 | MR4 (minor) B |
| line_223 | Abdomen | NA | NA | NA | NA | 7 | 1.1043 | 0.0577 | 5.2245 | NA | NA | MR3 (major) B / MR2 (major) A |
| line_223 | Thorax | NA | NA | NA | NA | 7 | 0.5743 | 0.0315 | 5.4933 | NA | NA | MR3 (major) B / MR2 (major) A |
| line_227 | Abdomen | 5 | 0.9620 | 0.0614 | 6.3826 | 11 | 0.8464 | 0.0825 | 9.7470 | -0.0105 | 0.0105 | MR1 (minor) B |
| line_227 | Thorax | 5 | 0.5940 | 0.0439 | 7.3959 | 11 | 0.4680 | 0.0702 | 15.0013 | -0.0115 | 0.0115 | MR1 (minor) B |
| line_228 | Thorax | 27 | 0.6083 | 0.0338 | 5.5622 | 22 | 0.5495 | 0.0334 | 6.0758 | -0.0053 | 0.0053 | NA |
| line_228 | Abdomen | 27 | 1.1752 | 0.0942 | 8.0151 | 22 | 1.0314 | 0.1069 | 10.3623 | -0.0131 | 0.0131 | NA |
| line_229 | Thorax | 7 | 0.5857 | 0.0707 | 12.0668 | 7 | 0.5600 | 0.0238 | 4.2509 | -0.0023 | 0.0023 | MR4 (major) A |
| line_229 | Abdomen | 7 | 0.9871 | 0.0916 | 9.2793 | 7 | 1.0100 | 0.0668 | 6.6171 | 0.0021 | 0.0021 | MR4 (major) A |
| line_233 | Thorax | 15 | 0.5950 | 0.0435 | 7.3036 | 18 | 0.5613 | 0.0370 | 6.5988 | -0.0031 | 0.0031 | NA |
| line_233 | Abdomen | 15 | 1.0280 | 0.0890 | 8.6555 | 18 | 0.9194 | 0.0733 | 7.9703 | -0.0099 | 0.0099 | NA |
| line_235 | Thorax | 31 | 0.5886 | 0.0638 | 10.8376 | 12 | 0.5692 | 0.0334 | 5.8731 | -0.0018 | 0.0018 | NA |
| line_235 | Abdomen | 18 | 1.1656 | 0.1010 | 8.6661 | 12 | 1.2492 | 0.1033 | 8.2664 | 0.0076 | 0.0076 | NA |
| line_237 | Thorax | 7 | 0.6000 | 0.0321 | 5.3576 | 14 | 0.5421 | 0.0402 | 7.4198 | -0.0053 | 0.0053 | NA |
| line_237 | Abdomen | 7 | 1.1886 | 0.1281 | 10.7792 | 14 | 1.0436 | 0.0945 | 9.0565 | -0.0132 | 0.0132 | NA |
| line_239 | Thorax | 26 | 0.6017 | 0.0451 | 7.4919 | 12 | 0.5982 | 0.0303 | 5.0606 | -0.0003 | 0.0003 | NA |
| line_239 | Abdomen | 26 | 1.1019 | 0.1398 | 12.6869 | 12 | 0.9933 | 0.0907 | 9.1296 | -0.0099 | 0.0099 | NA |
| line_256 | Abdomen | 11 | 1.2100 | 0.0899 | 7.4288 | 9 | 1.0089 | 0.0352 | 3.4849 | -0.0183 | 0.0183 | NA |
| line_256 | Thorax | 11 | 0.6100 | 0.0516 | 8.4655 | 9 | 0.5911 | 0.0366 | 6.1838 | -0.0017 | 0.0017 | NA |
| line_26 | Abdomen | 6 | 1.2567 | 0.1061 | 8.4465 | 12 | 0.8425 | 0.0477 | 5.6614 | -0.0377 | 0.0377 | NA |
| line_26 | Thorax | 6 | 0.6283 | 0.0313 | 4.9737 | 12 | 0.5264 | 0.0454 | 8.6342 | -0.0093 | 0.0093 | NA |
| line_28 | Abdomen | 8 | 1.0700 | 0.0648 | 6.0568 | 14 | 1.0450 | 0.0785 | 7.5139 | -0.0023 | 0.0023 | MR4 (major) B |
| line_28 | Thorax | 8 | 0.6138 | 0.0403 | 6.5716 | 14 | 0.5364 | 0.0462 | 8.6084 | -0.0070 | 0.0070 | MR4 (major) B |
| line_280 | Abdomen | 12 | 1.0442 | 0.0558 | 5.3472 | 21 | 1.0288 | 0.0755 | 7.3421 | -0.0014 | 0.0014 | NA |
| line_280 | Thorax | 15 | 0.6269 | 0.0357 | 5.6913 | 21 | 0.5684 | 0.0536 | 9.4241 | -0.0053 | 0.0053 | NA |
| line_287 | Abdomen | 12 | 1.2450 | 0.1241 | 9.9706 | 25 | 1.0384 | 0.0749 | 7.2155 | -0.0188 | 0.0188 | NA |
| line_287 | Thorax | 13 | 0.6192 | 0.0272 | 4.3961 | 25 | 0.5492 | 0.0449 | 8.1755 | -0.0064 | 0.0064 | NA |
| line_301 | Thorax | 6 | 0.6133 | 0.0280 | 4.5730 | 8 | 0.5688 | 0.0236 | 4.1435 | -0.0041 | 0.0041 | NA |
| line_301 | Abdomen | 6 | 1.0133 | 0.0528 | 5.2094 | 7 | 1.1529 | 0.1107 | 9.6033 | 0.0127 | 0.0127 | NA |
| line_303 | Thorax | 17 | 0.6244 | 0.0459 | 7.3504 | 7 | 0.5629 | 0.0281 | 4.9951 | -0.0056 | 0.0056 | NA |
| line_303 | Abdomen | 17 | 1.0706 | 0.0621 | 5.8002 | 7 | 1.0871 | 0.0920 | 8.4591 | 0.0015 | 0.0015 | NA |
| line_304 | Thorax | 11 | 0.6260 | 0.0389 | 6.2189 | 17 | 0.6094 | 0.0344 | 5.6389 | -0.0015 | 0.0015 | MR3 (major) / MR1 (minor) / MR4 (minor) B / MR2 (major) B |
| line_304 | Abdomen | 11 | 1.1891 | 0.0667 | 5.6095 | 17 | 1.1729 | 0.1508 | 12.8549 | -0.0015 | 0.0015 | MR3 (major) / MR1 (minor) / MR4 (minor) B / MR2 (major) B |
| line_306 | Thorax | 10 | 0.5940 | 0.0372 | 6.2590 | 11 | 0.5711 | 0.0434 | 7.6044 | -0.0021 | 0.0021 | NA |
| line_306 | Abdomen | 10 | 1.1690 | 0.1032 | 8.8298 | 11 | 1.2145 | 0.0992 | 8.1704 | 0.0041 | 0.0041 | NA |
| line_307 | Thorax | 27 | 0.5658 | 0.0395 | 6.9842 | 19 | 0.5328 | 0.0301 | 5.6421 | -0.0030 | 0.0030 | NA |
| line_307 | Abdomen | 27 | 1.1533 | 0.1008 | 8.7403 | 19 | 1.0311 | 0.0635 | 6.1587 | -0.0111 | 0.0111 | NA |
| line_309 | Abdomen | 9 | 1.0844 | 0.0585 | 5.3988 | 7 | 1.0943 | 0.0824 | 7.5331 | 0.0009 | 0.0009 | NA |
| line_309 | Thorax | 9 | 0.6244 | 0.0207 | 3.3122 | 7 | 0.5757 | 0.0472 | 8.1999 | -0.0044 | 0.0044 | NA |
| line_31 | Thorax | 12 | 0.5733 | 0.0235 | 4.0961 | 7 | 0.5443 | 0.0276 | 5.0713 | -0.0026 | 0.0026 | NA |
| line_31 | Abdomen | 12 | 1.2825 | 0.1175 | 9.1605 | 7 | 0.9957 | 0.0824 | 8.2788 | -0.0261 | 0.0261 | NA |
| line_310 | Thorax | 5 | 0.5580 | 0.0409 | 7.3236 | 16 | 0.5158 | 0.0297 | 5.7543 | -0.0038 | 0.0038 | NA |
| line_310 | Abdomen | 5 | 1.0760 | 0.0666 | 6.1857 | 16 | 1.1181 | 0.0966 | 8.6416 | 0.0038 | 0.0038 | NA |
| line_313 | Thorax | 8 | 0.5525 | 0.0477 | 8.6397 | 17 | 0.5369 | 0.0255 | 4.7473 | -0.0014 | 0.0014 | NA |
| line_313 | Abdomen | 8 | 1.0463 | 0.0537 | 5.1328 | 17 | 0.9412 | 0.1094 | 11.6202 | -0.0096 | 0.0096 | NA |
| line_315 | Abdomen | NA | NA | NA | NA | 11 | 1.2418 | 0.0810 | 6.5204 | NA | NA | no |
| line_315 | Thorax | NA | NA | NA | NA | 11 | 0.5236 | 0.0353 | 6.7396 | NA | NA | no |
| line_317 | Abdomen | 14 | 1.1371 | 0.0461 | 4.0583 | 13 | 1.0077 | 0.0398 | 3.9519 | -0.0118 | 0.0118 | NA |
| line_317 | Thorax | 14 | 0.5731 | 0.0317 | 5.5357 | 13 | 0.5533 | 0.0423 | 7.6416 | -0.0018 | 0.0018 | NA |
| line_318 | Thorax | 25 | 0.6125 | 0.0300 | 4.8920 | 12 | 0.4855 | 0.0202 | 4.1571 | -0.0115 | 0.0115 | NA |
| line_318 | Abdomen | 25 | 1.0908 | 0.0905 | 8.2970 | 12 | 0.9675 | 0.0603 | 6.2347 | -0.0112 | 0.0112 | NA |
| line_319 | Thorax | 14 | 0.5721 | 0.0258 | 4.5048 | 9 | 0.5511 | 0.0362 | 6.5702 | -0.0019 | 0.0019 | NA |
| line_319 | Abdomen | 14 | 1.0957 | 0.0426 | 3.8838 | 9 | 1.0344 | 0.1367 | 13.2116 | -0.0056 | 0.0056 | NA |
| line_32 | Thorax | 5 | 0.5440 | 0.0358 | 6.5767 | 8 | 0.5625 | 0.0381 | 6.7696 | 0.0017 | 0.0017 | MR3 (major) A |
| line_32 | Abdomen | 5 | 1.1860 | 0.0631 | 5.3193 | 8 | 1.0963 | 0.1448 | 13.2095 | -0.0082 | 0.0082 | MR3 (major) A |
| line_320 | Thorax | 11 | 0.5700 | 0.0434 | 7.6068 | 14 | 0.5292 | 0.0272 | 5.1437 | -0.0037 | 0.0037 | NA |
| line_320 | Abdomen | 11 | 1.0382 | 0.0691 | 6.6569 | 14 | 0.9457 | 0.0788 | 8.3334 | -0.0084 | 0.0084 | NA |
| line_321 | Thorax | 21 | 0.6014 | 0.0414 | 6.8814 | 24 | 0.5558 | 0.0283 | 5.0863 | -0.0041 | 0.0041 | MR3 (minor) B / MR1 (major) B / MR4 (major) B |
| line_321 | Abdomen | 21 | 1.1419 | 0.0760 | 6.6557 | 24 | 1.0304 | 0.0636 | 6.1743 | -0.0101 | 0.0101 | MR3 (minor) B / MR1 (major) B / MR4 (major) B |
| line_324 | Thorax | 15 | 0.5773 | 0.0271 | 4.6966 | 14 | 0.5550 | 0.0288 | 5.1813 | -0.0020 | 0.0020 | MR3 (major) B / MR1 (major) / MR4 (minor) A / MR2 (major) A |
| line_324 | Abdomen | 15 | 1.0440 | 0.0564 | 5.4039 | 14 | 1.0329 | 0.0595 | 5.7646 | -0.0010 | 0.0010 | MR3 (major) B / MR1 (major) / MR4 (minor) A / MR2 (major) A |
| line_332 | Thorax | 11 | 0.5782 | 0.0360 | 6.2273 | 7 | 0.5071 | 0.0377 | 7.4404 | -0.0065 | 0.0065 | NA |
| line_332 | Abdomen | 11 | 1.0864 | 0.0668 | 6.1512 | 7 | 1.1286 | 0.0919 | 8.1440 | 0.0038 | 0.0038 | NA |
| line_335 | Thorax | 12 | 0.5850 | 0.0366 | 6.2489 | 11 | 0.5163 | 0.0207 | 4.0017 | -0.0063 | 0.0063 | NA |
| line_335 | Abdomen | 12 | 1.1183 | 0.0677 | 6.0507 | 11 | 0.9327 | 0.0855 | 9.1614 | -0.0169 | 0.0169 | NA |
| line_336 | Thorax | 6 | 0.6083 | 0.0098 | 1.6162 | 18 | 0.5678 | 0.0304 | 5.3543 | -0.0037 | 0.0037 | NA |
| line_336 | Abdomen | 6 | 1.2500 | 0.0089 | 0.7155 | 18 | 0.9594 | 0.0616 | 6.4197 | -0.0264 | 0.0264 | NA |
| line_338 | Abdomen | 9 | 1.1067 | 0.0927 | 8.3798 | 15 | 1.0780 | 0.0986 | 9.1443 | -0.0026 | 0.0026 | NA |
| line_338 | Thorax | 9 | 0.5278 | 0.0338 | 6.4098 | 15 | 0.6107 | 0.0561 | 9.1901 | 0.0075 | 0.0075 | NA |
| line_340 | Abdomen | 6 | 1.1050 | 0.0281 | 2.5436 | 6 | 1.4133 | 0.0280 | 1.9845 | 0.0280 | 0.0280 | NA |
| line_340 | Thorax | 6 | 0.5780 | 0.0192 | 3.3279 | 6 | 0.5683 | 0.0133 | 2.3387 | -0.0009 | 0.0009 | NA |
| line_348 | Thorax | 8 | 0.5563 | 0.0396 | 7.1225 | 11 | 0.5327 | 0.0300 | 5.6371 | -0.0021 | 0.0021 | NA |
| line_348 | Abdomen | 8 | 0.9800 | 0.0697 | 7.1116 | 11 | 1.0800 | 0.0856 | 7.9219 | 0.0091 | 0.0091 | NA |
| line_350 | Thorax | 6 | 0.5700 | 0.0369 | 6.4699 | 7 | 0.5400 | 0.0346 | 6.4150 | -0.0027 | 0.0027 | NA |
| line_350 | Abdomen | 6 | 0.9850 | 0.0339 | 3.4428 | 7 | 1.0514 | 0.0752 | 7.1475 | 0.0060 | 0.0060 | NA |
| line_352 | Thorax | 35 | 0.6057 | 0.0501 | 8.2672 | 7 | 0.5943 | 0.0369 | 6.2098 | -0.0010 | 0.0010 | NA |
| line_352 | Abdomen | 35 | 1.1154 | 0.0843 | 7.5584 | 7 | 1.1071 | 0.1072 | 9.6820 | -0.0008 | 0.0008 | NA |
| line_354 | Thorax | 9 | 0.5814 | 0.0339 | 5.8264 | 21 | 0.5729 | 0.0321 | 5.6063 | -0.0008 | 0.0008 | no |
| line_354 | Abdomen | 9 | 1.0878 | 0.0997 | 9.1675 | 21 | 1.0305 | 0.1124 | 10.9123 | -0.0052 | 0.0052 | no |
| line_356 | Thorax | 14 | 0.5843 | 0.0396 | 6.7703 | 22 | 0.5289 | 0.0387 | 7.3192 | -0.0050 | 0.0050 | NA |
| line_356 | Abdomen | 14 | 1.1079 | 0.0996 | 8.9929 | 22 | 1.2023 | 0.1052 | 8.7535 | 0.0086 | 0.0086 | NA |
| line_357 | Thorax | 6 | 0.6017 | 0.0643 | 10.6898 | 8 | 0.6083 | 0.0183 | 3.0162 | 0.0006 | 0.0006 | NA |
| line_357 | Abdomen | 6 | 1.0867 | 0.0975 | 8.9726 | 8 | 1.0771 | 0.0541 | 5.0200 | -0.0009 | 0.0009 | NA |
| line_358 | Thorax | 13 | 0.5638 | 0.0380 | 6.7355 | 17 | 0.5335 | 0.0412 | 7.7263 | -0.0028 | 0.0028 | NA |
| line_358 | Abdomen | 13 | 0.9900 | 0.0610 | 6.1580 | 17 | 1.1635 | 0.1581 | 13.5856 | 0.0158 | 0.0158 | NA |
| line_359 | Thorax | 31 | 0.5868 | 0.0445 | 7.5883 | 35 | 0.5700 | 0.0331 | 5.8010 | -0.0015 | 0.0015 | MR1 (major) B |
| line_359 | Abdomen | 21 | 1.2581 | 0.1289 | 10.2490 | 35 | 1.0677 | 0.0964 | 9.0294 | -0.0173 | 0.0173 | MR1 (major) B |
| line_360 | Abdomen | 14 | 1.0071 | 0.0882 | 8.7555 | 13 | 0.9938 | 0.0496 | 4.9895 | -0.0012 | 0.0012 | MR3 (major) A |
| line_360 | Thorax | 14 | 0.6046 | 0.0472 | 7.8050 | 13 | 0.5733 | 0.0436 | 7.6027 | -0.0028 | 0.0028 | MR3 (major) A |
| line_361 | Abdomen | 11 | 1.1782 | 0.0800 | 6.7882 | 13 | 1.0777 | 0.0777 | 7.2090 | -0.0091 | 0.0091 | NA |
| line_361 | Thorax | 11 | 0.5673 | 0.0338 | 5.9567 | 13 | 0.5367 | 0.0440 | 8.1931 | -0.0028 | 0.0028 | NA |
| line_362 | Abdomen | 14 | 1.2500 | 0.1398 | 11.1868 | 14 | 1.0486 | 0.0642 | 6.1221 | -0.0183 | 0.0183 | MR3 (minor) B / MR4 (major) A |
| line_362 | Thorax | 14 | 0.5879 | 0.0297 | 5.0453 | 14 | 0.5491 | 0.0497 | 9.0495 | -0.0035 | 0.0035 | MR3 (minor) B / MR4 (major) A |
| line_365 | Abdomen | 6 | 1.0667 | 0.1488 | 13.9516 | 9 | 1.0856 | 0.1043 | 9.6077 | 0.0017 | 0.0017 | MR4 (major) B |
| line_365 | Thorax | 6 | 0.5700 | 0.0443 | 7.7670 | 9 | 0.4917 | 0.0598 | 12.1638 | -0.0071 | 0.0071 | MR4 (major) B |
| line_367 | Thorax | 29 | 0.5848 | 0.0508 | 8.6783 | 10 | 0.5520 | 0.0282 | 5.1097 | -0.0030 | 0.0030 | NA |
| line_367 | Abdomen | 29 | 1.2431 | 0.1181 | 9.5015 | 10 | 1.0950 | 0.1023 | 9.3456 | -0.0135 | 0.0135 | NA |
| line_370 | Thorax | 11 | 0.5530 | 0.0362 | 6.5506 | 12 | 0.5592 | 0.0281 | 5.0271 | 0.0006 | 0.0006 | MR3 (major) A / MR1 (minor) A / MR4 (minor) A / MR2 (minor) A |
| line_370 | Abdomen | 11 | 1.1445 | 0.0874 | 7.6405 | 12 | 1.1908 | 0.1117 | 9.3816 | 0.0042 | 0.0042 | MR3 (major) A / MR1 (minor) A / MR4 (minor) A / MR2 (minor) A |
| line_371 | Thorax | 10 | 0.6180 | 0.0478 | 7.7340 | 16 | 0.5813 | 0.0346 | 5.9556 | -0.0033 | 0.0033 | MR3 (minor) B |
| line_371 | Abdomen | 10 | 0.9550 | 0.0654 | 6.8442 | 16 | 1.0188 | 0.0718 | 7.0500 | 0.0058 | 0.0058 | MR3 (minor) B |
| line_373 | Abdomen | 10 | 1.0630 | 0.0814 | 7.6561 | 27 | 1.0633 | 0.0786 | 7.3958 | 0.0000 | 0.0000 | no |
| line_373 | Thorax | 10 | 0.5489 | 0.0330 | 6.0042 | 27 | 0.5522 | 0.0434 | 7.8507 | 0.0003 | 0.0003 | no |
| line_374 | Abdomen | 11 | 1.1473 | 0.0801 | 6.9849 | 18 | 1.0800 | 0.0910 | 8.4254 | -0.0061 | 0.0061 | MR1 (minor) B |
| line_374 | Thorax | 11 | 0.5945 | 0.0273 | 4.5978 | 18 | 0.5957 | 0.0543 | 9.1166 | 0.0001 | 0.0001 | MR1 (minor) B |
| line_375 | Abdomen | 13 | 1.1400 | 0.1040 | 9.1231 | 14 | 1.0721 | 0.0765 | 7.1332 | -0.0062 | 0.0062 | NA |
| line_375 | Thorax | 13 | 0.6042 | 0.0215 | 3.5610 | 14 | 0.5900 | 0.0520 | 8.8070 | -0.0013 | 0.0013 | NA |
| line_377 | Abdomen | 12 | 1.0875 | 0.0717 | 6.5975 | 13 | 1.0462 | 0.0580 | 5.5400 | -0.0038 | 0.0038 | NA |
| line_377 | Thorax | 13 | 0.5854 | 0.0631 | 10.7729 | 13 | 0.5467 | 0.0394 | 7.2054 | -0.0035 | 0.0035 | NA |
| line_379 | Abdomen | 14 | 1.0421 | 0.1130 | 10.8443 | 13 | 0.9869 | 0.0497 | 5.0389 | -0.0050 | 0.0050 | NA |
| line_379 | Thorax | 16 | 0.5807 | 0.0420 | 7.2327 | 13 | 0.6062 | 0.0415 | 6.8532 | 0.0023 | 0.0023 | NA |
| line_38 | Thorax | 7 | 0.6200 | 0.0516 | 8.3290 | 15 | 0.5753 | 0.0350 | 6.0876 | -0.0041 | 0.0041 | NA |
| line_38 | Abdomen | 7 | 1.1586 | 0.0708 | 6.1120 | 15 | 1.2080 | 0.1194 | 9.8854 | 0.0045 | 0.0045 | NA |
| line_380 | Thorax | 13 | 0.5873 | 0.0598 | 10.1909 | 13 | 0.5300 | 0.0305 | 5.7455 | -0.0052 | 0.0052 | MR1 (minor) A |
| line_380 | Abdomen | 13 | 1.1177 | 0.0676 | 6.0478 | 13 | 0.9692 | 0.0599 | 6.1828 | -0.0135 | 0.0135 | MR1 (minor) A |
| line_381 | Thorax | 5 | 0.5920 | 0.0164 | 2.7756 | 10 | 0.5710 | 0.0341 | 5.9790 | -0.0019 | 0.0019 | NA |
| line_381 | Abdomen | 5 | 1.2500 | 0.0100 | 0.8000 | 10 | 0.9340 | 0.1095 | 11.7198 | -0.0287 | 0.0287 | NA |
| line_382 | Abdomen | 13 | 1.1946 | 0.0454 | 3.7996 | 6 | 0.9867 | 0.0723 | 7.3273 | -0.0189 | 0.0189 | NA |
| line_382 | Thorax | 13 | 0.6046 | 0.0355 | 5.8715 | 6 | 0.6020 | 0.0444 | 7.3729 | -0.0002 | 0.0002 | NA |
| line_383 | Abdomen | 15 | 1.0813 | 0.0805 | 7.4466 | 6 | 0.9567 | 0.0423 | 4.4184 | -0.0113 | 0.0113 | NA |
| line_383 | Thorax | 15 | 0.5957 | 0.0306 | 5.1304 | 6 | 0.5460 | 0.0305 | 5.5853 | -0.0045 | 0.0045 | NA |
| line_385 | Thorax | 11 | 0.5750 | 0.0321 | 5.5755 | 22 | 0.5868 | 0.0460 | 7.8425 | 0.0011 | 0.0011 | NA |
| line_385 | Abdomen | 11 | 1.0227 | 0.0963 | 9.4201 | 22 | 1.0540 | 0.1197 | 11.3577 | 0.0028 | 0.0028 | NA |
| line_386 | Abdomen | 12 | 1.3008 | 0.0950 | 7.3036 | 5 | 1.1000 | 0.0977 | 8.8840 | -0.0183 | 0.0183 | MR3 (minor) A |
| line_386 | Thorax | 12 | 0.5845 | 0.0270 | 4.6135 | 5 | 0.5720 | 0.0602 | 10.5186 | -0.0011 | 0.0011 | MR3 (minor) A |
| line_390 | Abdomen | 8 | 1.0975 | 0.1146 | 10.4429 | 9 | 1.0922 | 0.0628 | 5.7502 | -0.0005 | 0.0005 | NA |
| line_390 | Thorax | 8 | 0.6638 | 0.0374 | 5.6336 | 9 | 0.5889 | 0.0401 | 6.8160 | -0.0068 | 0.0068 | NA |
| line_391 | Thorax | NA | NA | NA | NA | 12 | 0.5600 | 0.0335 | 5.9761 | NA | NA | NA |
| line_391 | Abdomen | NA | NA | NA | NA | 11 | 0.9773 | 0.0656 | 6.7114 | NA | NA | NA |
| line_392 | Abdomen | 19 | 1.1758 | 0.0568 | 4.8304 | 11 | 1.0000 | 0.0605 | 6.0498 | -0.0160 | 0.0160 | NA |
| line_392 | Thorax | 19 | 0.5728 | 0.0306 | 5.3496 | 11 | 0.5430 | 0.0392 | 7.2140 | -0.0027 | 0.0027 | NA |
| line_395 | Abdomen | 13 | 1.0500 | 0.0572 | 5.4433 | 5 | 1.0000 | 0.0346 | 3.4641 | -0.0045 | 0.0045 | NA |
| line_395 | Thorax | 13 | 0.5738 | 0.0461 | 8.0343 | 5 | 0.5400 | 0.0515 | 9.5330 | -0.0031 | 0.0031 | NA |
| line_397 | Thorax | NA | NA | NA | NA | 12 | 0.6000 | 0.0167 | 2.7889 | NA | NA | NA |
| line_397 | Abdomen | NA | NA | NA | NA | 12 | 0.9842 | 0.0706 | 7.1777 | NA | NA | NA |
| line_399 | Thorax | 11 | 0.5891 | 0.0339 | 5.7543 | 11 | 0.5500 | 0.0522 | 9.4912 | -0.0036 | 0.0036 | NA |
| line_399 | Abdomen | 11 | 1.1818 | 0.1056 | 8.9374 | 11 | 1.0073 | 0.1110 | 11.0202 | -0.0159 | 0.0159 | NA |
| line_40 | Abdomen | 11 | 1.0300 | 0.1342 | 13.0337 | 20 | 1.0390 | 0.0871 | 8.3784 | 0.0008 | 0.0008 | NA |
| line_40 | Thorax | 11 | 0.6350 | 0.0232 | 3.6557 | 20 | 0.5505 | 0.0509 | 9.2517 | -0.0077 | 0.0077 | NA |
| line_405 | Thorax | 10 | 0.5900 | 0.0427 | 7.2352 | 11 | 0.5540 | 0.0486 | 8.7689 | -0.0033 | 0.0033 | NA |
| line_405 | Abdomen | 10 | 1.0700 | 0.1017 | 9.5003 | 10 | 1.1300 | 0.1458 | 12.9054 | 0.0055 | 0.0055 | NA |
| line_406 | Abdomen | 13 | 1.1400 | 0.0719 | 6.3052 | 13 | 0.9308 | 0.0750 | 8.0574 | -0.0190 | 0.0190 | MR2 (major) B |
| line_406 | Thorax | 13 | 0.6131 | 0.0440 | 7.1838 | 13 | 0.5085 | 0.0452 | 8.8991 | -0.0095 | 0.0095 | MR2 (major) B |
| line_409 | Thorax | 13 | 0.6055 | 0.0266 | 4.3925 | 11 | 0.5888 | 0.0217 | 3.6809 | -0.0015 | 0.0015 | NA |
| line_409 | Abdomen | 13 | 1.1238 | 0.0684 | 6.0843 | 11 | 1.3882 | 0.1389 | 10.0067 | 0.0240 | 0.0240 | NA |
| line_41 | Abdomen | 7 | 1.2971 | 0.0871 | 6.7166 | 15 | 1.0720 | 0.0662 | 6.1797 | -0.0205 | 0.0205 | NA |
| line_41 | Thorax | 20 | 0.5785 | 0.0507 | 8.7655 | 15 | 0.5436 | 0.0348 | 6.3976 | -0.0032 | 0.0032 | NA |
| line_42 | Thorax | 5 | 0.6220 | 0.0476 | 7.6599 | 6 | 0.4967 | 0.0266 | 5.3523 | -0.0114 | 0.0114 | NA |
| line_42 | Abdomen | 5 | 1.0900 | 0.0972 | 8.9185 | 6 | 0.9133 | 0.0612 | 6.7018 | -0.0161 | 0.0161 | NA |
| line_426 | Thorax | 12 | 0.5909 | 0.0359 | 6.0760 | 21 | 0.5945 | 0.0312 | 5.2481 | 0.0003 | 0.0003 | NA |
| line_426 | Abdomen | 12 | 1.0117 | 0.1107 | 10.9410 | 21 | 1.1743 | 0.0938 | 7.9911 | 0.0148 | 0.0148 | NA |
| line_427 | Thorax | 11 | 0.5864 | 0.0423 | 7.2062 | 15 | 0.6043 | 0.0413 | 6.8297 | 0.0016 | 0.0016 | NA |
| line_427 | Abdomen | 11 | 1.1300 | 0.0681 | 6.0281 | 15 | 1.0729 | 0.0886 | 8.2598 | -0.0052 | 0.0052 | NA |
| line_437 | Thorax | 23 | 0.6152 | 0.0475 | 7.7159 | 12 | 0.5775 | 0.0373 | 6.4625 | -0.0034 | 0.0034 | NA |
| line_437 | Abdomen | 23 | 1.1917 | 0.0429 | 3.6016 | 12 | 0.9720 | 0.1006 | 10.3496 | -0.0200 | 0.0200 | NA |
| line_439 | Thorax | 23 | 0.5581 | 0.0386 | 6.9076 | 30 | 0.5314 | 0.0304 | 5.7287 | -0.0024 | 0.0024 | NA |
| line_439 | Abdomen | 23 | 1.1765 | 0.0760 | 6.4610 | 30 | 1.0000 | 0.0935 | 9.3497 | -0.0160 | 0.0160 | NA |
| line_440 | Thorax | 11 | 0.5940 | 0.0544 | 9.1592 | 24 | 0.5609 | 0.0373 | 6.6476 | -0.0030 | 0.0030 | NA |
| line_440 | Abdomen | 11 | 1.0455 | 0.1009 | 9.6544 | 23 | 1.0568 | 0.0935 | 8.8447 | 0.0010 | 0.0010 | NA |
| line_441 | Thorax | 6 | 0.5567 | 0.0361 | 6.4936 | 18 | 0.5688 | 0.0348 | 6.1179 | 0.0011 | 0.0011 | MR1 (major) A |
| line_441 | Abdomen | 6 | 0.9750 | 0.0589 | 6.0417 | 18 | 1.0678 | 0.0972 | 9.1058 | 0.0084 | 0.0084 | MR1 (major) A |
| line_443 | Thorax | 13 | 0.6300 | 0.0349 | 5.5366 | 9 | 0.5878 | 0.0205 | 3.4844 | -0.0038 | 0.0038 | NA |
| line_443 | Abdomen | 13 | 1.1746 | 0.0608 | 5.1740 | 9 | 1.1644 | 0.1616 | 13.8747 | -0.0009 | 0.0009 | NA |
| line_45 | Abdomen | 15 | 1.3767 | 0.1060 | 7.7005 | 5 | 1.0100 | 0.0100 | 0.9901 | -0.0333 | 0.0333 | NA |
| line_45 | Thorax | 15 | 0.5960 | 0.0444 | 7.4444 | 5 | 0.5600 | 0.0071 | 1.2627 | -0.0033 | 0.0033 | NA |
| line_461 | Abdomen | 5 | 0.9540 | 0.0055 | 0.5741 | 9 | 1.0222 | 0.0556 | 5.4418 | 0.0062 | 0.0062 | NA |
| line_461 | Thorax | 5 | 0.5260 | 0.0439 | 8.3520 | 9 | 0.5244 | 0.0292 | 5.5682 | -0.0001 | 0.0001 | NA |
| line_48 | Thorax | 8 | 0.5457 | 0.0237 | 4.3438 | 21 | 0.5515 | 0.0298 | 5.4006 | 0.0005 | 0.0005 | NA |
| line_48 | Abdomen | 7 | 1.2186 | 0.0865 | 7.0979 | 21 | 1.0510 | 0.0943 | 8.9761 | -0.0152 | 0.0152 | NA |
| line_486 | Thorax | 5 | 0.6220 | 0.0277 | 4.4612 | 7 | 0.5486 | 0.0248 | 4.5181 | -0.0067 | 0.0067 | NA |
| line_486 | Abdomen | 5 | 1.1580 | 0.1089 | 9.4084 | 7 | 1.2186 | 0.0710 | 5.8303 | 0.0055 | 0.0055 | NA |
| line_49 | Abdomen | 25 | 1.1324 | 0.1143 | 10.0953 | 12 | 0.8933 | 0.0739 | 8.2719 | -0.0217 | 0.0217 | MR3 (minor) B |
| line_49 | Thorax | 25 | 0.5900 | 0.0533 | 9.0295 | 12 | 0.4809 | 0.0466 | 9.6845 | -0.0099 | 0.0099 | MR3 (minor) B |
| line_491 | Abdomen | 12 | 1.1525 | 0.0940 | 8.1574 | 6 | 1.3567 | 0.0501 | 3.6904 | 0.0186 | 0.0186 | MR3 (minor) A / MR1 (major) A / MR2 (major) A |
| line_491 | Thorax | 12 | 0.5917 | 0.0272 | 4.6052 | 6 | 0.5900 | 0.0387 | 6.5644 | -0.0002 | 0.0002 | MR3 (minor) AMR1 (major) AMR2 (major) A |
| line_502 | Abdomen | 14 | 1.1057 | 0.0989 | 8.9440 | 14 | 1.0857 | 0.0766 | 7.0582 | -0.0018 | 0.0018 | NA |
| line_502 | Thorax | 14 | 0.5954 | 0.0445 | 7.4679 | 14 | 0.5364 | 0.0452 | 8.4201 | -0.0054 | 0.0054 | NA |
| line_508 | Abdomen | 26 | 1.1112 | 0.1679 | 15.1115 | 27 | 1.0341 | 0.0888 | 8.5922 | -0.0070 | 0.0070 | NA |
| line_508 | Thorax | 26 | 0.5752 | 0.0381 | 6.6224 | 27 | 0.5488 | 0.0473 | 8.6129 | -0.0024 | 0.0024 | NA |
| line_509 | Thorax | 6 | 0.5100 | 0.0063 | 1.2401 | 17 | 0.5231 | 0.0275 | 5.2569 | 0.0012 | 0.0012 | NA |
| line_509 | Abdomen | 6 | 1.0583 | 0.0041 | 0.3857 | 17 | 1.0459 | 0.1211 | 11.5829 | -0.0011 | 0.0011 | NA |
| line_513 | Thorax | 25 | 0.6058 | 0.0336 | 5.5479 | 10 | 0.6010 | 0.0288 | 4.8000 | -0.0004 | 0.0004 | NA |
| line_513 | Abdomen | 25 | 1.1140 | 0.0886 | 7.9533 | 10 | 1.1650 | 0.0826 | 7.0927 | 0.0046 | 0.0046 | NA |
| line_517 | Thorax | 25 | 0.5430 | 0.0388 | 7.1414 | 10 | 0.5770 | 0.0350 | 6.0617 | 0.0031 | 0.0031 | SNP_MR2 (minor) |
| line_517 | Abdomen | 24 | 0.9667 | 0.0954 | 9.8691 | 10 | 0.9980 | 0.0684 | 6.8580 | 0.0028 | 0.0028 | SNP_MR2 (minor) |
| line_528 | Thorax | 11 | 0.6136 | 0.0388 | 6.3230 | 11 | 0.5864 | 0.0543 | 9.2557 | -0.0025 | 0.0025 | NA |
| line_528 | Abdomen | 11 | 1.0955 | 0.1218 | 11.1157 | 11 | 1.0091 | 0.0943 | 9.3433 | -0.0079 | 0.0079 | NA |
| line_530 | Thorax | 11 | 0.5550 | 0.0268 | 4.8235 | 8 | 0.5450 | 0.0288 | 5.2862 | -0.0009 | 0.0009 | NA |
| line_530 | Abdomen | 11 | 1.0627 | 0.0615 | 5.7867 | 8 | 0.9575 | 0.0875 | 9.1347 | -0.0096 | 0.0096 | NA |
| line_531 | Thorax | 7 | 0.5986 | 0.0038 | 0.6314 | 12 | 0.4927 | 0.0408 | 8.2734 | -0.0096 | 0.0096 | NA |
| line_531 | Abdomen | 7 | 1.0971 | 0.0621 | 5.6607 | 12 | 0.9942 | 0.1481 | 14.8976 | -0.0094 | 0.0094 | NA |
| line_535 | Thorax | 11 | 0.5909 | 0.0466 | 7.8817 | 8 | 0.5643 | 0.0326 | 5.7749 | -0.0024 | 0.0024 | NA |
| line_535 | Abdomen | 11 | 1.1345 | 0.0628 | 5.5377 | 8 | 1.1250 | 0.1084 | 9.6324 | -0.0009 | 0.0009 | NA |
| line_551 | Abdomen | 10 | 1.0900 | 0.0429 | 3.9401 | 13 | 1.0615 | 0.0806 | 7.5934 | -0.0026 | 0.0026 | NA |
| line_551 | Thorax | 10 | 0.5270 | 0.0442 | 8.3936 | 13 | 0.5254 | 0.0422 | 8.0234 | -0.0001 | 0.0001 | NA |
| line_555 | Thorax | 5 | 0.5960 | 0.0288 | 4.8338 | 8 | 0.5588 | 0.0391 | 6.9931 | -0.0034 | 0.0034 | MR4 (minor) A |
| line_555 | Abdomen | 5 | 1.2180 | 0.0672 | 5.5198 | 8 | 1.0825 | 0.1139 | 10.5183 | -0.0123 | 0.0123 | MR4 (minor) A |
| line_559 | Thorax | 7 | 0.6100 | 0.0183 | 2.9930 | 11 | 0.5527 | 0.0344 | 6.2196 | -0.0052 | 0.0052 | MR1 (minor) A |
| line_559 | Abdomen | 7 | 1.0757 | 0.0808 | 7.5112 | 11 | 0.9864 | 0.0772 | 7.8304 | -0.0081 | 0.0081 | MR1 (minor) A |
| line_563 | Abdomen | 14 | 1.1671 | 0.0799 | 6.8496 | 12 | 0.9383 | 0.0653 | 6.9637 | -0.0208 | 0.0208 | NA |
| line_563 | Thorax | 14 | 0.5769 | 0.0411 | 7.1251 | 12 | 0.4940 | 0.0389 | 7.8806 | -0.0075 | 0.0075 | NA |
| line_566 | Thorax | 6 | 0.5950 | 0.0295 | 4.9573 | 11 | 0.5080 | 0.0274 | 5.3950 | -0.0079 | 0.0079 | NA |
| line_566 | Abdomen | 6 | 0.9983 | 0.1251 | 12.5335 | 10 | 1.0440 | 0.0828 | 7.9270 | 0.0042 | 0.0042 | NA |
| line_57 | Thorax | 20 | 0.5800 | 0.0446 | 7.6891 | 14 | 0.5264 | 0.0447 | 8.4824 | -0.0049 | 0.0049 | NA |
| line_57 | Abdomen | 20 | 1.1380 | 0.1019 | 8.9550 | 14 | 1.1421 | 0.1359 | 11.9022 | 0.0004 | 0.0004 | NA |
| line_584 | Abdomen | 12 | 1.0667 | 0.0877 | 8.2249 | 9 | 1.1156 | 0.0522 | 4.6818 | 0.0044 | 0.0044 | NA |
| line_584 | Thorax | 12 | 0.5930 | 0.0359 | 6.0568 | 9 | 0.5175 | 0.0453 | 8.7492 | -0.0069 | 0.0069 | NA |
| line_589 | Abdomen | 11 | 1.0818 | 0.0839 | 7.7539 | 14 | 0.9314 | 0.0480 | 5.1550 | -0.0137 | 0.0137 | MR2 (minor) |
| line_589 | Thorax | 11 | 0.5318 | 0.0357 | 6.7178 | 14 | 0.5370 | 0.0430 | 8.0000 | 0.0005 | 0.0005 | MR2 (minor) |
| line_59 | Abdomen | 5 | 0.9900 | 0.0660 | 6.6621 | 21 | 1.2495 | 0.1063 | 8.5054 | 0.0236 | 0.0236 | NA |
| line_59 | Thorax | 5 | 0.5980 | 0.0327 | 5.4700 | 21 | 0.5700 | 0.0665 | 11.6651 | -0.0025 | 0.0025 | NA |
| line_595 | Thorax | 7 | 0.5800 | 0.0329 | 5.6661 | 13 | 0.6069 | 0.0210 | 3.4551 | 0.0024 | 0.0024 | NA |
| line_595 | Abdomen | 7 | 0.9986 | 0.0790 | 7.9155 | 13 | 1.0662 | 0.0646 | 6.0610 | 0.0061 | 0.0061 | NA |
| line_596 | Thorax | 8 | 0.5725 | 0.0198 | 3.4621 | 7 | 0.5429 | 0.0585 | 10.7788 | -0.0027 | 0.0027 | MR1 (minor) B |
| line_596 | Abdomen | 8 | 1.0288 | 0.0967 | 9.4020 | 7 | 1.0357 | 0.1482 | 14.3085 | 0.0006 | 0.0006 | MR1 (minor) B |
| line_627 | Thorax | 12 | 0.5950 | 0.0321 | 5.3867 | 19 | 0.5322 | 0.0502 | 9.4399 | -0.0057 | 0.0057 | NA |
| line_627 | Abdomen | 12 | 1.1083 | 0.0917 | 8.2768 | 19 | 0.9226 | 0.0896 | 9.7134 | -0.0169 | 0.0169 | NA |
| line_630 | Thorax | 8 | 0.6338 | 0.0431 | 6.7967 | 7 | 0.5257 | 0.0181 | 3.4480 | -0.0098 | 0.0098 | NA |
| line_630 | Abdomen | 8 | 1.1613 | 0.0895 | 7.7083 | 7 | 1.1100 | 0.0622 | 5.6020 | -0.0047 | 0.0047 | NA |
| line_634 | Thorax | 8 | 0.5688 | 0.0327 | 5.7504 | 7 | 0.5257 | 0.0336 | 6.3902 | -0.0039 | 0.0039 | NA |
| line_634 | Abdomen | 8 | 1.1938 | 0.1000 | 8.3762 | 7 | 1.0786 | 0.0736 | 6.8222 | -0.0105 | 0.0105 | NA |
| line_639 | Abdomen | 12 | 0.9958 | 0.0626 | 6.2851 | 17 | 1.0071 | 0.0814 | 8.0806 | 0.0010 | 0.0010 | NA |
| line_639 | Thorax | 12 | 0.5700 | 0.0234 | 4.0974 | 17 | 0.5347 | 0.0464 | 8.6829 | -0.0032 | 0.0032 | NA |
| line_642 | Thorax | 9 | 0.5833 | 0.0485 | 8.3103 | 12 | 0.5508 | 0.0235 | 4.2722 | -0.0030 | 0.0030 | NA |
| line_642 | Abdomen | 9 | 1.2189 | 0.0948 | 7.7772 | 12 | 1.1017 | 0.0800 | 7.2600 | -0.0107 | 0.0107 | NA |
| line_646 | Abdomen | 7 | 1.0971 | 0.0873 | 7.9583 | 14 | 1.0486 | 0.0567 | 5.4059 | -0.0044 | 0.0044 | NA |
| line_646 | Thorax | 7 | 0.6386 | 0.0267 | 4.1853 | 14 | 0.5315 | 0.0391 | 7.3607 | -0.0097 | 0.0097 | NA |
| line_69 | Thorax | 8 | 0.5738 | 0.0453 | 7.9017 | 10 | 0.5225 | 0.0341 | 6.5305 | -0.0047 | 0.0047 | NA |
| line_69 | Abdomen | 8 | 0.9300 | 0.0428 | 4.5980 | 10 | 0.9060 | 0.0617 | 6.8080 | -0.0022 | 0.0022 | NA |
| line_703 | Abdomen | 24 | 1.0779 | 0.0951 | 8.8222 | 11 | 1.0418 | 0.0567 | 5.4437 | -0.0033 | 0.0033 | NA |
| line_703 | Thorax | 25 | 0.5467 | 0.0406 | 7.4289 | 11 | 0.5056 | 0.0343 | 6.7883 | -0.0037 | 0.0037 | NA |
| line_705 | Thorax | 11 | 0.6491 | 0.0367 | 5.6587 | 12 | 0.5267 | 0.0358 | 6.7899 | -0.0111 | 0.0111 | NA |
| line_705 | Abdomen | 11 | 1.1045 | 0.0959 | 8.6778 | 12 | 0.9142 | 0.0692 | 7.5709 | -0.0173 | 0.0173 | NA |
| line_707 | Thorax | 9 | 0.5600 | 0.0335 | 5.9895 | 9 | 0.5056 | 0.0391 | 7.7315 | -0.0049 | 0.0049 | NA |
| line_707 | Abdomen | 9 | 1.2000 | 0.0300 | 2.5000 | 9 | 1.1711 | 0.1045 | 8.9194 | -0.0026 | 0.0026 | NA |
| line_712 | Abdomen | 11 | 1.0291 | 0.1044 | 10.1494 | 13 | 0.9938 | 0.0423 | 4.2598 | -0.0032 | 0.0032 | NA |
| line_712 | Thorax | 11 | 0.5264 | 0.0280 | 5.3244 | 13 | 0.5260 | 0.0232 | 4.4088 | 0.0000 | 0.0000 | NA |
| line_714 | Abdomen | 12 | 0.9750 | 0.0511 | 5.2389 | 9 | 0.8544 | 0.0714 | 8.3603 | -0.0110 | 0.0110 | MR3 (minor) B / MR1 (major) B / MR4 (major) A / MR2 (major) A |
| line_714 | Thorax | 12 | 0.5583 | 0.0279 | 4.9982 | 9 | 0.5025 | 0.0443 | 8.8200 | -0.0051 | 0.0051 | MR3 (minor) B / MR1 (major) B / MR4 (major) A / MR2 (major) A |
| line_716 | Abdomen | 23 | 1.2430 | 0.0962 | 7.7370 | 11 | 0.9682 | 0.0588 | 6.0723 | -0.0250 | 0.0250 | NA |
| line_716 | Thorax | 23 | 0.5923 | 0.0408 | 6.8916 | 11 | 0.5122 | 0.0331 | 6.4586 | -0.0073 | 0.0073 | NA |
| line_721 | Abdomen | 8 | 1.0238 | 0.0370 | 3.6150 | 14 | 1.0214 | 0.0729 | 7.1414 | -0.0002 | 0.0002 | NA |
| line_721 | Thorax | 8 | 0.5838 | 0.0421 | 7.2064 | 14 | 0.5300 | 0.0382 | 7.2094 | -0.0049 | 0.0049 | NA |
| line_727 | Thorax | 10 | 0.6180 | 0.0379 | 6.1403 | 20 | 0.5670 | 0.0266 | 4.6872 | -0.0046 | 0.0046 | NA |
| line_727 | Abdomen | 10 | 1.1810 | 0.0638 | 5.4063 | 20 | 1.0705 | 0.0595 | 5.5594 | -0.0100 | 0.0100 | NA |
| line_73 | Thorax | 12 | 0.5208 | 0.0348 | 6.6741 | 5 | 0.6300 | 0.0274 | 4.3470 | 0.0099 | 0.0099 | MR2 (minor) A |
| line_73 | Abdomen | 12 | 0.9975 | 0.1009 | 10.1169 | 6 | 1.0100 | 0.0495 | 4.9007 | 0.0011 | 0.0011 | MR2 (minor) A |
| line_730 | Thorax | 16 | 0.5894 | 0.0169 | 2.8707 | 20 | 0.5530 | 0.0247 | 4.4720 | -0.0033 | 0.0033 | NA |
| line_730 | Abdomen | 16 | 1.0369 | 0.0930 | 8.9696 | 20 | 1.1045 | 0.0750 | 6.7910 | 0.0061 | 0.0061 | NA |
| line_737 | Abdomen | 8 | 1.1413 | 0.0772 | 6.7620 | 14 | 0.9979 | 0.0446 | 4.4676 | -0.0130 | 0.0130 | NA |
| line_737 | Thorax | 8 | 0.5750 | 0.0441 | 7.6657 | 14 | 0.4993 | 0.0358 | 7.1770 | -0.0069 | 0.0069 | NA |
| line_738 | Thorax | 16 | 0.5214 | 0.0496 | 9.5107 | 21 | 0.5452 | 0.0359 | 6.5776 | 0.0022 | 0.0022 | NA |
| line_738 | Abdomen | 16 | 1.0238 | 0.0658 | 6.4289 | 21 | 1.1005 | 0.0841 | 7.6452 | 0.0070 | 0.0070 | NA |
| line_748 | Abdomen | 12 | 1.1542 | 0.0438 | 3.7939 | 15 | 1.0240 | 0.0688 | 6.7234 | -0.0118 | 0.0118 | MR1 (minor) AMR2 (minor) B |
| line_748 | Thorax | 12 | 0.5258 | 0.0434 | 8.2481 | 15 | 0.5240 | 0.0417 | 7.9606 | -0.0002 | 0.0002 | MR1 (minor) AMR2 (minor) B |
| line_75 | Thorax | 17 | 0.5933 | 0.0616 | 10.3830 | 27 | 0.5680 | 0.0440 | 7.7411 | -0.0023 | 0.0023 | NA |
| line_75 | Abdomen | 17 | 1.1524 | 0.0724 | 6.2842 | 27 | 1.2033 | 0.1272 | 10.5722 | 0.0046 | 0.0046 | NA |
| line_757 | Abdomen | 7 | 0.9240 | 0.0055 | 0.5928 | 9 | 1.0071 | 0.0457 | 4.5397 | 0.0076 | 0.0076 | NA |
| line_757 | Thorax | 7 | 0.5760 | 0.0336 | 5.8360 | 9 | 0.5500 | 0.0630 | 11.4512 | -0.0024 | 0.0024 | NA |
| line_761 | Abdomen | 17 | 1.1465 | 0.0838 | 7.3103 | 11 | 1.0400 | 0.0804 | 7.7283 | -0.0097 | 0.0097 | NA |
| line_761 | Thorax | 17 | 0.5671 | 0.0329 | 5.8076 | 11 | 0.5273 | 0.0429 | 8.1393 | -0.0036 | 0.0036 | NA |
| line_765 | Thorax | 5 | 0.6100 | 0.0374 | 6.1339 | 21 | 0.5414 | 0.0355 | 6.5635 | -0.0062 | 0.0062 | MR3 (major) B / MR1 (major) B / MR4 (minor) A |
| line_765 | Abdomen | 5 | 1.1900 | 0.0640 | 5.3808 | 21 | 0.9575 | 0.0660 | 6.8934 | -0.0211 | 0.0211 | MR3 (major) B / MR1 (major) B / MR4 (minor) A |
| line_774 | Abdomen | 8 | 1.2388 | 0.0615 | 4.9658 | 11 | 1.0882 | 0.0782 | 7.1870 | -0.0137 | 0.0137 | MR4 (major) |
| line_774 | Thorax | 8 | 0.6538 | 0.0400 | 6.1151 | 11 | 0.5620 | 0.0437 | 7.7696 | -0.0083 | 0.0083 | MR4 (major) |
| line_776 | Thorax | 11 | 0.5670 | 0.0337 | 5.9403 | 14 | 0.5523 | 0.0390 | 7.0572 | -0.0013 | 0.0013 | NA |
| line_776 | Abdomen | 11 | 1.0945 | 0.0775 | 7.0812 | 14 | 1.0407 | 0.0748 | 7.1853 | -0.0049 | 0.0049 | NA |
| line_783 | Abdomen | 14 | 1.1179 | 0.0766 | 6.8505 | 7 | 1.1943 | 0.0288 | 2.4102 | 0.0069 | 0.0069 | MR2 (minor) |
| line_783 | Thorax | 14 | 0.5408 | 0.0315 | 5.8214 | 7 | 0.5657 | 0.0310 | 5.4824 | 0.0023 | 0.0023 | MR2 (minor) |
| line_786 | Thorax | 17 | 0.5806 | 0.0300 | 5.1656 | 17 | 0.5693 | 0.0369 | 6.4865 | -0.0010 | 0.0010 | NA |
| line_786 | Abdomen | 17 | 1.0606 | 0.0402 | 3.7931 | 16 | 1.0494 | 0.0861 | 8.2010 | -0.0010 | 0.0010 | NA |
| line_787 | Abdomen | 22 | 1.0982 | 0.0906 | 8.2536 | 15 | 0.9707 | 0.0631 | 6.4978 | -0.0116 | 0.0116 | MR3 (major) B |
| line_787 | Thorax | 22 | 0.5762 | 0.0439 | 7.6142 | 15 | 0.5108 | 0.0348 | 6.8038 | -0.0059 | 0.0059 | MR3 (major) B |
| line_790 | Abdomen | 12 | 1.0992 | 0.0899 | 8.1784 | 12 | 0.9533 | 0.0675 | 7.0767 | -0.0133 | 0.0133 | MR2 (minor) |
| line_790 | Thorax | 12 | 0.5183 | 0.0369 | 7.1163 | 12 | 0.5575 | 0.0445 | 7.9898 | 0.0036 | 0.0036 | MR2 (minor) |
| line_796 | Thorax | 18 | 0.5594 | 0.0383 | 6.8402 | 22 | 0.5657 | 0.0403 | 7.1273 | 0.0006 | 0.0006 | MR2 (minor) |
| line_796 | Abdomen | 18 | 1.2056 | 0.0726 | 6.0235 | 21 | 1.0924 | 0.0847 | 7.7510 | -0.0103 | 0.0103 | MR2 (minor) |
| line_799 | Thorax | 5 | 0.5840 | 0.0207 | 3.5508 | 7 | 0.5614 | 0.0324 | 5.7651 | -0.0021 | 0.0021 | NA |
| line_799 | Abdomen | 5 | 1.2080 | 0.0669 | 5.5346 | 7 | 1.0986 | 0.0776 | 7.0593 | -0.0099 | 0.0099 | NA |
| line_801 | Thorax | 11 | 0.6100 | 0.0298 | 4.8876 | 13 | 0.5492 | 0.0287 | 5.2351 | -0.0055 | 0.0055 | NA |
| line_801 | Abdomen | 11 | 1.1718 | 0.1203 | 10.2676 | 13 | 0.9515 | 0.0628 | 6.6028 | -0.0200 | 0.0200 | NA |
| line_802 | Abdomen | 22 | 1.2809 | 0.0985 | 7.6867 | 24 | 1.1342 | 0.0636 | 5.6061 | -0.0133 | 0.0133 | NA |
| line_802 | Thorax | 22 | 0.6332 | 0.0380 | 5.9968 | 24 | 0.5670 | 0.0397 | 7.0036 | -0.0060 | 0.0060 | NA |
| line_804 | Thorax | 11 | 0.5756 | 0.0410 | 7.1167 | 13 | 0.4775 | 0.0347 | 7.2615 | -0.0089 | 0.0089 | NA |
| line_804 | Abdomen | 11 | 1.1982 | 0.1227 | 10.2409 | 13 | 1.0385 | 0.0972 | 9.3598 | -0.0145 | 0.0145 | NA |
| line_805 | Abdomen | 9 | 1.1100 | 0.0811 | 7.3051 | 16 | 1.0638 | 0.0627 | 5.8945 | -0.0042 | 0.0042 | NA |
| line_805 | Thorax | 9 | 0.5944 | 0.0288 | 4.8400 | 16 | 0.5445 | 0.0602 | 11.0600 | -0.0045 | 0.0045 | NA |
| line_808 | Thorax | 10 | 0.6170 | 0.0432 | 7.0045 | 11 | 0.5391 | 0.0367 | 6.8133 | -0.0071 | 0.0071 | NA |
| line_808 | Abdomen | 10 | 1.0200 | 0.0583 | 5.7166 | 11 | 0.9391 | 0.0682 | 7.2607 | -0.0074 | 0.0074 | NA |
| line_809 | Abdomen | NA | NA | NA | NA | 8 | 1.1525 | 0.0978 | 8.4857 | NA | NA | NA |
| line_809 | Thorax | NA | NA | NA | NA | 9 | 0.5613 | 0.0627 | 11.1651 | NA | NA | NA |
| line_810 | Thorax | 9 | 0.5822 | 0.0205 | 3.5176 | 12 | 0.5917 | 0.0295 | 4.9843 | 0.0009 | 0.0009 | NA |
| line_810 | Abdomen | 9 | 1.0622 | 0.0458 | 4.3084 | 11 | 0.9900 | 0.0523 | 5.2874 | -0.0066 | 0.0066 | NA |
| line_818 | Thorax | 9 | 0.5956 | 0.0498 | 8.3581 | 10 | 0.5467 | 0.0245 | 4.4808 | -0.0044 | 0.0044 | MR1 (major) AMR2 (major) B |
| line_818 | Abdomen | 9 | 0.9656 | 0.0893 | 9.2505 | 10 | 0.9800 | 0.0615 | 6.2718 | 0.0013 | 0.0013 | MR1 (major) AMR2 (major) B |
| line_819 | Abdomen | 7 | 1.0014 | 0.0291 | 2.9072 | 31 | 0.9981 | 0.0537 | 5.3796 | -0.0003 | 0.0003 | NA |
| line_819 | Thorax | 7 | 0.6486 | 0.0363 | 5.5897 | 31 | 0.5582 | 0.0389 | 6.9647 | -0.0082 | 0.0082 | NA |
| line_820 | Abdomen | 18 | 1.1550 | 0.0857 | 7.4197 | 12 | 0.9500 | 0.0386 | 4.0645 | -0.0186 | 0.0186 | NA |
| line_820 | Thorax | 19 | 0.5994 | 0.0368 | 6.1431 | 12 | 0.5767 | 0.0464 | 8.0435 | -0.0021 | 0.0021 | NA |
| line_821 | Abdomen | 8 | 1.0663 | 0.0362 | 3.3977 | 9 | 1.1267 | 0.0700 | 6.2130 | 0.0055 | 0.0055 | NA |
| line_821 | Thorax | 8 | 0.5750 | 0.0382 | 6.6387 | 9 | 0.5567 | 0.0555 | 9.9616 | -0.0017 | 0.0017 | NA |
| line_822 | Abdomen | 10 | 1.2489 | 0.0810 | 6.4858 | 34 | 1.0747 | 0.0543 | 5.0512 | -0.0158 | 0.0158 | MR4 (major) B |
| line_822 | Thorax | 10 | 0.6178 | 0.0444 | 7.1836 | 34 | 0.5510 | 0.0345 | 6.2585 | -0.0061 | 0.0061 | MR4 (major) B |
| line_83 | Abdomen | 10 | 1.2380 | 0.0447 | 3.6084 | NA | NA | NA | NA | NA | NA | MR3 (minor) A / MR1 (minor) B |
| line_83 | Thorax | 10 | 0.6011 | 0.0420 | 6.9813 | NA | NA | NA | NA | NA | NA | MR3 (minor) A / MR1 (minor) B |
| line_832 | Thorax | 11 | 0.5445 | 0.0333 | 6.1107 | 6 | 0.6033 | 0.0320 | 5.3108 | 0.0053 | 0.0053 | MR3 (major) A / MR1 (minor) B / MR4 (minor) B / MR2 (major) B |
| line_832 | Abdomen | 10 | 0.8870 | 0.0690 | 7.7755 | 6 | 1.1717 | 0.0627 | 5.3550 | 0.0259 | 0.0259 | MR3 (major) A / MR1 (minor) B / MR4 (minor) B / MR2 (major) B |
| line_837 | Abdomen | 19 | 1.0774 | 0.0867 | 8.0493 | 7 | 1.0929 | 0.0565 | 5.1685 | 0.0014 | 0.0014 | NA |
| line_837 | Thorax | 19 | 0.5535 | 0.0394 | 7.1109 | 7 | 0.5329 | 0.0373 | 6.9980 | -0.0019 | 0.0019 | NA |
| line_843 | Thorax | 13 | 0.6025 | 0.0277 | 4.5934 | 11 | 0.6164 | 0.0250 | 4.0575 | 0.0013 | 0.0013 | NA |
| line_843 | Abdomen | 13 | 0.9492 | 0.0609 | 6.4148 | 11 | 1.0509 | 0.0539 | 5.1323 | 0.0092 | 0.0092 | NA |
| line_849 | Abdomen | 6 | 0.9433 | 0.1669 | 17.6961 | 5 | 1.0500 | 0.0742 | 7.0630 | 0.0097 | 0.0097 | NA |
| line_849 | Thorax | 6 | 0.6300 | 0.0089 | 1.4197 | 5 | 0.5800 | 0.0447 | 7.7106 | -0.0045 | 0.0045 | NA |
| line_85 | Thorax | 11 | 0.5918 | 0.0433 | 7.3193 | 8 | 0.5800 | 0.0680 | 11.7299 | -0.0011 | 0.0011 | NA |
| line_85 | Abdomen | 11 | 1.1573 | 0.0748 | 6.4674 | 8 | 1.2000 | 0.1437 | 11.9772 | 0.0039 | 0.0039 | NA |
| line_850 | Thorax | NA | NA | NA | NA | 12 | 0.5400 | 0.0358 | 6.6254 | NA | NA | NA |
| line_850 | Abdomen | NA | NA | NA | NA | 12 | 1.0325 | 0.0778 | 7.5376 | NA | NA | NA |
| line_852 | Thorax | 12 | 0.5620 | 0.0368 | 6.5405 | 37 | 0.5348 | 0.0456 | 8.5197 | -0.0025 | 0.0025 | MR3 (major) B / MR1 (major) A / MR4 (minor) A / MR2 (major) B |
| line_852 | Abdomen | 12 | 0.9936 | 0.0923 | 9.2925 | 37 | 0.9792 | 0.1476 | 15.0722 | -0.0013 | 0.0013 | MR3 (major) B / MR1 (major) A / MR4 (minor) A / MR2 (major) B |
| line_853 | Abdomen | 15 | 1.1353 | 0.0755 | 6.6488 | 12 | 1.0175 | 0.0418 | 4.1087 | -0.0107 | 0.0107 | NA |
| line_853 | Thorax | 15 | 0.6207 | 0.0273 | 4.3991 | 12 | 0.5736 | 0.0383 | 6.6734 | -0.0043 | 0.0043 | NA |
| line_855 | Abdomen | 17 | 1.1882 | 0.0745 | 6.2713 | 20 | 1.0340 | 0.0656 | 6.3488 | -0.0140 | 0.0140 | NA |
| line_855 | Thorax | 17 | 0.5869 | 0.0353 | 6.0233 | 20 | 0.5190 | 0.0491 | 9.4579 | -0.0062 | 0.0062 | NA |
| line_857 | Thorax | 17 | 0.6173 | 0.0361 | 5.8555 | 26 | 0.5440 | 0.0447 | 8.2208 | -0.0067 | 0.0067 | NA |
| line_857 | Abdomen | 16 | 1.1294 | 0.0745 | 6.5982 | 26 | 1.0038 | 0.0898 | 8.9503 | -0.0114 | 0.0114 | NA |
| line_859 | Thorax | 11 | 0.5664 | 0.0314 | 5.5427 | 8 | 0.5488 | 0.0309 | 5.6326 | -0.0016 | 0.0016 | NA |
| line_859 | Abdomen | 11 | 1.1991 | 0.0769 | 6.4107 | 8 | 1.1663 | 0.0826 | 7.0846 | -0.0030 | 0.0030 | NA |
| line_861 | Thorax | 12 | 0.5425 | 0.0273 | 5.0405 | 14 | 0.5108 | 0.0299 | 5.8441 | -0.0029 | 0.0029 | no |
| line_861 | Abdomen | 12 | 1.1800 | 0.0484 | 4.1042 | 14 | 1.1507 | 0.0891 | 7.7463 | -0.0027 | 0.0027 | no |
| line_879 | Thorax | 14 | 0.6617 | 0.0147 | 2.2246 | 6 | NA | NA | NA | NA | NA | MR1 (major) |
| line_879 | Abdomen | 14 | 1.0267 | 0.0418 | 4.0708 | 6 | NA | NA | NA | NA | NA | MR1 (major) |
| line_88 | Thorax | 16 | 0.6420 | 0.0329 | 5.1294 | 20 | 0.5047 | 0.0431 | 8.5438 | -0.0125 | 0.0125 | NA |
| line_88 | Abdomen | 16 | 1.2360 | 0.0675 | 5.4634 | 20 | 1.1115 | 0.1408 | 12.6707 | -0.0113 | 0.0113 | NA |
| line_882 | Abdomen | 7 | 1.0786 | 0.0372 | 3.4454 | 10 | 1.0011 | 0.0033 | 0.3330 | -0.0071 | 0.0071 | NA |
| line_882 | Thorax | 7 | 0.5843 | 0.0053 | 0.9148 | 10 | 0.5300 | 0.0050 | 0.9434 | -0.0049 | 0.0049 | NA |
| line_884 | Thorax | 12 | 0.5983 | 0.0473 | 7.8983 | 17 | 0.5813 | 0.0368 | 6.3326 | -0.0015 | 0.0015 | NA |
| line_884 | Abdomen | 12 | 0.9783 | 0.0518 | 5.2993 | 17 | 0.9125 | 0.0695 | 7.6189 | -0.0060 | 0.0060 | NA |
| line_887 | Thorax | 11 | 0.6360 | 0.0196 | 3.0740 | 9 | 0.5111 | 0.0379 | 7.4144 | -0.0114 | 0.0114 | NA |
| line_887 | Abdomen | 11 | 1.0218 | 0.0739 | 7.2290 | 9 | 0.9278 | 0.1491 | 16.0756 | -0.0085 | 0.0085 | NA |
| line_890 | Abdomen | 22 | 1.1047 | 0.0835 | 7.5609 | 10 | 1.0500 | 0.0726 | 6.9116 | -0.0050 | 0.0050 | NA |
| line_890 | Thorax | 22 | 0.5513 | 0.0488 | 8.8557 | 10 | 0.5720 | 0.0426 | 7.4537 | 0.0019 | 0.0019 | NA |
| line_892 | Thorax | 15 | 0.6186 | 0.0470 | 7.6053 | 16 | 0.5593 | 0.0456 | 8.1501 | -0.0054 | 0.0054 | MR4 (minor) B / MR2 (minor) B |
| line_892 | Abdomen | 15 | 1.1580 | 0.1007 | 8.6983 | 16 | 1.1553 | 0.2754 | 23.8389 | -0.0002 | 0.0002 | MR4 (minor) B / MR2 (minor) B |
| line_894 | Abdomen | 7 | 1.0414 | 0.0937 | 8.9979 | NA | NA | NA | NA | NA | NA | NA |
| line_894 | Thorax | 7 | 0.5843 | 0.0538 | 9.2091 | NA | NA | NA | NA | NA | NA | NA |
| line_897 | Thorax | 10 | 0.5338 | 0.0457 | 8.5527 | 6 | 0.5000 | 0.0310 | 6.1968 | -0.0031 | 0.0031 | no |
| line_897 | Abdomen | 10 | 1.0250 | 0.0782 | 7.6302 | 6 | 0.9183 | 0.0643 | 7.0037 | -0.0097 | 0.0097 | no |
| line_900 | Thorax | 11 | 0.5818 | 0.0440 | 7.5632 | 12 | 0.5558 | 0.0309 | 5.5562 | -0.0024 | 0.0024 | NA |
| line_900 | Abdomen | 11 | 0.9236 | 0.0879 | 9.5161 | 12 | 1.0825 | 0.1608 | 14.8546 | 0.0144 | 0.0144 | NA |
| line_907 | Abdomen | 10 | 1.0720 | 0.0553 | 5.1621 | 8 | 1.0213 | 0.0488 | 4.7810 | -0.0046 | 0.0046 | NA |
| line_907 | Thorax | 10 | 0.6380 | 0.0312 | 4.8900 | 8 | 0.5600 | 0.0393 | 7.0141 | -0.0071 | 0.0071 | NA |
| line_908 | Thorax | 10 | 0.6070 | 0.0437 | 7.2041 | 10 | 0.5022 | 0.0360 | 7.1638 | -0.0095 | 0.0095 | NA |
| line_908 | Abdomen | 10 | 1.0350 | 0.0654 | 6.3152 | 10 | 0.9490 | 0.0711 | 7.4915 | -0.0078 | 0.0078 | NA |
| line_91 | Thorax | 11 | 0.5380 | 0.0410 | 7.6286 | 11 | 0.5555 | 0.0298 | 5.3627 | 0.0016 | 0.0016 | NA |
| line_91 | Abdomen | 11 | 1.1491 | 0.0938 | 8.1586 | 11 | 1.1127 | 0.0605 | 5.4383 | -0.0033 | 0.0033 | NA |
| line_911 | Thorax | 8 | 0.5938 | 0.0177 | 2.9773 | 26 | 0.5170 | 0.0347 | 6.7120 | -0.0070 | 0.0070 | MR4 (major) A / MR2 (major) A |
| line_911 | Abdomen | 8 | 1.0129 | 0.0496 | 4.8940 | 26 | 0.8488 | 0.0703 | 8.2772 | -0.0149 | 0.0149 | MR4 (major) A / MR2 (major) A |
| line_913 | Thorax | 12 | 0.5173 | 0.0358 | 6.9214 | 10 | 0.5644 | 0.0207 | 3.6643 | 0.0043 | 0.0043 | NA |
| line_913 | Abdomen | 12 | 1.0325 | 0.0458 | 4.4311 | 10 | 1.3760 | 0.1133 | 8.2307 | 0.0312 | 0.0312 | NA |
| line_93 | Thorax | 12 | 0.6427 | 0.0438 | 6.8207 | 10 | 0.6020 | 0.0358 | 5.9533 | -0.0037 | 0.0037 | MR3 (minor) A / MR4 (major) A |
| line_93 | Abdomen | 12 | 1.1742 | 0.1568 | 13.3503 | 10 | 1.1770 | 0.1018 | 8.6464 | 0.0003 | 0.0003 | MR3 (minor) A / MR4 (major) A |
