# Supplementary material for: Genetic basis of thermal plasticity variation in Drosophila melanogaster body size
Source: PLoS Genet. 2018 Sep 26;14(9):e1007686. doi: 10.1371/journal.pgen.1007686 (PMC6175520; doi:10.1371/journal.pgen.1007686)
Supplement: S3 Table — (DOCX) [file pgen.1007686.s010.docx]

| **S3 Table. GWAS results for variation in size plasticity.** Nominally significant SNPs (p-value < 10e-5) from GWAS for the raw slope of the reaction norms (Value=Raw) and absolute slope of the reaction norms (Value=Absolute) per body part. This table includes the overlap between our candidate QTLs and genes that showed changes in populations evolving under cold and hot fluctuating thermal regimes in Tobler et al. (2014). The genomic position (given by annotation with Genome Releases v.5. and v.6.), type of SNP/Indel, potential impact, associated gene name (Flybase Gene ID) and putative consequence are also shown. | | | | | | | | | |
| --- | --- | --- | --- | --- | --- | --- | --- | --- | --- |
| **Genomic position (v.6.)** | **Genomic position (v.5.)** | **SNP/Indel** | **Value** | **P value** | **Major allele** | **Minor allele** | **Gene symbol** | **Affected region** | **Thermal fluctuation** |
| 2R:4854197 | 2R:741702 | SNP | Raw | 6.13E-06 | G | A | - | intergenic variant | NA |
| 3L:10923119 | 3L:10916219 | SNP | Raw | 7.41E-07 | G | C | - | intergenic variant | NA |
| 3L:12321780 | 3L:12314880 | DEL | Absolute | 6.35E-06 | TAGTGCAGTTC | T | - | intergenic variant | NA |
| 3L:781082 | 3L:781082 | SNP | Absolute | 9.71E-06 | A | G | - | intergenic variant | NA |
| 3R:13896385 | 3R:9722107 | SNP | Absolute | 2.21E-06 | A | G | - | intergenic variant | NA |
| 3R:13896385 | 3R:9722107 | SNP | Absolute | 2.21E-06 | A | G | - | intergenic variant | NA |
| 3R:24978474 | 3R:20804196 | SNP | Absolute | 6.77E-06 | C | G | - | intergenic variant | NA |
| 3R:24978474 | 3R:20804196 | SNP | Absolute | 6.77E-06 | C | G | - | intergenic variant | NA |
| 3R:25145642 | 3R:20971364 | SNP | Absolute | 8.31E-06 | T | C | - | intergenic variant | NA |
| 3R:25145642 | 3R:20971364 | SNP | Absolute | 8.31E-06 | T | C | - | intergenic variant | NA |
| X:14564271 | X:14458304 | SNP | Absolute | 7.34E-06 | T | C | - | intergenic variant | NA |
| X:14567463 | X:14461496 | SNP | Absolute | 8.39E-06 | C | G | - | intergenic variant | NA |
| X:14567474 | X:14461507 | SNP | Absolute | 3.09E-06 | C | A | - | intergenic variant | NA |
| X:6391641 | X:6285674 | DEL | Absolute | 4.00E-06 | TATCATTAGAATTGATTGATCCT | T | - | intergenic variant | NA |
| X:6391641 | X:6285674 | DEL | Absolute | 4.00E-06 | TATCATTAGAATTGATTGATCCT | T | - | intergenic variant | NA |
| 2R:7983239 | 2R:3870744 | SNP | Raw | 6.10E-06 | C | T | ACC | intron variant | NA |
| 2R:7983798 | 2R:3871303 | SNP | Raw | 5.31E-06 | G | T | ACC | 5 prime UTR variant | NA |
| 2R:7983798 | 2R:3871303 | SNP | Raw | 5.31E-06 | G | T | ACC | intron variant | NA |
| X:15362032 | X:15256065 | SNP | Absolute | 3.65E-06 | G | A | acj6 | intron variant | NA |
| X:15362032 | X:15256065 | SNP | Absolute | 3.65E-06 | G | A | acj6 | intron variant | NA |
| X:15362032 | X:15256065 | SNP | Raw | 7.11E-06 | G | A | acj6 | intron variant | NA |
| 2R:21396062 | 2R:17283567 | SNP | Absolute | 5.34E-06 | T | A | Acox57D-d | intergenic variant | NA |
| 3L:2245066 | 3L:2245066 | SNP | Absolute | 9.43E-06 | A | G | ACXD | intergenic variant | NA |
| 3R:13712326 | 3R:9538048 | SNP | Absolute | 1.77E-06 | A | G | Adgf-C | intergenic variant | NA |
| 3L:16655829 | 3L:16648929 | SNP | Absolute | 6.45E-07 | A | G | Baldspot | intron variant | NA |
| 3L:16655829 | 3L:16648929 | SNP | Absolute | 6.45E-07 | A | G | Baldspot | intron variant | NA |
| 3L:6992697 | 3L:6985797 | SNP | Raw | 8.35E-07 | T | C | bin | synonymous variant | NA |
| 3L:6992724 | 3L:6985824 | SNP | Raw | 2.12E-06 | T | C | bin | synonymous variant | NA |
| 2L:17961008 | 2L:17961008 | SNP | Raw | 3.67E-06 | T | A | btv | intergenic variant | hot & cold |
| 2L:17969392 | 2L:17969392 | SNP | Raw | 6.31E-06 | A | G | btv | intron variant | hot & cold |
| 2L:16310742 | 2L:16310742 | SNP | Absolute | 5.08E-06 | C | T | c(2)M | intergenic variant | NA |
| 2L:16310742 | 2L:16310742 | SNP | Absolute | 5.08E-06 | C | T | cact | intergenic variant | NA |
| 2L:16818587 | 2L:16818587 | SNP | Absolute | 5.13E-06 | C | T | Cas | intergenic variant | cold |
| 2R:21072501 | 2R:16960006 | SNP | Absolute | 9.97E-06 | T | G | CG10527 | intron variant | NA |
| 3L:12951673 | 3L:12944773 | SNP | Absolute | 2.81E-06 | T | C | CG10752 | intergenic variant | NA |
| 2R:7180821 | 2R:3068326 | SNP | Raw | 6.38E-06 | C | G | CG11060 | intergenic variant | NA |
| X:11108613 | X:11002646 | INS | Absolute | 3.80E-06 | G | GTAC | CG11122 | intergenic variant | NA |
| X:11108613 | X:11002646 | INS | Absolute | 3.80E-06 | G | GTAC | CG11122 | intergenic variant | NA |
| 3R:5826778 | 3R:1652500 | SNP | Absolute | 4.87E-06 | C | T | CG1208 | intergenic variant | NA |
| 3R:5826778 | 3R:1652500 | SNP | Absolute | 4.87E-06 | C | T | CG1208 | intergenic variant | NA |
| 2R:10353132 | 2R:6240637 | SNP | Raw | 3.66E-06 | C | T | CG12902 | intergenic variant | NA |
| 2R:10353132 | 2R:6240637 | SNP | Raw | 3.66E-06 | C | T | CG12907 | intergenic variant | NA |
| 3R:9328010 | 3R:5153732 | SNP | Absolute | 9.19E-06 | A | G | CG12951 | intergenic variant | cold |
| X:17433111 | X:17327144 | SNP | Absolute | 6.48E-06 | C | T | CG12994 | intron variant | NA |
| 3L:20727281 | 3L:20720381 | SNP | Absolute | 9.17E-06 | A | G | CG13252 | intergenic variant | cold |
| 2L:16815764 | 2L:16815764 | SNP | Absolute | 8.19E-06 | G | A | CG13272 | missense variant | NA |
| 2L:16818587 | 2L:16818587 | SNP | Absolute | 5.13E-06 | C | T | CG13272 | intergenic variant | NA |
| 2L:16815764 | 2L:16815764 | SNP | Absolute | 8.19E-06 | G | A | CG13280 | intron variant | NA |
| 2L:16818587 | 2L:16818587 | SNP | Absolute | 5.13E-06 | C | T | CG13280 | intron variant | NA |
| 2R:20517687 | 2R:16405192 | SNP | Absolute | 9.69E-06 | A | G | CG13426 | intergenic variant | NA |
| 3R:26454823 | 3R:22280545 | SNP | Raw | 5.43E-06 | C | G | CG14237 | intergenic variant | NA |
| 3R:26454823 | 3R:22280545 | SNP | Raw | 5.43E-06 | C | G | CG14238 | intergenic variant | hot |
| 3R:28955902 | 3R:24781624 | SNP | Raw | 2.55E-06 | C | A | CG14521 | intron variant | NA |
| 3R:28817976 | 3R:24643698 | SNP | Raw | 5.71E-06 | C | T | CG1523 | synonymous variant | NA |
| X:8276762 | X:8170795 | SNP | Absolute | 9.79E-06 | A | C | CG15343 | intergenic variant | NA |
| X:8276770 | X:8170803 | SNP | Absolute | 9.78E-08 | A | T | CG15343 | intergenic variant | NA |
| X:12460715 | X:12354748 | SNP | Absolute | 9.32E-07 | A | G | CG15731 | intergenic variant | NA |
| X:12460715 | X:12354748 | SNP | Absolute | 9.32E-07 | A | G | CG15731 | intergenic variant | NA |
| X:12460715 | X:12354748 | SNP | Raw | 8.95E-06 | A | G | CG15731 | intergenic variant | NA |
| X:12460721 | X:12354754 | SNP | Absolute | 4.94E-06 | G | A | CG15731 | intergenic variant | NA |
| X:12460721 | X:12354754 | SNP | Absolute | 4.94E-06 | G | A | CG15731 | intergenic variant | NA |
| X:12460725 | X:12354758 | SNP | Absolute | 2.54E-06 | C | T | CG15731 | intergenic variant | NA |
| X:12460725 | X:12354758 | SNP | Absolute | 2.54E-06 | C | T | CG15731 | intergenic variant | NA |
| X:12460908 | X:12354941 | SNP | Absolute | 1.81E-06 | C | G | CG15731 | intergenic variant | NA |
| X:12460908 | X:12354941 | SNP | Absolute | 1.81E-06 | C | G | CG15731 | intergenic variant | NA |
| X:8276762 | X:8170795 | SNP | Absolute | 9.79E-06 | A | C | CG1632 | intergenic variant | NA |
| X:8276770 | X:8170803 | SNP | Absolute | 9.78E-08 | A | T | CG1632 | intergenic variant | NA |
| X:8276762 | X:8170795 | SNP | Absolute | 9.79E-06 | A | C | CG1636 | intergenic variant | NA |
| X:8276770 | X:8170803 | SNP | Absolute | 9.78E-08 | A | T | CG1636 | intergenic variant | NA |
| 3R:28817976 | 3R:24643698 | SNP | Raw | 5.71E-06 | C | T | CG1646 | intergenic variant | NA |
| 3R:28817976 | 3R:24643698 | SNP | Raw | 5.71E-06 | C | T | CG1647 | intergenic variant | NA |
| 3R:9328010 | 3R:5153732 | SNP | Absolute | 9.19E-06 | A | G | CG16749 | intergenic variant | NA |
| 3R:23060285 | 3R:18886007 | SNP | Absolute | 2.69E-06 | T | A | CG17111 | intergenic variant | cold |
| 3R:23060285 | 3R:18886007 | SNP | Absolute | 2.69E-06 | T | A | CG17111 | intergenic variant | cold |
| 3R:23060285 | 3R:18886007 | SNP | Absolute | 2.69E-06 | T | A | CG17119 | intergenic variant | cold |
| 3R:23060285 | 3R:18886007 | SNP | Absolute | 2.69E-06 | T | A | CG17119 | intergenic variant | cold |
| 2R:12302662 | 2R:8190167 | INS | Absolute | 7.60E-08 | G | GGGATG | CG17739 | intergenic variant | NA |
| 2R:12302662 | 2R:8190167 | INS | Absolute | 7.60E-08 | G | GGGATG | CG17739 | intergenic variant | NA |
| 2R:12302662 | 2R:8190167 | INS | Raw | 1.32E-06 | G | GGGATG | CG17739 | intergenic variant | NA |
| 3L:11947080 | 3L:11940180 | SNP | Absolute | 1.91E-06 | C | T | CG17826 | intergenic variant | NA |
| 3R:5826778 | 3R:1652500 | SNP | Absolute | 4.87E-06 | C | T | CG2023 | intergenic variant | NA |
| 3R:5826778 | 3R:1652500 | SNP | Absolute | 4.87E-06 | C | T | CG2023 | intergenic variant | NA |
| 3R:5769927 | 3R:1595649 | SNP | Absolute | 3.92E-06 | C | A | CG2082 | intron variant | NA |
| 3R:5769927 | 3R:1595649 | SNP | Absolute | 3.92E-06 | C | A | CG2082 | intron variant | NA |
| 2R:15716133 | 2R:11603638 | SNP | Raw | 4.27E-06 | C | T | CG30083 | intergenic variant | NA |
| 2R:15716344 | 2R:11603849 | SNP | Raw | 7.43E-06 | G | C | CG30083 | intergenic variant | NA |
| 2R:16322619 | 2R:12210124 | SNP | Absolute | 6.37E-06 | A | C | CG30096 | intergenic variant | NA |
| 2R:16322619 | 2R:12210124 | SNP | Absolute | 6.37E-06 | A | C | CG30096 | intergenic variant | NA |
| 2R:16322619 | 2R:12210124 | SNP | Raw | 8.92E-06 | A | C | CG30096 | intergenic variant | NA |
| 2R:7180821 | 2R:3068326 | SNP | Raw | 6.38E-06 | C | G | CG30384 | intergenic variant | NA |
| 2R:7180821 | 2R:3068326 | SNP | Raw | 6.38E-06 | C | G | CG30385 | intergenic variant | NA |
| 3R:13712326 | 3R:9538048 | SNP | Absolute | 1.77E-06 | A | G | CG31469 | intergenic variant | hot & cold |
| 3R:19190327 | 3R:15016049 | SNP | Absolute | 4.88E-07 | A | G | CG31475 | missense variant | hot & cold |
| 3R:19190327 | 3R:15016049 | SNP | Absolute | 4.88E-07 | A | G | CG31475 | missense variant | hot & cold |
| 2L:5647089 | 2L:5647089 | SNP | Raw | 3.71E-06 | G | A | CG31646 | intron variant | NA |
| 2L:1595899 | 2L:1595899 | SNP | Raw | 4.45E-07 | C | G | CG31935 | synonymous variant | NA |
| 3L:2245066 | 3L:2245066 | SNP | Absolute | 9.43E-06 | A | G | CG32301 | splice region variant/intron variant | NA |
| 3L:2245066 | 3L:2245066 | SNP | Absolute | 9.43E-06 | A | G | CG32305 | intergenic variant | NA |
| X:16724373 | X:16618406 | INS | Absolute | 6.83E-06 | A | AA | CG32572 | intron variant | NA |
| X:12778133 | X:12672166 | SNP | Raw | 6.95E-06 | T | G | CG32647 | intron variant | NA |
| X:12778134 | X:12672167 | SNP | Raw | 1.20E-06 | A | C | CG32647 | intron variant | NA |
| X:12778138 | X:12672171 | SNP | Raw | 7.15E-06 | T | A | CG32647 | intron variant | NA |
| X:12778151 | X:12672184 | SNP | Raw | 5.98E-07 | A | T | CG32647 | intron variant | NA |
| X:12778242 | X:12672275 | SNP | Raw | 1.76E-06 | C | T | CG32647 | intron variant | NA |
| 2R:7180821 | 2R:3068326 | SNP | Raw | 6.38E-06 | C | G | CG33140 | intergenic variant | NA |
| 2R:12526102 | 2R:8413607 | SNP | Absolute | 2.42E-07 | C | T | CG33632 | intergenic variant | NA |
| 3R:26325774 | 3R:22151496 | SNP | Absolute | 2.09E-07 | T | G | CG33970 | intron variant | cold |
| 3R:26325774 | 3R:22151496 | SNP | Raw | 8.64E-07 | T | G | CG33970 | intron variant | cold |
| X:16724373 | X:16618406 | INS | Absolute | 6.83E-06 | A | AA | CG34325 | intergenic variant | NA |
| 3R:13712326 | 3R:9538048 | SNP | Absolute | 1.77E-06 | A | G | CG34383 | intergenic variant | hot & cold |
| X:11108613 | X:11002646 | INS | Absolute | 3.80E-06 | G | GTAC | CG42249 | intergenic variant | NA |
| X:11108613 | X:11002646 | INS | Absolute | 3.80E-06 | G | GTAC | CG42249 | intergenic variant | NA |
| X:12460715 | X:12354748 | SNP | Absolute | 9.32E-07 | A | G | CG42258 | intergenic variant | NA |
| X:12460715 | X:12354748 | SNP | Absolute | 9.32E-07 | A | G | CG42258 | intergenic variant | NA |
| X:12460715 | X:12354748 | SNP | Raw | 8.95E-06 | A | G | CG42258 | intergenic variant | NA |
| X:12460721 | X:12354754 | SNP | Absolute | 4.94E-06 | G | A | CG42258 | intergenic variant | NA |
| X:12460721 | X:12354754 | SNP | Absolute | 4.94E-06 | G | A | CG42258 | intergenic variant | NA |
| X:12460725 | X:12354758 | SNP | Absolute | 2.54E-06 | C | T | CG42258 | intergenic variant | NA |
| X:12460725 | X:12354758 | SNP | Absolute | 2.54E-06 | C | T | CG42258 | intergenic variant | NA |
| X:12460908 | X:12354941 | SNP | Absolute | 1.81E-06 | C | G | CG42258 | intergenic variant | NA |
| X:12460908 | X:12354941 | SNP | Absolute | 1.81E-06 | C | G | CG42258 | intergenic variant | NA |
| 3L:7461922 | 3L:7455022 | SNP | Absolute | 5.29E-06 | C | T | CG42660 | intergenic variant | NA |
| 3L:7461922 | 3L:7455022 | SNP | Absolute | 5.29E-06 | C | T | CG42660 | intergenic variant | NA |
| 3L:7461922 | 3L:7455022 | SNP | Absolute | 5.29E-06 | C | T | CG42661 | intergenic variant | NA |
| 3L:7461922 | 3L:7455022 | SNP | Absolute | 5.29E-06 | C | T | CG42661 | intergenic variant | NA |
| 2R:12302662 | 2R:8190167 | INS | Absolute | 7.60E-08 | G | GGGATG | CG42700 | intergenic variant | NA |
| 2R:12302662 | 2R:8190167 | INS | Absolute | 7.60E-08 | G | GGGATG | CG42700 | intergenic variant | NA |
| 2R:12302662 | 2R:8190167 | INS | Raw | 1.32E-06 | G | GGGATG | CG42700 | intergenic variant | NA |
| 2R:10353132 | 2R:6240637 | SNP | Raw | 3.66E-06 | C | T | CG42732 | intron variant | cold |
| 2R:10353132 | 2R:6240637 | SNP | Raw | 3.66E-06 | C | T | CG42733 | intergenic variant | NA |
| 2R:16322619 | 2R:12210124 | SNP | Absolute | 6.37E-06 | A | C | CG4282 | synonymous variant | NA |
| 2R:16322619 | 2R:12210124 | SNP | Absolute | 6.37E-06 | A | C | CG4282 | synonymous variant | NA |
| 2R:16322619 | 2R:12210124 | SNP | Raw | 8.92E-06 | A | C | CG4282 | synonymous variant | NA |
| 3L:15647417 | 3L:15640517 | SNP | Raw | 9.06E-06 | C | T | CG43083 | intergenic variant | NA |
| 3L:15647417 | 3L:15640517 | SNP | Raw | 9.06E-06 | C | T | CG43084 | intergenic variant | NA |
| 3R:26454823 | 3R:22280545 | SNP | Raw | 5.43E-06 | C | G | CG43117 | missense variant | NA |
| 3L:8495741 | 3L:8488841 | SNP | Absolute | 5.43E-06 | T | C | CG43163 | intron variant | NA |
| 3L:8495741 | 3L:8488841 | SNP | Absolute | 5.43E-06 | T | C | CG43163 | intron variant | NA |
| 2R:10353132 | 2R:6240637 | SNP | Raw | 3.66E-06 | C | T | CG43171 | intergenic variant | NA |
| 2R:10353132 | 2R:6240637 | SNP | Raw | 3.66E-06 | C | T | CG43172 | intergenic variant | NA |
| 2R:10353132 | 2R:6240637 | SNP | Raw | 3.66E-06 | C | T | CG43178 | intergenic variant | NA |
| 2R:10353132 | 2R:6240637 | SNP | Raw | 3.66E-06 | C | T | CG43200 | intergenic variant | NA |
| 2R:10353132 | 2R:6240637 | SNP | Raw | 3.66E-06 | C | T | CG43201 | intergenic variant | NA |
| 2R:10353132 | 2R:6240637 | SNP | Raw | 3.66E-06 | C | T | CG43397 | intergenic variant | NA |
| 2L:1023181 | 2L:1023181 | SNP | Absolute | 3.26E-07 | C | G | CG4375 | intergenic variant | NA |
| 2L:1023254 | 2L:1023254 | SNP | Absolute | 3.26E-07 | C | T | CG4375 | intergenic variant | NA |
| X:10192303 | X:10086336 | SNP | Absolute | 6.14E-06 | C | T | CG43902 | missense variant | NA |
| X:10192303 | X:10086336 | SNP | Absolute | 6.14E-06 | C | T | CG43902 | missense variant | NA |
| 3R:26454823 | 3R:22280545 | SNP | Raw | 5.43E-06 | C | G | CG5447 | intergenic variant | hot |
| X:17433111 | X:17327144 | SNP | Absolute | 6.48E-06 | C | T | CG5613 | intergenic variant | NA |
| 2L:17961008 | 2L:17961008 | SNP | Raw | 3.67E-06 | T | A | CG5674 | intergenic variant | hot & cold |
| 2L:17969392 | 2L:17969392 | SNP | Raw | 6.31E-06 | A | G | CG5674 | synonymous variant | hot & cold |
| 2L:17969392 | 2L:17969392 | SNP | Raw | 6.31E-06 | A | G | CG5674 | intron variant | hot & cold |
| 2L:16310742 | 2L:16310742 | SNP | Absolute | 5.08E-06 | C | T | CG5861 | intergenic variant | NA |
| 3L:11947080 | 3L:11940180 | SNP | Absolute | 1.91E-06 | C | T | CG5883 | intergenic variant | cold |
| 3R:25030054 | 3R:20855776 | SNP | Absolute | 3.06E-06 | A | G | CG6422 | intergenic variant | NA |
| 3R:25030054 | 3R:20855776 | SNP | Absolute | 3.06E-06 | A | G | CG6422 | intergenic variant | NA |
| 2L:12025074 | 2L:12025074 | SNP | Raw | 8.29E-06 | C | T | CG6734 | intergenic variant | NA |
| 3L:11947080 | 3L:11940180 | SNP | Absolute | 1.91E-06 | C | T | CG7252 | intergenic variant | NA |
| 3R:14732067 | 3R:10557789 | SNP | Absolute | 2.55E-06 | C | T | CG7530 | intergenic variant | hot |
| 3R:14732067 | 3R:10557789 | SNP | Absolute | 2.55E-06 | C | T | CG7530 | intergenic variant | hot |
| 2R:16322619 | 2R:12210124 | SNP | Absolute | 6.37E-06 | A | C | CG8060 | intergenic variant | NA |
| 2R:16322619 | 2R:12210124 | SNP | Absolute | 6.37E-06 | A | C | CG8060 | intergenic variant | NA |
| 2R:16322619 | 2R:12210124 | SNP | Raw | 8.92E-06 | A | C | CG8060 | intergenic variant | NA |
| X:15694750 | X:15588783 | SNP | Raw | 4.56E-06 | T | G | CG8128 | intergenic variant | NA |
| 2R:12526102 | 2R:8413607 | SNP | Absolute | 2.42E-07 | C | T | CG8569 | intergenic variant | NA |
| 2R:12302662 | 2R:8190167 | INS | Absolute | 7.60E-08 | G | GGGATG | CG8850 | intergenic variant | NA |
| 2R:12302662 | 2R:8190167 | INS | Absolute | 7.60E-08 | G | GGGATG | CG8850 | intergenic variant | NA |
| 2R:12302662 | 2R:8190167 | INS | Raw | 1.32E-06 | G | GGGATG | CG8850 | intergenic variant | NA |
| 3R:9328010 | 3R:5153732 | SNP | Absolute | 9.19E-06 | A | G | CG8861 | intron variant | cold |
| 3R:9328010 | 3R:5153732 | SNP | Absolute | 9.19E-06 | A | G | CG8866 | intergenic variant | NA |
| X:16724373 | X:16618406 | INS | Absolute | 6.83E-06 | A | AA | CG9634 | intergenic variant | NA |
| 2R:23782137 | 2R:19669660 | SNP | Raw | 1.83E-07 | G | A | CG9850 | intron variant | NA |
| 2R:21072501 | 2R:16960006 | SNP | Absolute | 9.97E-06 | T | G | Cht9 | intergenic variant | NA |
| 2L:16310742 | 2L:16310742 | SNP | Absolute | 5.08E-06 | C | T | cni | intergenic variant | NA |
| 3L:15647417 | 3L:15640517 | SNP | Raw | 9.06E-06 | C | T | comm3 | synonymous variant | cold |
| 3R:13712326 | 3R:9538048 | SNP | Absolute | 1.77E-06 | A | G | CR34044 | intergenic variant | hot & cold |
| 3R:26325774 | 3R:22151496 | SNP | Absolute | 2.09E-07 | T | G | CR42765 | intergenic variant | NA |
| 3R:26325774 | 3R:22151496 | SNP | Raw | 8.64E-07 | T | G | CR42765 | intergenic variant | NA |
| X:15694750 | X:15588783 | SNP | Raw | 4.56E-06 | T | G | CR43132 | intergenic variant | NA |
| 2L:15524598 | 2L:15524598 | SNP | Absolute | 1.35E-07 | A | G | CR43761 | intergenic variant | cold |
| 3L:20727281 | 3L:20720381 | SNP | Absolute | 9.17E-06 | A | G | CR43945 | intergenic variant | NA |
| 3R:12726343 | 3R:8552065 | SNP | Absolute | 9.20E-06 | G | T | CR44231 | intergenic variant | NA |
| 3L:12665912 | 3L:12659012 | SNP | Raw | 8.21E-06 | C | T | CR44552 | intergenic variant | NA |
| 3L:12665916 | 3L:12659016 | SNP | Raw | 4.57E-06 | G | T | CR44552 | intergenic variant | NA |
| 2L:12025074 | 2L:12025074 | SNP | Raw | 8.29E-06 | C | T | CR44587 | non coding transcript/exon variant | NA |
| 2R:21072501 | 2R:16960006 | SNP | Absolute | 9.97E-06 | T | G | CR44703 | intergenic variant | NA |
| 2R:21072501 | 2R:16960006 | SNP | Absolute | 9.97E-06 | T | G | CR44704 | intergenic variant | NA |
| 3L:11250903 | 3L:11244003 | SNP | Absolute | 5.70E-06 | A | G | CR44713 | intergenic variant | NA |
| 3L:11250903 | 3L:11244003 | SNP | Absolute | 5.70E-06 | A | G | CR44713 | intergenic variant | NA |
| 3L:11250903 | 3L:11244003 | SNP | Absolute | 5.70E-06 | A | G | CR44714 | intergenic variant | NA |
| 3L:11250903 | 3L:11244003 | SNP | Absolute | 5.70E-06 | A | G | CR44714 | intergenic variant | NA |
| 3L:11250903 | 3L:11244003 | SNP | Absolute | 5.70E-06 | A | G | CR44715 | intergenic variant | NA |
| 3L:11250903 | 3L:11244003 | SNP | Absolute | 5.70E-06 | A | G | CR44715 | intergenic variant | NA |
| 3L:11250903 | 3L:11244003 | SNP | Absolute | 5.70E-06 | A | G | CR44716 | intergenic variant | NA |
| 3L:11250903 | 3L:11244003 | SNP | Absolute | 5.70E-06 | A | G | CR44716 | intergenic variant | NA |
| 3L:11250903 | 3L:11244003 | SNP | Absolute | 5.70E-06 | A | G | CR44717 | non coding transcript/exon variant | NA |
| 3L:11250903 | 3L:11244003 | SNP | Absolute | 5.70E-06 | A | G | CR44717 | non coding transcript/exon variant | NA |
| 2R:24326786 | 2R:20214309 | SNP | Raw | 5.04E-06 | T | A | CR44810 | intron variant | NA |
| 2R:24326786 | 2R:20214309 | SNP | Raw | 5.04E-06 | T | A | CR44810 | intergenic variant | NA |
| 2L:15512250 | 2L:15512250 | SNP | Absolute | 1.90E-08 | T | C | CR44864 | intergenic variant | hot |
| 2L:15512480 | 2L:15512480 | SNP | Absolute | 3.82E-06 | T | A | CR44864 | intergenic variant | hot |
| 2L:15512644 | 2L:15512644 | SNP | Absolute | 2.61E-06 | C | T | CR44864 | intergenic variant | hot |
| 2L:15513183 | 2L:15513183 | SNP | Absolute | 1.39E-06 | A | T | CR44864 | intergenic variant | hot |
| X:10192303 | X:10086336 | SNP | Absolute | 6.14E-06 | C | T | CR44894 | intron variant | cold |
| X:10192303 | X:10086336 | SNP | Absolute | 6.14E-06 | C | T | CR44894 | intron variant | cold |
| 2L:20590834 | 2L:20590834 | SNP | Raw | 7.11E-06 | C | A | CR44909 | intron variant | hot |
| 3R:26609244 | 3R:22434966 | SNP | Absolute | 5.34E-06 | C | T | CR44951 | intergenic variant | NA |
| 3R:26609244 | 3R:22434966 | SNP | Absolute | 5.34E-06 | C | T | CR44951 | intergenic variant | NA |
| X:22664164 | X:22066000 | SNP | Absolute | 7.13E-06 | C | G | CR44997 | intron variant | NA |
| 2R:12302662 | 2R:8190167 | INS | Absolute | 7.60E-08 | G | GGGATG | CR45271 | intergenic variant | NA |
| 2R:12302662 | 2R:8190167 | INS | Absolute | 7.60E-08 | G | GGGATG | CR45271 | intergenic variant | NA |
| 2R:12302662 | 2R:8190167 | INS | Raw | 1.32E-06 | G | GGGATG | CR45271 | intergenic variant | NA |
| 2R:13366781 | 2R:9254286 | SNP | Absolute | 3.62E-06 | T | A | CR45312 | intergenic variant | cold |
| 2R:13366781 | 2R:9254286 | SNP | Absolute | 3.62E-06 | T | A | CR45312 | intergenic variant | cold |
| 2R:13366782 | 2R:9254287 | SNP | Absolute | 5.61E-06 | A | T | CR45312 | intergenic variant | cold |
| 2R:13366782 | 2R:9254287 | SNP | Absolute | 5.61E-06 | A | T | CR45312 | intergenic variant | cold |
| X:15694750 | X:15588783 | SNP | Raw | 4.56E-06 | T | G | CR45523 | intergenic variant | NA |
| X:15694750 | X:15588783 | SNP | Raw | 4.56E-06 | T | G | CR45524 | intergenic variant | NA |
| X:15694750 | X:15588783 | SNP | Raw | 4.56E-06 | T | G | CR45526 | intergenic variant | NA |
| X:12460715 | X:12354748 | SNP | Absolute | 9.32E-07 | A | G | CR45615 | intergenic variant | NA |
| X:12460715 | X:12354748 | SNP | Absolute | 9.32E-07 | A | G | CR45615 | intergenic variant | NA |
| X:12460715 | X:12354748 | SNP | Raw | 8.95E-06 | A | G | CR45615 | intergenic variant | NA |
| X:12460721 | X:12354754 | SNP | Absolute | 4.94E-06 | G | A | CR45615 | intergenic variant | NA |
| X:12460721 | X:12354754 | SNP | Absolute | 4.94E-06 | G | A | CR45615 | intergenic variant | NA |
| X:12460725 | X:12354758 | SNP | Absolute | 2.54E-06 | C | T | CR45615 | intergenic variant | NA |
| X:12460725 | X:12354758 | SNP | Absolute | 2.54E-06 | C | T | CR45615 | intergenic variant | NA |
| X:12460908 | X:12354941 | SNP | Absolute | 1.81E-06 | C | G | CR45615 | intergenic variant | NA |
| X:12460908 | X:12354941 | SNP | Absolute | 1.81E-06 | C | G | CR45615 | intergenic variant | NA |
| 3R:26609244 | 3R:22434966 | SNP | Absolute | 5.34E-06 | C | T | CR45788 | intergenic variant | NA |
| 3R:26609244 | 3R:22434966 | SNP | Absolute | 5.34E-06 | C | T | CR45788 | intergenic variant | NA |
| 3R:19190327 | 3R:15016049 | SNP | Absolute | 4.88E-07 | A | G | CR46039 | intergenic variant | cold |
| 3R:19190327 | 3R:15016049 | SNP | Absolute | 4.88E-07 | A | G | CR46039 | intergenic variant | cold |
| 3R:23060285 | 3R:18886007 | SNP | Absolute | 2.69E-06 | T | A | CR46089 | intergenic variant | cold |
| 3R:23060285 | 3R:18886007 | SNP | Absolute | 2.69E-06 | T | A | CR46089 | intergenic variant | cold |
| 3L:8862712 | 3L:8855812 | SNP | Absolute | 2.81E-06 | A | G | dally | intron variant | cold |
| 3L:8862712 | 3L:8855812 | SNP | Absolute | 2.81E-06 | A | G | dally | intron variant | cold |
| 2L:6292687 | 2L:6292687 | SNP | Absolute | 2.37E-06 | A | G | Ddr | intergenic variant | cold |
| 2L:6292687 | 2L:6292687 | SNP | Absolute | 2.37E-06 | A | G | Ddr | intron variant | cold |
| 2L:6292687 | 2L:6292687 | SNP | Absolute | 2.37E-06 | A | G | Ddr | intergenic variant | cold |
| 2L:6292687 | 2L:6292687 | SNP | Absolute | 2.37E-06 | A | G | Ddr | intron variant | cold |
| 3R:5826778 | 3R:1652500 | SNP | Absolute | 4.87E-06 | C | T | dgrn | intergenic variant | NA |
| 3R:5826778 | 3R:1652500 | SNP | Absolute | 4.87E-06 | C | T | dgrn | intergenic variant | NA |
| 2R:24836296 | 2R:20723819 | SNP | Raw | 5.72E-07 | C | T | Dll | intergenic variant | NA |
| 2R:24836456 | 2R:20723979 | SNP | Raw | 4.64E-07 | A | G | Dll | intergenic variant | NA |
| 2L:12025074 | 2L:12025074 | SNP | Raw | 8.29E-06 | C | T | dmGlut | intergenic variant | NA |
| 2R:13062395 | 2R:8949900 | SNP | Absolute | 2.69E-06 | T | C | Drl-2 | intron variant | NA |
| 2R:13062395 | 2R:8949900 | SNP | Absolute | 2.69E-06 | T | C | Drl-2 | intron variant | NA |
| 3R:14732067 | 3R:10557789 | SNP | Absolute | 2.55E-06 | C | T | eff | intergenic variant | hot |
| 3R:14732067 | 3R:10557789 | SNP | Absolute | 2.55E-06 | C | T | eff | intergenic variant | hot |
| 3L:15647417 | 3L:15640517 | SNP | Raw | 9.06E-06 | C | T | Eig71Ea | intergenic variant | NA |
| 3L:15647417 | 3L:15640517 | SNP | Raw | 9.06E-06 | C | T | Eig71Eb | intergenic variant | NA |
| 3L:15647417 | 3L:15640517 | SNP | Raw | 9.06E-06 | C | T | Eig71Ec | intergenic variant | NA |
| 3L:17997317 | 3L:17990417 | SNP | Absolute | 2.08E-06 | C | A | Eip75B | intron variant | NA |
| 3L:17999272 | 3L:17992372 | SNP | Absolute | 5.27E-06 | C | A | Eip75B | 5 prime UTR variant | NA |
| 3L:17999272 | 3L:17992372 | SNP | Absolute | 5.27E-06 | C | A | Eip75B | intron variant | NA |
| 3L:18001645 | 3L:17994745 | SNP | Absolute | 6.06E-06 | A | T | Eip75B | intron variant | NA |
| 3L:18001645 | 3L:17994745 | SNP | Absolute | 6.06E-06 | A | T | Eip75B | intergenic variant | NA |
| 3L:18001988 | 3L:17995088 | SNP | Absolute | 9.94E-06 | C | T | Eip75B | intron variant | NA |
| 3L:18001988 | 3L:17995088 | SNP | Absolute | 9.94E-06 | C | T | Eip75B | intergenic variant | NA |
| 3L:18004874 | 3L:17997974 | SNP | Absolute | 4.98E-06 | A | G | Eip75B | intron variant | NA |
| 3L:18006774 | 3L:17999874 | SNP | Absolute | 1.32E-06 | T | G | Eip75B | intron variant | NA |
| 3L:18006921 | 3L:18000021 | SNP | Absolute | 1.15E-06 | A | G | Eip75B | intron variant | NA |
| 3L:18007991 | 3L:18001091 | SNP | Absolute | 1.07E-06 | C | T | Eip75B | intron variant | NA |
| 3L:18008173 | 3L:18001273 | SNP | Absolute | 1.07E-06 | G | A | Eip75B | intron variant | NA |
| 3L:18008461 | 3L:18001561 | SNP | Absolute | 9.99E-06 | C | T | Eip75B | intron variant | NA |
| 3L:18008537 | 3L:18001637 | SNP | Absolute | 3.31E-06 | C | T | Eip75B | intron variant | NA |
| 3L:18008556 | 3L:18001656 | SNP | Absolute | 1.53E-06 | G | A | Eip75B | intron variant | NA |
| 3L:18008562 | 3L:18001662 | SNP | Absolute | 1.53E-06 | T | A | Eip75B | intron variant | NA |
| 3L:18012599 | 3L:18005699 | SNP | Absolute | 1.90E-07 | G | T | Eip75B | intron variant | NA |
| 2R:12526102 | 2R:8413607 | SNP | Absolute | 2.42E-07 | C | T | fra | intergenic variant | cold |
| 2L:16310742 | 2L:16310742 | SNP | Absolute | 5.08E-06 | C | T | fzy | intergenic variant | NA |
| 3R:5826778 | 3R:1652500 | SNP | Absolute | 4.87E-06 | C | T | glob3 | intergenic variant | NA |
| 3R:5826778 | 3R:1652500 | SNP | Absolute | 4.87E-06 | C | T | glob3 | intergenic variant | NA |
| 3R:8150010 | 3R:3975732 | SNP | Absolute | 1.47E-06 | A | G | grn | intron variant | hot & cold |
| 3R:8150012 | 3R:3975734 | SNP | Absolute | 1.20E-06 | C | G | grn | intron variant | hot & cold |
| 2L:1595899 | 2L:1595899 | SNP | Raw | 4.45E-07 | C | G | haf | intron variant | cold |
| 3R:26454823 | 3R:22280545 | SNP | Raw | 5.43E-06 | C | G | Hex-t1 | intergenic variant | NA |
| 3R:26454823 | 3R:22280545 | SNP | Raw | 5.43E-06 | C | G | Hex-t2 | intergenic variant | NA |
| 3R:25030054 | 3R:20855776 | SNP | Absolute | 3.06E-06 | A | G | Hr96 | intergenic variant | NA |
| 3R:25030054 | 3R:20855776 | SNP | Absolute | 3.06E-06 | A | G | Hr96 | intergenic variant | NA |
| X:11108613 | X:11002646 | INS | Absolute | 3.80E-06 | G | GTAC | Hsp60 | 5 prime UTR variant | NA |
| X:11108613 | X:11002646 | INS | Absolute | 3.80E-06 | G | GTAC | Hsp60 | intergenic variant | NA |
| X:11108613 | X:11002646 | INS | Absolute | 3.80E-06 | G | GTAC | Hsp60 | 5 prime UTR variant | NA |
| X:11108613 | X:11002646 | INS | Absolute | 3.80E-06 | G | GTAC | Hsp60 | intergenic variant | NA |
| 2L:1023181 | 2L:1023181 | SNP | Absolute | 3.26E-07 | C | G | IA-2 | intron variant | NA |
| 2L:1023254 | 2L:1023254 | SNP | Absolute | 3.26E-07 | C | T | IA-2 | intron variant | NA |
| 2R:19515783 | 2R:15403288 | SNP | Absolute | 9.64E-06 | C | A | Ir56c | intergenic variant | NA |
| 2R:19515783 | 2R:15403288 | SNP | Absolute | 9.64E-06 | C | A | Ir56c | intergenic variant | NA |
| 2R:19515783 | 2R:15403288 | SNP | Absolute | 9.64E-06 | C | A | Ir56d | intergenic variant | NA |
| 2R:19515783 | 2R:15403288 | SNP | Absolute | 9.64E-06 | C | A | Ir56d | intergenic variant | NA |
| 3L:10428487 | 3L:10421587 | SNP | Absolute | 9.96E-07 | G | C | Ir67b | intergenic variant | NA |
| 3L:10428487 | 3L:10421587 | SNP | Absolute | 9.96E-07 | G | C | Ir67b | intergenic variant | NA |
| 3L:10428487 | 3L:10421587 | SNP | Absolute | 9.96E-07 | G | C | Ir67c | intergenic variant | NA |
| 3L:10428487 | 3L:10421587 | SNP | Absolute | 9.96E-07 | G | C | Ir67c | intergenic variant | NA |
| 2L:16815764 | 2L:16815764 | SNP | Absolute | 8.19E-06 | G | A | mdy | intergenic variant | cold |
| 2L:16818587 | 2L:16818587 | SNP | Absolute | 5.13E-06 | C | T | mdy | intron variant | cold |
| 3R:12719133 | 3R:8544855 | SNP | Absolute | 2.90E-07 | A | T | Men | intron variant | hot |
| 3R:12719133 | 3R:8544855 | SNP | Raw | 6.24E-06 | A | T | Men | intron variant | hot |
| 3R:12719163 | 3R:8544885 | SNP | Absolute | 7.99E-06 | A | T | Men | intron variant | hot |
| 3R:12720159 | 3R:8545881 | SNP | Absolute | 7.13E-07 | T | C | Men | intron variant | hot |
| 3R:12720159 | 3R:8545881 | SNP | Absolute | 7.13E-07 | T | C | Men | intergenic variant | hot |
| 3R:12720176 | 3R:8545898 | SNP | Absolute | 1.65E-06 | C | T | Men | intron variant | hot |
| 3R:12720176 | 3R:8545898 | SNP | Absolute | 1.65E-06 | C | T | Men | intergenic variant | hot |
| 3R:12720214 | 3R:8545936 | SNP | Absolute | 2.14E-06 | C | G | Men | intron variant | hot |
| 3R:12720214 | 3R:8545936 | SNP | Absolute | 2.14E-06 | C | G | Men | intergenic variant | hot |
| 3R:12720242 | 3R:8545964 | SNP | Absolute | 2.97E-06 | A | T | Men | intron variant | hot |
| 3R:12720242 | 3R:8545964 | SNP | Absolute | 2.97E-06 | A | T | Men | intergenic variant | hot |
| 3R:12720253 | 3R:8545975 | SNP | Absolute | 3.44E-06 | A | G | Men | intron variant | hot |
| 3R:12720253 | 3R:8545975 | SNP | Absolute | 3.44E-06 | A | G | Men | intergenic variant | hot |
| 3R:12720258 | 3R:8545980 | MNP | Absolute | 2.34E-06 | ACA | TTT | Men | intron variant | hot |
| 3R:12720258 | 3R:8545980 | MNP | Absolute | 2.34E-06 | ACA | TTT | Men | intergenic variant | hot |
| 3R:12726343 | 3R:8552065 | SNP | Absolute | 9.20E-06 | G | T | Men | intergenic variant | hot |
| 3L:11947080 | 3L:11940180 | SNP | Absolute | 1.91E-06 | C | T | mir-285 | intergenic variant | NA |
| 2L:5647089 | 2L:5647089 | SNP | Raw | 3.71E-06 | G | A | mir-964 | intergenic variant | NA |
| X:3159013 | X:3053046 | SNP | Absolute | 5.71E-06 | G | A | N | intron variant | cold |
| X:8276762 | X:8170795 | SNP | Absolute | 9.79E-06 | A | C | nAChRalpha3 | 3 prime UTR variant | NA |
| X:8276770 | X:8170803 | SNP | Absolute | 9.78E-08 | A | T | nAChRalpha3 | 3 prime UTR variant | NA |
| 2R:7162863 | 2R:3050368 | DEL | Raw | 4.04E-06 | T | TTCGGAT | nec | intergenic variant | NA |
| 2R:7983798 | 2R:3871303 | SNP | Raw | 5.31E-06 | G | T | Nup44A | intergenic variant | NA |
| 3R:26454823 | 3R:22280545 | SNP | Raw | 5.43E-06 | C | G | Pdf | intergenic variant | NA |
| 2R:7162863 | 2R:3050368 | DEL | Raw | 4.04E-06 | T | TTCGGAT | pk | intron variant | NA |
| 2R:7180821 | 2R:3068326 | SNP | Raw | 6.38E-06 | C | G | pk | intron variant | NA |
| 2L:9681582 | 2L:9681582 | SNP | Raw | 1.08E-06 | T | C | Pka-C1 | intergenic variant | NA |
| 2L:9681583 | 2L:9681583 | SNP | Raw | 1.26E-06 | C | T | Pka-C1 | intergenic variant | NA |
| 2L:9681602 | 2L:9681602 | SNP | Raw | 5.45E-07 | A | G | Pka-C1 | intergenic variant | NA |
| 2L:9681618 | 2L:9681618 | SNP | Raw | 1.91E-07 | C | T | Pka-C1 | intergenic variant | NA |
| X:15362032 | X:15256065 | SNP | Absolute | 3.65E-06 | G | A | Pp1-13C | intergenic variant | NA |
| X:15362032 | X:15256065 | SNP | Absolute | 3.65E-06 | G | A | Pp1-13C | intergenic variant | NA |
| X:15362032 | X:15256065 | SNP | Raw | 7.11E-06 | G | A | Pp1-13C | intergenic variant | NA |
| 3L:7461922 | 3L:7455022 | SNP | Absolute | 5.29E-06 | C | T | ppk26 | intergenic variant | NA |
| 3L:7461922 | 3L:7455022 | SNP | Absolute | 5.29E-06 | C | T | ppk26 | intergenic variant | NA |
| 3R:13712326 | 3R:9538048 | SNP | Absolute | 1.77E-06 | A | G | primo-1 | intergenic variant | NA |
| 3R:13712326 | 3R:9538048 | SNP | Absolute | 1.77E-06 | A | G | primo-2 | intergenic variant | NA |
| X:12460715 | X:12354748 | SNP | Absolute | 9.32E-07 | A | G | Rab40 | intron variant | NA |
| X:12460715 | X:12354748 | SNP | Absolute | 9.32E-07 | A | G | Rab40 | intron variant | NA |
| X:12460715 | X:12354748 | SNP | Raw | 8.95E-06 | A | G | Rab40 | intron variant | NA |
| X:12460721 | X:12354754 | SNP | Absolute | 4.94E-06 | G | A | Rab40 | intron variant | NA |
| X:12460721 | X:12354754 | SNP | Absolute | 4.94E-06 | G | A | Rab40 | intron variant | NA |
| X:12460725 | X:12354758 | SNP | Absolute | 2.54E-06 | C | T | Rab40 | intron variant | NA |
| X:12460725 | X:12354758 | SNP | Absolute | 2.54E-06 | C | T | Rab40 | intron variant | NA |
| X:12460908 | X:12354941 | SNP | Absolute | 1.81E-06 | C | G | Rab40 | intron variant | NA |
| X:12460908 | X:12354941 | SNP | Absolute | 1.81E-06 | C | G | Rab40 | intron variant | NA |
| 3L:11947080 | 3L:11940180 | SNP | Absolute | 1.91E-06 | C | T | Rpt4R | intergenic variant | cold |
| X:20699151 | X:20570178 | SNP | Raw | 5.91E-07 | G | T | run | intergenic variant | NA |
| 2R:21480414 | 2R:17367919 | DEL | Absolute | 7.59E-06 | CAC | C | Sara | intergenic variant | NA |
| 2R:21480414 | 2R:17367919 | DEL | Absolute | 7.59E-06 | CAC | C | Sara | intergenic variant | NA |
| 3R:23060285 | 3R:18886007 | SNP | Absolute | 2.69E-06 | T | A | sav | intron variant | cold |
| 3R:23060285 | 3R:18886007 | SNP | Absolute | 2.69E-06 | T | A | sav | intron variant | cold |
| 3L:11250903 | 3L:11244003 | SNP | Absolute | 5.70E-06 | A | G | scyl | intergenic variant | NA |
| 3L:11250903 | 3L:11244003 | SNP | Absolute | 5.70E-06 | A | G | scyl | intergenic variant | NA |
| 2R:21396062 | 2R:17283567 | SNP | Absolute | 5.34E-06 | T | A | Sdc | intron variant | NA |
| 2R:21413812 | 2R:17301317 | SNP | Absolute | 8.21E-06 | G | T | Sdc | intron variant | NA |
| 2R:21480414 | 2R:17367919 | DEL | Absolute | 7.59E-06 | CAC | C | Sdc | intron variant | NA |
| 2R:21480414 | 2R:17367919 | DEL | Absolute | 7.59E-06 | CAC | C | Sdc | intron variant | NA |
| 2R:13919442 | 2R:9806947 | SNP | Absolute | 1.96E-06 | A | G | shot | intron variant | NA |
| 2R:13919442 | 2R:9806947 | SNP | Absolute | 1.96E-06 | A | G | shot | intron variant | NA |
| 2R:12302662 | 2R:8190167 | INS | Absolute | 7.60E-08 | G | GGGATG | SIP2 | 3 prime UTR variant | NA |
| 2R:12302662 | 2R:8190167 | INS | Absolute | 7.60E-08 | G | GGGATG | SIP2 | 3 prime UTR variant | NA |
| 2R:12302662 | 2R:8190167 | INS | Raw | 1.32E-06 | G | GGGATG | SIP2 | 3 prime UTR variant | NA |
| X:5393202 | X:5287235 | SNP | Raw | 1.51E-06 | G | A | SK | intron variant | NA |
| 2R:24326786 | 2R:20214309 | SNP | Raw | 5.04E-06 | T | A | slbo | intergenic variant | NA |
| 2R:19515783 | 2R:15403288 | SNP | Absolute | 9.64E-06 | C | A | sm | intergenic variant | cold |
| 2R:19515783 | 2R:15403288 | SNP | Absolute | 9.64E-06 | C | A | sm | intergenic variant | cold |
| 3R:25030054 | 3R:20855776 | SNP | Absolute | 3.06E-06 | A | G | Smg6 | synonymous variant | NA |
| 3R:25030054 | 3R:20855776 | SNP | Absolute | 3.06E-06 | A | G | Smg6 | synonymous variant | NA |
| 3L:17999272 | 3L:17992372 | SNP | Absolute | 5.27E-06 | C | A | snoRNA:Me28S-A30 | intergenic variant | NA |
| 3L:18001645 | 3L:17994745 | SNP | Absolute | 6.06E-06 | A | T | snoRNA:Me28S-A30 | intergenic variant | NA |
| 3L:18001988 | 3L:17995088 | SNP | Absolute | 9.94E-06 | C | T | snoRNA:Me28S-A30 | intergenic variant | NA |
| 3L:18004874 | 3L:17997974 | SNP | Absolute | 4.98E-06 | A | G | snoRNA:Me28S-A30 | intergenic variant | NA |
| 3L:18006774 | 3L:17999874 | SNP | Absolute | 1.32E-06 | T | G | snoRNA:Me28S-A30 | intergenic variant | NA |
| 3L:18006921 | 3L:18000021 | SNP | Absolute | 1.15E-06 | A | G | snoRNA:Me28S-A30 | intergenic variant | NA |
| 3L:18007991 | 3L:18001091 | SNP | Absolute | 1.07E-06 | C | T | snoRNA:Me28S-A30 | intergenic variant | NA |
| 3L:16655829 | 3L:16648929 | SNP | Absolute | 6.45E-07 | A | G | snRNA:U12:73B | intergenic variant | NA |
| 3L:16655829 | 3L:16648929 | SNP | Absolute | 6.45E-07 | A | G | snRNA:U12:73B | intergenic variant | NA |
| 2L:20331015 | 2L:20331015 | SNP | Raw | 3.21E-06 | T | A | spir | intron variant | hot |
| 2L:20331045 | 2L:20331045 | SNP | Raw | 4.37E-06 | G | A | spir | intron variant | hot |
| 2L:16310742 | 2L:16310742 | SNP | Absolute | 5.08E-06 | C | T | Syx5 | intergenic variant | NA |
| 2R:21072501 | 2R:16960006 | SNP | Absolute | 9.97E-06 | T | G | Treh | intergenic variant | NA |
| 3L:7461922 | 3L:7455022 | SNP | Absolute | 5.29E-06 | C | T | Tsp66A | intergenic variant | NA |
| 3L:7461922 | 3L:7455022 | SNP | Absolute | 5.29E-06 | C | T | Tsp66A | intergenic variant | NA |
| 3R:17742322 | 3R:13568044 | SNP | Absolute | 6.69E-06 | C | T | TyrRII | intron variant | hot |
| 3R:17742322 | 3R:13568044 | SNP | Absolute | 6.69E-06 | C | T | TyrRII | intron variant | hot |
| 3R:17742348 | 3R:13568070 | INS | Absolute | 4.30E-06 | G | GT | TyrRII | intron variant | hot |
| 3R:17742348 | 3R:13568070 | INS | Absolute | 4.30E-06 | G | GT | TyrRII | intron variant | hot |
| 3R:23060285 | 3R:18886007 | SNP | Absolute | 2.69E-06 | T | A | Ublcp1 | intergenic variant | cold |
| 3R:23060285 | 3R:18886007 | SNP | Absolute | 2.69E-06 | T | A | Ublcp1 | intergenic variant | cold |
| 2R:16322619 | 2R:12210124 | SNP | Absolute | 6.37E-06 | A | C | Vha44 | intergenic variant | NA |
| 2R:16322619 | 2R:12210124 | SNP | Absolute | 6.37E-06 | A | C | Vha44 | intergenic variant | NA |
| 2R:16322619 | 2R:12210124 | SNP | Raw | 8.92E-06 | A | C | Vha44 | intergenic variant | NA |
| 2L:14310715 | 2L:14310715 | SNP | Raw | 8.22E-06 | T | C | wb | intron variant | hot & cold |
| 2L:14310739 | 2L:14310739 | SNP | Raw | 7.64E-06 | C | A | wb | intron variant | hot & cold |
