# Supplementary material for: Genetic basis of thermal plasticity variation in Drosophila melanogaster body size
Source: PLoS Genet. 2018 Sep 26;14(9):e1007686. doi: 10.1371/journal.pgen.1007686 (PMC6175520; doi:10.1371/journal.pgen.1007686)
Supplement: S4 Table — (DOCX) [file pgen.1007686.s011.docx]

| **S4 Table. GWAS results for variation in size.**  Nominally significant SNPs (p-value < 10e-5) from GWAS for size variation at 17°C and at 28°C per body part. The genomic position (given by annotation with Genome Releases v.5. and v.6.), type of SNP/Indel, potential impact, associated gene name (Flybase Gene ID) and putative consequence are also shown. | | | | | | | | |
| --- | --- | --- | --- | --- | --- | --- | --- | --- |
| **Genomic position (v.6.)** | **Genomic position (v.5.)** | **SNP/**  **Indel** | **Temperature** | **P value** | **Major allele** | **Minor allele** | **Gene symbol** | **Affected region** |
| 2L:10191206 | 2L:10191206 | SNP | 28C | 3.21E-06 | G | A | CG31874 | intron variant |
| 2L:10191206 | 2L:10191206 | SNP | 28C | 3.21E-06 | G | A | Sur | intron variant |
| 2L:10365704 | 2L:10365704 | SNP | 17C | 7.08E-06 | A | G | Cand1 | intergenic variant |
| 2L:10365704 | 2L:10365704 | SNP | 17C | 7.08E-06 | A | G | CG5056 | intergenic variant |
| 2L:10365704 | 2L:10365704 | SNP | 17C | 7.08E-06 | A | G | lft | intron variant |
| 2L:10365704 | 2L:10365704 | SNP | 17C | 7.08E-06 | A | G | pim | intergenic variant |
| 2L:10365704 | 2L:10365704 | SNP | 17C | 7.08E-06 | A | G | rho-5 | intergenic variant |
| 2L:10727825 | 2L:10727825 | SNP | 28C | 5.61E-06 | T | C | CG17118 | intergenic variant |
| 2L:10727825 | 2L:10727825 | SNP | 28C | 5.61E-06 | T | C | CG6431 | intron variant |
| 2L:10727825 | 2L:10727825 | SNP | 28C | 5.61E-06 | T | C | CG6443 | intergenic variant |
| 2L:10727825 | 2L:10727825 | SNP | 28C | 5.61E-06 | T | C | CG6750 | intergenic variant |
| 2L:10727825 | 2L:10727825 | SNP | 28C | 5.61E-06 | T | C | Dpy-30L1 | intergenic variant |
| 2L:14572053 | 2L:14572053 | SNP | 17C | 9.68E-06 | C | T | CR44856 | intergenic variant |
| 2L:17421687 | 2L:17421687 | SNP | 17C | 9.61E-06 | G | T | Dif | intron variant |
| 2L:17433046 | 2L:17433046 | SNP | 17C | 2.27E-06 | T | A | CG33928 | intergenic variant |
| 2L:17433046 | 2L:17433046 | SNP | 17C | 2.27E-06 | T | A | CG5043 | intergenic variant |
| 2L:17433046 | 2L:17433046 | SNP | 17C | 2.27E-06 | T | A | CR44409 | intergenic variant |
| 2L:17433046 | 2L:17433046 | SNP | 17C | 2.27E-06 | T | A | Dif | 5 prime UTR variant |
| 2L:17433046 | 2L:17433046 | SNP | 17C | 2.27E-06 | T | A | Dif | intergenic variant |
| 2L:17433046 | 2L:17433046 | SNP | 17C | 2.27E-06 | T | A | dl | intergenic variant |
| 2L:17517353 | 2L:17517353 | SNP | 17C | 9.32E-06 | G | T | CG15143 | intergenic variant |
| 2L:17517353 | 2L:17517353 | SNP | 17C | 9.32E-06 | G | T | Dhc36C | intron variant |
| 2L:4353236 | 2L:4353236 | SNP | 28C | 1.59E-06 | A | G | CG15429 | intron variant |
| 2L:4353236 | 2L:4353236 | SNP | 28C | 1.59E-06 | A | G | CR44698 | intergenic variant |
| 2L:4353236 | 2L:4353236 | SNP | 28C | 1.59E-06 | A | G | CR44699 | intergenic variant |
| 2L:4353236 | 2L:4353236 | SNP | 28C | 1.59E-06 | A | G | CR44700 | intergenic variant |
| 2L:7866740 | 2L:7866740 | SNP | 17C | 9.07E-06 | T | A | Proc | intron variant |
| 2L:8978795 | 2L:8978795 | SNP | 28C | 4.01E-06 | C | T | CG13101 | intron variant |
| 2L:8978795 | 2L:8978795 | SNP | 28C | 4.01E-06 | C | T | CG18662 | intergenic variant |
| 2L:8978795 | 2L:8978795 | SNP | 28C | 4.01E-06 | C | T | CG9541 | intergenic variant |
| 2L:9645469 | 2L:9645469 | SNP | 17C | 6.70E-06 | C | G | Apoltp | synonymous variant |
| 2L:9645535 | 2L:9645535 | SNP | 17C | 7.79E-06 | A | T | Apoltp | synonymous variant |
| 2L:9649060 | 2L:9649060 | SNP | 17C | 4.60E-06 | C | T | Apoltp | synonymous variant |
| 2L:9649237 | 2L:9649237 | SNP | 17C | 3.96E-06 | T | G | Apoltp | synonymous variant |
| 2L:9751781 | 2L:9751781 | SNP | 28C | 8.00E-06 | T | C | Cyp4e3 | intergenic variant |
| 2R:10243133 | 2R:6130638 | SNP | 28C | 5.54E-06 | A | T | CG12209 | intergenic variant |
| 2R:10243133 | 2R:6130638 | SNP | 28C | 5.54E-06 | A | T | CG12914 | intergenic variant |
| 2R:10243133 | 2R:6130638 | SNP | 28C | 5.54E-06 | A | T | CR45265 | intergenic variant |
| 2R:10243133 | 2R:6130638 | SNP | 28C | 5.54E-06 | A | T | Hdc | intron variant |
| 2R:10525929 | 2R:6413434 | SNP | 17C | 6.95E-06 | G | A | lola | intergenic variant |
| 2R:10525929 | 2R:6413434 | SNP | 17C | 6.95E-06 | G | A | lola | intron variant |
| 2R:11396623 | 2R:7284128 | SNP | 17C | 2.02E-06 | A | T | sprt | intron variant |
| 2R:11396623 | 2R:7284128 | SNP | 17C | 2.02E-06 | A | T | tRNA:CR30257 | intergenic variant |
| 2R:11396623 | 2R:7284128 | SNP | 17C | 2.02E-06 | A | T | tRNA:CR30506 | intergenic variant |
| 2R:11434678 | 2R:7322183 | SNP | 17C | 6.78E-06 | A | T | CG7777 | intergenic variant |
| 2R:11434678 | 2R:7322183 | SNP | 17C | 6.78E-06 | A | T | CR43771 | intergenic variant |
| 2R:11434684 | 2R:7322189 | SNP | 17C | 4.58E-06 | A | G | CG7777 | intergenic variant |
| 2R:11434684 | 2R:7322189 | SNP | 17C | 4.58E-06 | A | G | CR43771 | intergenic variant |
| 2R:12302662 | 2R:8190167 | INS | 28C | 8.00E-06 | G | GGGATG | CG17739 | intergenic variant |
| 2R:12302662 | 2R:8190167 | INS | 28C | 8.00E-06 | G | GGGATG | CG42700 | intergenic variant |
| 2R:12302662 | 2R:8190167 | INS | 28C | 8.00E-06 | G | GGGATG | CG8850 | intergenic variant |
| 2R:12302662 | 2R:8190167 | INS | 28C | 8.00E-06 | G | GGGATG | CR45271 | intergenic variant |
| 2R:12302662 | 2R:8190167 | INS | 28C | 8.00E-06 | G | GGGATG | SIP2 | 3 prime UTR variant |
| 2R:12618589 | 2R:8506094 | SNP | 17C | 6.86E-06 | C | T | CG45086 | intron variant |
| 2R:12618589 | 2R:8506094 | SNP | 17C | 6.86E-06 | C | T | Galphaq | intron variant |
| 2R:12717813 | 2R:8605318 | SNP | 17C | 1.27E-06 | G | T | CG42663 | intron variant |
| 2R:12717820 | 2R:8605325 | SNP | 17C | 8.37E-06 | A | C | CG42663 | intron variant |
| 2R:13146124 | 2R:9033629 | SNP | 17C | 5.27E-06 | T | A | Ack-like | 3 prime UTR variant |
| 2R:13146124 | 2R:9033629 | SNP | 17C | 5.27E-06 | T | A | Ack-like | intergenic variant |
| 2R:13146124 | 2R:9033629 | SNP | 17C | 5.27E-06 | T | A | Ack-like | intron variant |
| 2R:13146124 | 2R:9033629 | SNP | 17C | 5.27E-06 | T | A | Cap-G | intergenic variant |
| 2R:13771344 | 2R:9658849 | SNP | 17C | 8.83E-06 | C | T | Cpr50Ca | intron variant |
| 2R:16055909 | 2R:11943414 | SNP | 28C | 5.82E-06 | G | C | CG8405 | intron variant |
| 2R:17657443 | 2R:13544948 | SNP | 17C | 6.04E-06 | A | G | CCHa1-R | intron variant |
| 2R:17657462 | 2R:13544967 | DEL | 17C | 6.04E-06 | AG | A | CCHa1-R | intron variant |
| 2R:18058763 | 2R:13946268 | SNP | 17C | 1.43E-06 | A | G | CG10911 | intergenic variant |
| 2R:18058763 | 2R:13946268 | SNP | 17C | 1.43E-06 | A | G | CG10912 | intergenic variant |
| 2R:18058763 | 2R:13946268 | SNP | 17C | 1.43E-06 | A | G | CG34386 | intergenic variant |
| 2R:18058763 | 2R:13946268 | SNP | 17C | 1.43E-06 | A | G | CR45313 | intergenic variant |
| 2R:18058763 | 2R:13946268 | SNP | 17C | 1.43E-06 | A | G | CR45314 | non coding transcript/exon variant |
| 2R:18058774 | 2R:13946279 | SNP | 17C | 2.61E-06 | G | T | CG10911 | intergenic variant |
| 2R:18058774 | 2R:13946279 | SNP | 17C | 2.61E-06 | G | T | CG10912 | intergenic variant |
| 2R:18058774 | 2R:13946279 | SNP | 17C | 2.61E-06 | G | T | CG34386 | intergenic variant |
| 2R:18058774 | 2R:13946279 | SNP | 17C | 2.61E-06 | G | T | CR45313 | intergenic variant |
| 2R:18058774 | 2R:13946279 | SNP | 17C | 2.61E-06 | G | T | CR45314 | non coding transcript/exon variant |
| 2R:18058791 | 2R:13946296 | SNP | 17C | 9.62E-06 | C | T | CG10911 | intergenic variant |
| 2R:18058791 | 2R:13946296 | SNP | 17C | 9.62E-06 | C | T | CG10912 | intergenic variant |
| 2R:18058791 | 2R:13946296 | SNP | 17C | 9.62E-06 | C | T | CG34386 | intergenic variant |
| 2R:18058791 | 2R:13946296 | SNP | 17C | 9.62E-06 | C | T | CR45313 | intergenic variant |
| 2R:18058791 | 2R:13946296 | SNP | 17C | 9.62E-06 | C | T | CR45314 | non coding transcript/exon variant |
| 2R:18058817 | 2R:13946322 | SNP | 17C | 9.52E-07 | A | G | CG10911 | intergenic variant |
| 2R:18058817 | 2R:13946322 | SNP | 17C | 9.52E-07 | A | G | CG10912 | intergenic variant |
| 2R:18058817 | 2R:13946322 | SNP | 17C | 9.52E-07 | A | G | CG34386 | intergenic variant |
| 2R:18058817 | 2R:13946322 | SNP | 17C | 9.52E-07 | A | G | CR45313 | intergenic variant |
| 2R:18058817 | 2R:13946322 | SNP | 17C | 9.52E-07 | A | G | CR45314 | non coding transcript/exon variant |
| 2R:19503880 | 2R:15391385 | SNP | 17C | 5.43E-06 | C | T | CG11007 | synonymous variant |
| 2R:19503880 | 2R:15391385 | SNP | 17C | 5.43E-06 | C | T | CG15120 | intergenic variant |
| 2R:19503880 | 2R:15391385 | SNP | 17C | 5.43E-06 | C | T | CG16926 | intergenic variant |
| 2R:19503880 | 2R:15391385 | SNP | 17C | 5.43E-06 | C | T | CR44472 | intergenic variant |
| 2R:19503880 | 2R:15391385 | SNP | 17C | 5.43E-06 | C | T | Ir56b | intergenic variant |
| 2R:19503880 | 2R:15391385 | SNP | 17C | 5.43E-06 | C | T | tRNA:H:56E | intergenic variant |
| 2R:19504481 | 2R:15391986 | SNP | 17C | 9.88E-07 | G | A | CG11007 | 3 prime UTR variant |
| 2R:19504481 | 2R:15391986 | SNP | 17C | 9.88E-07 | G | A | CG11007 | intergenic variant |
| 2R:19504481 | 2R:15391986 | SNP | 17C | 9.88E-07 | G | A | CG15120 | intergenic variant |
| 2R:19504481 | 2R:15391986 | SNP | 17C | 9.88E-07 | G | A | CG16926 | intergenic variant |
| 2R:19504481 | 2R:15391986 | SNP | 17C | 9.88E-07 | G | A | CR44472 | intergenic variant |
| 2R:19504481 | 2R:15391986 | SNP | 17C | 9.88E-07 | G | A | Ir56b | intergenic variant |
| 2R:19504481 | 2R:15391986 | SNP | 17C | 9.88E-07 | G | A | tRNA:H:56E | intergenic variant |
| 2R:20012462 | 2R:15899967 | SNP | 17C | 3.11E-06 | G | A | CR44628 | intergenic variant |
| 2R:20012462 | 2R:15899967 | SNP | 17C | 3.11E-06 | G | A | CR44629 | intergenic variant |
| 2R:20012462 | 2R:15899967 | SNP | 17C | 3.11E-06 | G | A | CR44630 | intergenic variant |
| 2R:20012463 | 2R:15899968 | SNP | 17C | 8.60E-07 | G | A | CR44628 | intergenic variant |
| 2R:20012463 | 2R:15899968 | SNP | 17C | 8.60E-07 | G | A | CR44629 | intergenic variant |
| 2R:20012463 | 2R:15899968 | SNP | 17C | 8.60E-07 | G | A | CR44630 | intergenic variant |
| 2R:20012537 | 2R:15900042 | SNP | 17C | 3.19E-06 | A | T | CR44628 | intergenic variant |
| 2R:20012537 | 2R:15900042 | SNP | 17C | 3.19E-06 | A | T | CR44629 | intergenic variant |
| 2R:20012537 | 2R:15900042 | SNP | 17C | 3.19E-06 | A | T | CR44630 | intergenic variant |
| 2R:20012544 | 2R:15900049 | SNP | 17C | 5.88E-06 | A | G | CR44628 | intergenic variant |
| 2R:20012544 | 2R:15900049 | SNP | 17C | 5.88E-06 | A | G | CR44629 | intergenic variant |
| 2R:20012544 | 2R:15900049 | SNP | 17C | 5.88E-06 | A | G | CR44630 | intergenic variant |
| 2R:20012550 | 2R:15900055 | SNP | 17C | 3.54E-06 | A | T | CR44628 | intergenic variant |
| 2R:20012550 | 2R:15900055 | SNP | 17C | 3.54E-06 | A | T | CR44629 | intergenic variant |
| 2R:20012550 | 2R:15900055 | SNP | 17C | 3.54E-06 | A | T | CR44630 | intergenic variant |
| 2R:20013356 | 2R:15900861 | SNP | 17C | 9.48E-06 | T | C | CR44629 | intergenic variant |
| 2R:20013356 | 2R:15900861 | SNP | 17C | 9.48E-06 | T | C | CR44630 | intergenic variant |
| 2R:20936608 | 2R:16824113 | SNP | 17C | 1.00E-06 | G | A | Rx | missense variant |
| 2R:20987752 | 2R:16875257 | SNP | 28C | 1.07E-06 | G | T | CG15651 | intergenic variant |
| 2R:20987752 | 2R:16875257 | SNP | 28C | 1.07E-06 | G | T | CG3216 | synonymous variant |
| 2R:20987752 | 2R:16875257 | SNP | 28C | 1.07E-06 | G | T | CG9313 | intergenic variant |
| 2R:20987752 | 2R:16875257 | SNP | 28C | 1.07E-06 | G | T | dgt3 | intergenic variant |
| 2R:22173819 | 2R:18061324 | SNP | 17C | 9.12E-06 | G | T | a | intergenic variant |
| 2R:22173819 | 2R:18061324 | SNP | 17C | 9.12E-06 | G | T | CG11170 | intergenic variant |
| 2R:22173819 | 2R:18061324 | SNP | 17C | 9.12E-06 | G | T | CG3045 | synonymous variant |
| 2R:22173819 | 2R:18061324 | SNP | 17C | 9.12E-06 | G | T | CG6758 | intergenic variant |
| 2R:22173819 | 2R:18061324 | SNP | 17C | 9.12E-06 | G | T | CR45151 | intergenic variant |
| 2R:23737609 | 2R:19625132 | SNP | 17C | 5.21E-06 | C | T | egl | missense variant |
| 2R:23737609 | 2R:19625132 | SNP | 17C | 5.21E-06 | C | T | mir-4979 | intergenic variant |
| 2R:23737609 | 2R:19625132 | SNP | 17C | 5.21E-06 | C | T | Sesn | intergenic variant |
| 2R:23737625 | 2R:19625148 | SNP | 17C | 2.86E-06 | G | A | egl | synonymous variant |
| 2R:23737625 | 2R:19625148 | SNP | 17C | 2.86E-06 | G | A | mir-4979 | intergenic variant |
| 2R:23737625 | 2R:19625148 | SNP | 17C | 2.86E-06 | G | A | Sesn | intergenic variant |
| 2R:24326786 | 2R:20214309 | SNP | 17C | 9.88E-06 | T | A | CR44810 | intron variant |
| 2R:24326786 | 2R:20214309 | SNP | 17C | 9.88E-06 | T | A | CR44810 | intergenic variant |
| 2R:24326786 | 2R:20214309 | SNP | 17C | 9.88E-06 | T | A | slbo | intergenic variant |
| 2R:25171571 | 2R:21059094 | SNP | 17C | 2.31E-06 | C | T | CG34038 | intergenic variant |
| 2R:25171571 | 2R:21059094 | SNP | 17C | 2.31E-06 | C | T | Peb | intergenic variant |
| 2R:25171571 | 2R:21059094 | SNP | 17C | 2.31E-06 | C | T | PebII | intergenic variant |
| 2R:25171571 | 2R:21059094 | SNP | 17C | 2.31E-06 | C | T | Sfp60F | intergenic variant |
| 2R:25171743 | 2R:21059266 | SNP | 17C | 4.91E-06 | G | A | CG34038 | intergenic variant |
| 2R:25171743 | 2R:21059266 | SNP | 17C | 4.91E-06 | G | A | Peb | intergenic variant |
| 2R:25171743 | 2R:21059266 | SNP | 17C | 4.91E-06 | G | A | PebII | intergenic variant |
| 2R:25171743 | 2R:21059266 | SNP | 17C | 4.91E-06 | G | A | Sfp60F | intergenic variant |
| 2R:25171769 | 2R:21059292 | SNP | 17C | 4.03E-06 | G | A | CG34038 | intergenic variant |
| 2R:25171769 | 2R:21059292 | SNP | 17C | 4.03E-06 | G | A | Peb | intergenic variant |
| 2R:25171769 | 2R:21059292 | SNP | 17C | 4.03E-06 | G | A | PebII | intergenic variant |
| 2R:25171769 | 2R:21059292 | SNP | 17C | 4.03E-06 | G | A | Sfp60F | intergenic variant |
| 2R:25191934 | 2R:21079457 | SNP | 17C | 7.20E-06 | T | A | CG9380 | intron variant |
| 2R:25191934 | 2R:21079457 | SNP | 17C | 7.20E-06 | T | A | CG9380 | intergenic variant |
| 2R:25256280 | 2R:21143803 | SNP | 17C | 7.83E-06 | A | T | CG30428 | intergenic variant |
| 2R:25256280 | 2R:21143803 | SNP | 17C | 7.83E-06 | A | T | CG9380 | intergenic variant |
| 2R:4902906 | 2R:790411 | SNP | 17C | 4.58E-06 | C | T | CG44102 | intron variant |
| 2R:5744162 | 2R:1631667 | SNP | 17C | 2.34E-06 | A | C | - | intergenic variant |
| 2R:5744166 | 2R:1631671 | SNP | 17C | 2.01E-06 | T | C | - | intergenic variant |
| 2R:6552411 | 2R:2439916 | SNP | 17C | 1.63E-07 | A | G | jing | intron variant |
| 2R:6647012 | 2R:2534517 | SNP | 17C | 1.36E-07 | G | A | Bap170 | intergenic variant |
| 2R:6647012 | 2R:2534517 | SNP | 17C | 1.36E-07 | G | A | Debcl | intergenic variant |
| 2R:6647012 | 2R:2534517 | SNP | 17C | 1.36E-07 | G | A | Fmo-2 | intergenic variant |
| 2R:6648219 | 2R:2535724 | SNP | 17C | 1.86E-06 | T | A | Debcl | intergenic variant |
| 2R:6648219 | 2R:2535724 | SNP | 17C | 1.86E-06 | T | A | Fmo-2 | intergenic variant |
| 2R:6648219 | 2R:2535724 | SNP | 17C | 1.86E-06 | T | A | Opbp | intergenic variant |
| 2R:6649185 | 2R:2536690 | SNP | 17C | 1.20E-07 | C | T | Debcl | 5 prime UTR variant |
| 2R:6649185 | 2R:2536690 | SNP | 17C | 1.20E-07 | C | T | Fmo-2 | intergenic variant |
| 2R:6649185 | 2R:2536690 | SNP | 17C | 1.20E-07 | C | T | Opbp | intergenic variant |
| 2R:6649718 | 2R:2537223 | INS | 17C | 9.22E-08 | T | TG | Debcl | intron variant |
| 2R:6649718 | 2R:2537223 | INS | 17C | 9.22E-08 | T | TG | Fmo-2 | intergenic variant |
| 2R:6649718 | 2R:2537223 | INS | 17C | 9.22E-08 | T | TG | Opbp | intergenic variant |
| 2R:6649723 | 2R:2537228 | INS | 17C | 9.22E-08 | G | TCAAAAA | Debcl | intron variant |
| 2R:6649723 | 2R:2537228 | INS | 17C | 9.22E-08 | G | TCAAAAA | Fmo-2 | intergenic variant |
| 2R:6649723 | 2R:2537228 | INS | 17C | 9.22E-08 | G | TCAAAAA | Opbp | intergenic variant |
| 2R:6651687 | 2R:2539192 | SNP | 17C | 3.22E-06 | G | A | Debcl | intron variant |
| 2R:6651687 | 2R:2539192 | SNP | 17C | 3.22E-06 | G | A | geminin | intergenic variant |
| 2R:6651687 | 2R:2539192 | SNP | 17C | 3.22E-06 | G | A | Opbp | intergenic variant |
| 2R:6655630 | 2R:2543135 | DEL | 17C | 5.30E-06 | AA | A | Debcl | intergenic variant |
| 2R:6655630 | 2R:2543135 | DEL | 17C | 5.30E-06 | AA | A | geminin | 3 prime UTR variant |
| 2R:6655630 | 2R:2543135 | DEL | 17C | 5.30E-06 | AA | A | Opbp | intergenic variant |
| 2R:6655630 | 2R:2543135 | DEL | 17C | 5.30E-06 | AA | A | sced | intergenic variant |
| 2R:6655690 | 2R:2543195 | SNP | 17C | 3.22E-06 | C | A | Debcl | intergenic variant |
| 2R:6655690 | 2R:2543195 | SNP | 17C | 3.22E-06 | C | A | Dpit47 | intergenic variant |
| 2R:6655690 | 2R:2543195 | SNP | 17C | 3.22E-06 | C | A | geminin | missense variant |
| 2R:6655690 | 2R:2543195 | SNP | 17C | 3.22E-06 | C | A | Opbp | intergenic variant |
| 2R:6655690 | 2R:2543195 | SNP | 17C | 3.22E-06 | C | A | sced | intergenic variant |
| 2R:6829956 | 2R:2717461 | SNP | 17C | 3.73E-06 | G | T | CG15236 | missense variant |
| 2R:6829956 | 2R:2717461 | SNP | 17C | 3.73E-06 | G | T | CG34215 | intergenic variant |
| 2R:6861727 | 2R:2749232 | SNP | 17C | 8.96E-06 | A |  | coro | intergenic variant |
| 2R:6862773 | 2R:2750278 | SNP | 17C | 6.78E-07 | G | A | coro | 3 prime UTR variant |
| 2R:6862828 | 2R:2750333 | SNP | 17C | 3.68E-07 | C | T | coro | 3 prime UTR variant |
| 2R:6865972 | 2R:2753477 | SNP | 17C | 2.69E-06 | A | G | coro | intron variant |
| 2R:6865974 | 2R:2753479 | SNP | 17C | 4.14E-07 | C | T | coro | intron variant |
| 2R:6874627 | 2R:2762132 | SNP | 17C | 8.48E-06 | C |  | CG9447 | synonymous variant |
| 2R:6874627 | 2R:2762132 | SNP | 17C | 8.48E-06 | C |  | coro | intergenic variant |
| 2R:6874627 | 2R:2762132 | SNP | 17C | 8.48E-06 | C |  | CR44169 | intron variant |
| 2R:6874627 | 2R:2762132 | SNP | 17C | 8.48E-06 | C |  | CR44169 | intergenic variant |
| 2R:6874627 | 2R:2762132 | SNP | 17C | 8.48E-06 | C |  | Spn42Da | intergenic variant |
| 2R:6874627 | 2R:2762132 | SNP | 17C | 8.48E-06 | C |  | Spn42Db | intergenic variant |
| 2R:6879309 | 2R:2766814 | SNP | 17C | 1.66E-06 | G |  | CG9447 | intergenic variant |
| 2R:6879309 | 2R:2766814 | SNP | 17C | 1.66E-06 | G |  | CR44169 | intron variant |
| 2R:6879309 | 2R:2766814 | SNP | 17C | 1.66E-06 | G |  | Spn42Da | intergenic variant |
| 2R:6879309 | 2R:2766814 | SNP | 17C | 1.66E-06 | G |  | Spn42Db | 3 prime UTR variant |
| 2R:6879309 | 2R:2766814 | SNP | 17C | 1.66E-06 | G |  | Spn42Dc | intergenic variant |
| 2R:6879309 | 2R:2766814 | SNP | 17C | 1.66E-06 | G |  | Spn42Dd | intergenic variant |
| 2R:6880733 | 2R:2768238 | SNP | 17C | 3.99E-06 | A | C | CG9447 | intergenic variant |
| 2R:6880733 | 2R:2768238 | SNP | 17C | 3.99E-06 | A | C | CR44169 | intergenic variant |
| 2R:6880733 | 2R:2768238 | SNP | 17C | 3.99E-06 | A | C | Spn42Da | intergenic variant |
| 2R:6880733 | 2R:2768238 | SNP | 17C | 3.99E-06 | A | C | Spn42Db | 5 prime UTR variant |
| 2R:6880733 | 2R:2768238 | SNP | 17C | 3.99E-06 | A | C | Spn42Dc | intergenic variant |
| 2R:6880733 | 2R:2768238 | SNP | 17C | 3.99E-06 | A | C | Spn42Dd | intergenic variant |
| 2R:6880733 | 2R:2768238 | SNP | 17C | 3.99E-06 | A | C | Spn42De | intergenic variant |
| 2R:6881286 | 2R:2768791 | SNP | 17C | 2.04E-06 | G |  | CR44169 | intergenic variant |
| 2R:6881286 | 2R:2768791 | SNP | 17C | 2.04E-06 | G |  | Spn42Da | intergenic variant |
| 2R:6881286 | 2R:2768791 | SNP | 17C | 2.04E-06 | G |  | Spn42Db | intergenic variant |
| 2R:6881286 | 2R:2768791 | SNP | 17C | 2.04E-06 | G |  | Spn42Dc | intron variant |
| 2R:6881286 | 2R:2768791 | SNP | 17C | 2.04E-06 | G |  | Spn42Dc | intergenic variant |
| 2R:6881286 | 2R:2768791 | SNP | 17C | 2.04E-06 | G |  | Spn42Dd | intergenic variant |
| 2R:6881286 | 2R:2768791 | SNP | 17C | 2.04E-06 | G |  | Spn42De | intergenic variant |
| 2R:6890169 | 2R:2777674 | SNP | 17C | 7.13E-06 | G | T | CG30158 | intergenic variant |
| 2R:6890169 | 2R:2777674 | SNP | 17C | 7.13E-06 | G | T | Spn42De | intergenic variant |
| 2R:6941047 | 2R:2828552 | SNP | 17C | 1.06E-06 | C | A | CheB42b | intergenic variant |
| 2R:6941047 | 2R:2828552 | SNP | 17C | 1.06E-06 | C | A | CheB42c | intergenic variant |
| 2R:6941047 | 2R:2828552 | SNP | 17C | 1.06E-06 | C | A | mim | intron variant |
| 2R:6952053 | 2R:2839558 | SNP | 17C | 8.53E-06 | C | A | CheB42a | intergenic variant |
| 2R:6952053 | 2R:2839558 | SNP | 17C | 8.53E-06 | C | A | CheB42c | intron variant |
| 2R:6952053 | 2R:2839558 | SNP | 17C | 8.53E-06 | C | A | mim | intron variant |
| 2R:6952053 | 2R:2839558 | SNP | 17C | 8.53E-06 | C | A | ppk25 | synonymous variant |
| 2R:6952053 | 2R:2839558 | SNP | 17C | 8.53E-06 | C | A | tRNA:K2:42Eb | intergenic variant |
| 2R:6952053 | 2R:2839558 | SNP | 17C | 8.53E-06 | C | A | tRNA:K2:42Ec | intergenic variant |
| 2R:6952053 | 2R:2839558 | SNP | 17C | 8.53E-06 | C | A | tRNA:K2:42Ed | intergenic variant |
| 2R:6970206 | 2R:2857711 | SNP | 17C | 4.40E-06 | A | G | CG30157 | intergenic variant |
| 2R:6970206 | 2R:2857711 | SNP | 17C | 4.40E-06 | A | G | CheB42c | intron variant |
| 2R:6970206 | 2R:2857711 | SNP | 17C | 4.40E-06 | A | G | Cyp6u1 | intergenic variant |
| 2R:6970206 | 2R:2857711 | SNP | 17C | 4.40E-06 | A | G | mim | intron variant |
| 2R:7097150 | 2R:2984655 | SNP | 17C | 4.70E-07 | C | A | esn | intron variant |
| 2R:7097150 | 2R:2984655 | SNP | 17C | 4.70E-07 | C | A | esn | intergenic variant |
| 2R:7111859 | 2R:2999364 | SNP | 17C | 9.80E-06 | G | T | esn | intron variant |
| 2R:7671336 | 2R:3558841 | SNP | 17C | 8.70E-08 | G | A | CG12042 | intergenic variant |
| 2R:7671336 | 2R:3558841 | SNP | 17C | 8.70E-08 | G | A | CG12107 | intergenic variant |
| 2R:7671336 | 2R:3558841 | SNP | 17C | 8.70E-08 | G | A | CG12826 | intergenic variant |
| 2R:7671336 | 2R:3558841 | SNP | 17C | 8.70E-08 | G | A | CG2064 | intergenic variant |
| 2R:7671336 | 2R:3558841 | SNP | 17C | 8.70E-08 | G | A | mRpL52 | intergenic variant |
| 2R:7671336 | 2R:3558841 | SNP | 17C | 8.70E-08 | G | A | U2A | intergenic variant |
| 2R:7684354 | 2R:3571859 | SNP | 17C | 4.57E-06 | A | C | CG1399 | intron variant |
| 2R:7684354 | 2R:3571859 | SNP | 17C | 4.57E-06 | A | C | CG1399 | intergenic variant |
| 2R:7684443 | 2R:3571948 | SNP | 17C | 8.42E-06 | C | T | CG1399 | intron variant |
| 2R:7684443 | 2R:3571948 | SNP | 17C | 8.42E-06 | C | T | CG1399 | intergenic variant |
| 2R:7983239 | 2R:3870744 | SNP | 17C | 6.94E-06 | C | T | ACC | intron variant |
| 2R:7984225 | 2R:3871730 | SNP | 17C | 3.66E-06 | C | T | ACC | intron variant |
| 2R:7984225 | 2R:3871730 | SNP | 17C | 3.66E-06 | C | T | ACC | intergenic variant |
| 2R:7984225 | 2R:3871730 | SNP | 17C | 3.66E-06 | C | T | Nup44A | intergenic variant |
| 2R:7985641 | 2R:3873146 | SNP | 17C | 3.71E-06 | T | C | ACC | missense variant |
| 2R:7985641 | 2R:3873146 | SNP | 17C | 3.71E-06 | T | C | ACC | intron variant |
| 2R:7985641 | 2R:3873146 | SNP | 17C | 3.71E-06 | T | C | ACC | intergenic variant |
| 2R:7985641 | 2R:3873146 | SNP | 17C | 3.71E-06 | T | C | Nup44A | intergenic variant |
| 2R:8024790 | 2R:3912295 | SNP | 17C | 1.64E-07 | C | T | CR43724 | intergenic variant |
| 2R:8024790 | 2R:3912295 | SNP | 17C | 1.64E-07 | C | T | CR45267 | non coding transcript/exon variant |
| 2R:8032561 | 2R:3920066 | SNP | 17C | 4.42E-06 | G | T | CR43724 | intergenic variant |
| 2R:8032561 | 2R:3920066 | SNP | 17C | 4.42E-06 | G | T | Optix | intron variant |
| 2R:8106869 | 2R:3994374 | SNP | 17C | 6.73E-06 | T | C | CG11210 | intergenic variant |
| 2R:8106869 | 2R:3994374 | SNP | 17C | 6.73E-06 | T | C | Cul4 | intergenic variant |
| 2R:8106869 | 2R:3994374 | SNP | 17C | 6.73E-06 | T | C | udd | synonymous variant |
| 2R:8114430 | 2R:4001935 | SNP | 17C | 9.57E-06 | G | A | Asap | 3 prime UTR variant |
| 2R:8114430 | 2R:4001935 | SNP | 17C | 9.57E-06 | G | A | Asap | intergenic variant |
| 2R:8126880 | 2R:4014385 | INS | 17C | 1.69E-06 | T | TT | Asap | intergenic variant |
| 2R:8126880 | 2R:4014385 | INS | 17C | 1.69E-06 | T | TT | CG42516 | intergenic variant |
| 2R:8126880 | 2R:4014385 | INS | 17C | 1.69E-06 | T | TT | coil | 5 prime UTR variant |
| 2R:8126880 | 2R:4014385 | INS | 17C | 1.69E-06 | T | TT | coil | intron variant |
| 2R:8126880 | 2R:4014385 | INS | 17C | 1.69E-06 | T | TT | Nup50 | intergenic variant |
| 2R:8126880 | 2R:4014385 | INS | 17C | 1.69E-06 | T | TT | Pabp2 | intergenic variant |
| 2R:8126880 | 2R:4014385 | INS | 17C | 1.69E-06 | T | TT | Pbp49 | intergenic variant |
| 2R:8126880 | 2R:4014385 | INS | 17C | 1.69E-06 | T | TT | Socs44A | intergenic variant |
| 2R:8130938 | 2R:4018443 | SNP | 17C | 9.67E-06 | A | G | CG42516 | synonymous variant |
| 2R:8130938 | 2R:4018443 | SNP | 17C | 9.67E-06 | A | G | coil | intergenic variant |
| 2R:8130938 | 2R:4018443 | SNP | 17C | 9.67E-06 | A | G | Obp44a | intergenic variant |
| 2R:8130938 | 2R:4018443 | SNP | 17C | 9.67E-06 | A | G | Pabp2 | intergenic variant |
| 2R:8130938 | 2R:4018443 | SNP | 17C | 9.67E-06 | A | G | Pbp49 | 5 prime UTR variant |
| 2R:8130938 | 2R:4018443 | SNP | 17C | 9.67E-06 | A | G | Socs44A | intergenic variant |
| 2R:8160919 | 2R:4048424 | SNP | 17C | 2.27E-06 | G | A | CG30373 | intergenic variant |
| 2R:8160919 | 2R:4048424 | SNP | 17C | 2.27E-06 | G | A | CG8708 | synonymous variant |
| 2R:8160919 | 2R:4048424 | SNP | 17C | 2.27E-06 | G | A | CR30374 | intergenic variant |
| 2R:8160919 | 2R:4048424 | SNP | 17C | 2.27E-06 | G | A | CR44068 | intergenic variant |
| 2R:8160919 | 2R:4048424 | SNP | 17C | 2.27E-06 | G | A | kermit | intergenic variant |
| 2R:8160919 | 2R:4048424 | SNP | 17C | 2.27E-06 | G | A | Lpin | intergenic variant |
| 2R:8160919 | 2R:4048424 | SNP | 17C | 2.27E-06 | G | A | RagC-D | intergenic variant |
| 2R:8160919 | 2R:4048424 | SNP | 17C | 2.27E-06 | G | A | Rs1 | intergenic variant |
| 2R:8160919 | 2R:4048424 | SNP | 17C | 2.27E-06 | G | A | tRNA:CR30297 | intergenic variant |
| 2R:8265392 | 2R:4152897 | SNP | 17C | 1.13E-06 | A | G | CG12126 | intergenic variant |
| 2R:8265392 | 2R:4152897 | SNP | 17C | 1.13E-06 | A | G | CG14759 | intergenic variant |
| 2R:8265392 | 2R:4152897 | SNP | 17C | 1.13E-06 | A | G | CG2291 | intergenic variant |
| 2R:8265392 | 2R:4152897 | SNP | 17C | 1.13E-06 | A | G | CG30371 | intergenic variant |
| 2R:8265392 | 2R:4152897 | SNP | 17C | 1.13E-06 | A | G | CG30375 | intergenic variant |
| 2R:8265392 | 2R:4152897 | SNP | 17C | 1.13E-06 | A | G | CG30376 | intergenic variant |
| 2R:8280757 | 2R:4168262 | SNP | 17C | 4.00E-06 | A | G | CR44280 | intergenic variant |
| 2R:8280761 | 2R:4168266 | SNP | 17C | 3.76E-06 | T | G | CR44280 | intergenic variant |
| 2R:8295674 | 2R:4183179 | SNP | 17C | 4.24E-06 | C | T | mir-280 | intergenic variant |
| 2R:8464671 | 2R:4352176 | SNP | 17C | 5.98E-06 | A | C | Mal-A4 | intergenic variant |
| 2R:8464671 | 2R:4352176 | SNP | 17C | 5.98E-06 | A | C | Mal-A5 | synonymous variant |
| 2R:8464671 | 2R:4352176 | SNP | 17C | 5.98E-06 | A | C | Mal-A6 | intergenic variant |
| 2R:8464671 | 2R:4352176 | SNP | 17C | 5.98E-06 | A | C | Mal-A7 | intergenic variant |
| 2R:8492574 | 2R:4380079 | SNP | 17C | 1.80E-06 | A | T | mtt | intron variant |
| 2R:8494842 | 2R:4382347 | SNP | 17C | 5.35E-06 | A | T | mtt | intron variant |
| 2R:8494842 | 2R:4382347 | SNP | 17C | 5.35E-06 | A | T | mtt | intergenic variant |
| 2R:9491447 | 2R:5378952 | SNP | 17C | 6.76E-06 | G | A | Wnt2 | intergenic variant |
| 2R:9764696 | 2R:5652201 | SNP | 28C | 2.06E-06 | C | A | CG1688 | intergenic variant |
| 2R:9764696 | 2R:5652201 | SNP | 28C | 2.06E-06 | C | A | CR44205 | intergenic variant |
| 2R:9764696 | 2R:5652201 | SNP | 28C | 2.06E-06 | C | A | trpl | synonymous variant |
| 2R:9764735 | 2R:5652240 | SNP | 28C | 2.31E-06 | T | A | CG1688 | intergenic variant |
| 2R:9764735 | 2R:5652240 | SNP | 28C | 2.31E-06 | T | A | CR44205 | intergenic variant |
| 2R:9764735 | 2R:5652240 | SNP | 28C | 2.31E-06 | T | A | trpl | intron variant |
| 2R:9764747 | 2R:5652252 | SNP | 28C | 1.54E-06 | A | C | CG1688 | intergenic variant |
| 2R:9764747 | 2R:5652252 | SNP | 28C | 1.54E-06 | A | C | CR44205 | intergenic variant |
| 2R:9764747 | 2R:5652252 | SNP | 28C | 1.54E-06 | A | C | trpl | intron variant |
| 2R:9764749 | 2R:5652254 | SNP | 28C | 2.06E-06 | T | A | CG1688 | intergenic variant |
| 2R:9764749 | 2R:5652254 | SNP | 28C | 2.06E-06 | T | A | CR44205 | intergenic variant |
| 2R:9764749 | 2R:5652254 | SNP | 28C | 2.06E-06 | T | A | trpl | intron variant |
| 2R:9764757 | 2R:5652262 | SNP | 28C | 2.45E-06 | A | T | CG1688 | intergenic variant |
| 2R:9764757 | 2R:5652262 | SNP | 28C | 2.45E-06 | A | T | CR44205 | intergenic variant |
| 2R:9764757 | 2R:5652262 | SNP | 28C | 2.45E-06 | A | T | trpl | intron variant |
| 2R:9764761 | 2R:5652266 | SNP | 28C | 2.06E-06 | A | T | CG1688 | intergenic variant |
| 2R:9764761 | 2R:5652266 | SNP | 28C | 2.06E-06 | A | T | CR44205 | intergenic variant |
| 2R:9764761 | 2R:5652266 | SNP | 28C | 2.06E-06 | A | T | trpl | intron variant |
| 2R:9764766 | 2R:5652271 | SNP | 28C | 9.84E-06 | C | T | CG1688 | intergenic variant |
| 2R:9764766 | 2R:5652271 | SNP | 28C | 9.84E-06 | C | T | CR44205 | intergenic variant |
| 2R:9764766 | 2R:5652271 | SNP | 28C | 9.84E-06 | C | T | trpl | splice region variant/intron variant |
| 2R:9764769 | 2R:5652274 | SNP | 28C | 2.31E-06 | C | T | CG1688 | intergenic variant |
| 2R:9764769 | 2R:5652274 | SNP | 28C | 2.31E-06 | C | T | CR44205 | intergenic variant |
| 2R:9764769 | 2R:5652274 | SNP | 28C | 2.31E-06 | C | T | trpl | splice region variant/intron variant |
| 2R:9764776 | 2R:5652281 | SNP | 28C | 2.23E-06 | T | C | CG1688 | intergenic variant |
| 2R:9764776 | 2R:5652281 | SNP | 28C | 2.23E-06 | T | C | CR44205 | intergenic variant |
| 2R:9764776 | 2R:5652281 | SNP | 28C | 2.23E-06 | T | C | trpl | synonymous variant |
| 3L:10073500 | 3L:10066600 | SNP | 28C | 5.17E-06 | T | C | dpr6 | 3 prime UTR variant |
| 3L:10073500 | 3L:10066600 | SNP | 28C | 5.17E-06 | T | C | dpr6 | intergenic variant |
| 3L:10073500 | 3L:10066600 | SNP | 28C | 5.17E-06 | T | C | dpr6 | intron variant |
| 3L:10929014 | 3L:10922114 | SNP | 28C | 3.64E-06 | C | T | - | intergenic variant |
| 3L:12268596 | 3L:12261696 | SNP | 17C | 5.77E-06 | A | G | app | intergenic variant |
| 3L:12268596 | 3L:12261696 | SNP | 17C | 5.77E-06 | A | G | CG32100 | missense variant |
| 3L:12268596 | 3L:12261696 | SNP | 17C | 5.77E-06 | A | G | CG4300 | intergenic variant |
| 3L:12268596 | 3L:12261696 | SNP | 17C | 5.77E-06 | A | G | Pbgs | intergenic variant |
| 3L:12274185 | 3L:12267285 | INS | 17C | 7.44E-06 | T | TATA | CG10426 | intergenic variant |
| 3L:12274185 | 3L:12267285 | INS | 17C | 7.44E-06 | T | TATA | CG32100 | intergenic variant |
| 3L:12274185 | 3L:12267285 | INS | 17C | 7.44E-06 | T | TATA | CG4300 | intron variant |
| 3L:12274185 | 3L:12267285 | INS | 17C | 7.44E-06 | T | TATA | Pbgs | intergenic variant |
| 3L:16731698 | 3L:16724798 | SNP | 17C | 7.67E-06 | C | T | CG9701 | intergenic variant |
| 3L:16731698 | 3L:16724798 | SNP | 17C | 7.67E-06 | C | T | Dbp73D | intergenic variant |
| 3L:16731698 | 3L:16724798 | SNP | 17C | 7.67E-06 | C | T | PGRP-SB1 | intergenic variant |
| 3L:16731698 | 3L:16724798 | SNP | 17C | 7.67E-06 | C | T | PGRP-SB2 | intergenic variant |
| 3L:16744185 | 3L:16737285 | SNP | 17C | 9.49E-06 | T | C | CG9701 | intergenic variant |
| 3L:18397032 | 3L:18390132 | SNP | 17C | 5.37E-07 | T | A | rpr | intergenic variant |
| 3L:22975222 | 3L:22968322 | SNP | 17C | 4.50E-06 | T | A | - | intergenic variant |
| 3L:2354193 | 3L:2354193 | SNP | 28C | 9.24E-06 | G | T | - | intergenic variant |
| 3L:4117187 | 3L:4117187 | DEL | 28C | 3.25E-07 | CAC | C | Ack | intergenic variant |
| 3L:4117187 | 3L:4117187 | DEL | 28C | 3.25E-07 | CAC | C | Chd64 | intron variant |
| 3L:4117187 | 3L:4117187 | DEL | 28C | 3.25E-07 | CAC | C | Chd64 | intergenic variant |
| 3L:5045493 | 3L:5045493 | SNP | 17C | 9.42E-06 | A | T | Con | intron variant |
| 3L:6987430 | 3L:6980530 | SNP | 17C | 9.76E-06 | G | A | bin | intergenic variant |
| 3L:6987430 | 3L:6980530 | SNP | 17C | 9.76E-06 | G | A | CG32388 | intergenic variant |
| 3L:6987430 | 3L:6980530 | SNP | 17C | 9.76E-06 | G | A | CG43439 | intergenic variant |
| 3L:8627933 | 3L:8621033 | SNP | 28C | 8.08E-06 | T | A | Cdc6 | intergenic variant |
| 3L:8627933 | 3L:8621033 | SNP | 28C | 8.08E-06 | T | A | GAPsec | intergenic variant |
| 3L:8627933 | 3L:8621033 | SNP | 28C | 8.08E-06 | T | A | Zasp66 | intron variant |
| 3L:8627933 | 3L:8621033 | SNP | 28C | 8.08E-06 | T | A | Zasp66 | intergenic variant |
| 3L:9382789 | 3L:9375889 | SNP | 17C | 9.07E-06 | G | T | CG4080 | intergenic variant |
| 3L:9382789 | 3L:9375889 | SNP | 17C | 9.07E-06 | G | T | CR43481 | intergenic variant |
| 3L:9382789 | 3L:9375889 | SNP | 17C | 9.07E-06 | G | T | CR43482 | intergenic variant |
| 3L:9382789 | 3L:9375889 | SNP | 17C | 9.07E-06 | G | T | CR43483 | intergenic variant |
| 3L:9382789 | 3L:9375889 | SNP | 17C | 9.07E-06 | G | T | Hsp23 | intergenic variant |
| 3L:9382789 | 3L:9375889 | SNP | 17C | 9.07E-06 | G | T | Hsp27 | intergenic variant |
| 3L:9382789 | 3L:9375889 | SNP | 17C | 9.07E-06 | G | T | Hsp67Ba | intergenic variant |
| 3R:10678848 | 3R:6504570 | SNP | 28C | 6.50E-06 | G | T | CG14688 | missense variant |
| 3R:10678848 | 3R:6504570 | SNP | 28C | 6.50E-06 | G | T | CG6465 | intergenic variant |
| 3R:10678848 | 3R:6504570 | SNP | 28C | 6.50E-06 | G | T | CR44230 | intergenic variant |
| 3R:12875060 | 3R:8700782 | DEL | 28C | 6.11E-06 | GTATG | GG | - | intergenic variant |
| 3R:12935208 | 3R:8760930 | SNP | 28C | 2.22E-06 | G | A | beat-Vb | intergenic variant |
| 3R:12935208 | 3R:8760930 | SNP | 28C | 2.22E-06 | G | A | CR44236 | intergenic variant |
| 3R:22306386 | 3R:18132108 | SNP | 28C | 1.89E-06 | C | T | CG34377 | intergenic variant |
| 3R:22306386 | 3R:18132108 | SNP | 28C | 1.89E-06 | C | T | CG7084 | intergenic variant |
| 3R:26246961 | 3R:22072683 | SNP | 17C | 1.93E-06 | C | A | - | intergenic variant |
| 3R:28900875 | 3R:24726597 | SNP | 28C | 7.89E-06 | G | A | Doa | synonymous variant |
| 3R:28900875 | 3R:24726597 | SNP | 28C | 7.89E-06 | G | A | Doa | intergenic variant |
| 3R:29314477 | 3R:25140199 | SNP | 17C | 9.70E-06 | A | C | Cnx99A | intergenic variant |
| 3R:30669620 | 3R:26495342 | SNP | 17C | 9.66E-06 | T | A | CR45191 | intergenic variant |
| 3R:6696974 | 3R:2522696 | SNP | 17C | 5.37E-06 | A | G | Ccp84Ab | intergenic variant |
| 3R:6696974 | 3R:2522696 | SNP | 17C | 5.37E-06 | A | G | Ccp84Ac | intergenic variant |
| 3R:6696974 | 3R:2522696 | SNP | 17C | 5.37E-06 | A | G | Ccp84Ad | intergenic variant |
| 3R:6696974 | 3R:2522696 | SNP | 17C | 5.37E-06 | A | G | pncr002:3R | intergenic variant |
| 3R:6697688 | 3R:2523410 | SNP | 17C | 7.23E-06 | T | A | Ccp84Ab | intergenic variant |
| 3R:6697688 | 3R:2523410 | SNP | 17C | 7.23E-06 | T | A | Ccp84Ac | intergenic variant |
| 3R:6697688 | 3R:2523410 | SNP | 17C | 7.23E-06 | T | A | Ccp84Ad | intergenic variant |
| 3R:6697688 | 3R:2523410 | SNP | 17C | 7.23E-06 | T | A | pncr002:3R | intergenic variant |
| 3R:9034462 | 3R:4860184 | DEL | 28C | 9.80E-06 | TTT | T | hyx | intergenic variant |
| 3R:9034462 | 3R:4860184 | DEL | 28C | 9.80E-06 | TTT | T | neur | intron variant |
| 3R:9034462 | 3R:4860184 | DEL | 28C | 9.80E-06 | TTT | T | neur | intergenic variant |
| 3R:9042750 | 3R:4868472 | SNP | 28C | 6.97E-07 | C | A | hyx | intergenic variant |
| 3R:9042750 | 3R:4868472 | SNP | 28C | 6.97E-07 | C | A | neur | intergenic variant |
| 3R:9042750 | 3R:4868472 | SNP | 28C | 6.97E-07 | C | A | Nmdmc | synonymous variant |
| 3R:9042750 | 3R:4868472 | SNP | 28C | 6.97E-07 | C | A | Rel | intergenic variant |
| 3R:9049187 | 3R:4874909 | SNP | 28C | 1.92E-06 | G | A | Kdm2 | intergenic variant |
| 3R:9049187 | 3R:4874909 | SNP | 28C | 1.92E-06 | G | A | Mst85C | intron variant |
| 3R:9049187 | 3R:4874909 | SNP | 28C | 1.92E-06 | G | A | Mst85C | intergenic variant |
| 3R:9049187 | 3R:4874909 | SNP | 28C | 1.92E-06 | G | A | Nmdmc | 5 prime UTR variant |
| 3R:9049187 | 3R:4874909 | SNP | 28C | 1.92E-06 | G | A | Nmdmc | intron variant |
| 3R:9049187 | 3R:4874909 | SNP | 28C | 1.92E-06 | G | A | Nmdmc | intergenic variant |
| 3R:9049187 | 3R:4874909 | SNP | 28C | 1.92E-06 | G | A | Rel | intergenic variant |
| 3R:9052391 | 3R:4878113 | DEL | 28C | 6.81E-06 | GT | C | Kdm2 | intergenic variant |
| 3R:9052391 | 3R:4878113 | DEL | 28C | 6.81E-06 | GT | C | Mst85C | intergenic variant |
| 3R:9052391 | 3R:4878113 | DEL | 28C | 6.81E-06 | GT | C | Nmdmc | intergenic variant |
| 3R:9052391 | 3R:4878113 | DEL | 28C | 6.81E-06 | GT | C | Rel | intergenic variant |
| 3R:9056601 | 3R:4882323 | SNP | 28C | 2.33E-06 | G | A | Kdm2 | intron variant |
| 3R:9056601 | 3R:4882323 | SNP | 28C | 2.33E-06 | G | A | Mst85C | intergenic variant |
| 3R:9057142 | 3R:4882864 | SNP | 28C | 1.59E-06 | G | A | Kdm2 | intron variant |
| 3R:9057142 | 3R:4882864 | SNP | 28C | 1.59E-06 | G | A | Mst85C | intergenic variant |
| 3R:9057536 | 3R:4883258 | SNP | 28C | 1.13E-06 | C | A | Kdm2 | intron variant |
| 3R:9060562 | 3R:4886284 | SNP | 28C | 2.61E-06 | T | C | Ada | intergenic variant |
| 3R:9060562 | 3R:4886284 | SNP | 28C | 2.61E-06 | T | C | beag | intergenic variant |
| 3R:9060562 | 3R:4886284 | SNP | 28C | 2.61E-06 | T | C | Kdm2 | intron variant |
| 3R:9336375 | 3R:5162097 | SNP | 28C | 7.57E-06 | T | C | CG16749 | intergenic variant |
| 3R:9336375 | 3R:5162097 | SNP | 28C | 7.57E-06 | T | C | CG45050 | intergenic variant |
| 3R:9336375 | 3R:5162097 | SNP | 28C | 7.57E-06 | T | C | CG8121 | intron variant |
| 3R:9336375 | 3R:5162097 | SNP | 28C | 7.57E-06 | T | C | CG8866 | intergenic variant |
| 3R:9336375 | 3R:5162097 | SNP | 28C | 7.57E-06 | T | C | CR43301 | intergenic variant |
| X:13056960 | X:12950993 | SNP | 17C | 5.66E-07 | G | C | CG12715 | intergenic variant |
| X:13056960 | X:12950993 | SNP | 17C | 5.66E-07 | G | C | CR44840 | intergenic variant |
| X:13056960 | X:12950993 | SNP | 17C | 5.66E-07 | G | C | rad | intron variant |
| X:14863807 | X:14757840 | SNP | 17C | 8.19E-06 | T | C | CG9518 | 3 prime UTR variant |
| X:14863807 | X:14757840 | SNP | 17C | 8.19E-06 | T | C | Flo2 | intron variant |
| X:16820100 | X:16714133 | INS | 17C | 8.85E-06 | TTT | T | CG4829 | intron variant |
| X:16820100 | X:16714133 | INS | 17C | 8.85E-06 | TTT | T | CR44129 | intergenic variant |
| X:16820189 | X:16714222 | SNP | 17C | 6.36E-06 | T | C | CG4829 | intron variant |
| X:16820189 | X:16714222 | SNP | 17C | 6.36E-06 | T | C | CR44129 | intergenic variant |
| X:16820191 | X:16714224 | SNP | 17C | 6.97E-07 | T | C | CG4829 | intron variant |
| X:16820191 | X:16714224 | SNP | 17C | 6.97E-07 | T | C | CR44129 | intergenic variant |
| X:19714017 | X:19608050 | DEL | 17C | 1.53E-06 | T | TT | CG12531 | intergenic variant |
| X:19714017 | X:19608050 | DEL | 17C | 1.53E-06 | T | TT | CR44889 | intergenic variant |
| X:19714017 | X:19608050 | DEL | 17C | 1.53E-06 | T | TT | meso18E | intergenic variant |
| X:20003067 | X:19897100 | SNP | 28C | 4.57E-06 | T | G | CG17003 | intergenic variant |
| X:20003067 | X:19897100 | SNP | 28C | 4.57E-06 | T | G | Dop2R | intergenic variant |
| X:20003074 | X:19897107 | SNP | 28C | 9.32E-06 | A | T | CG17003 | intergenic variant |
| X:20003074 | X:19897107 | SNP | 28C | 9.32E-06 | A | T | Dop2R | intergenic variant |
| X:20003199 | X:19897232 | SNP | 28C | 1.99E-08 | T | C | CG17003 | intergenic variant |
| X:20003199 | X:19897232 | SNP | 28C | 1.99E-08 | T | C | Dop2R | intergenic variant |
| X:20535156 | X:20406183 | SNP | 28C | 8.97E-06 | T | G | RunxB | intergenic variant |
| X:20562471 | X:20433498 | SNP | 17C | 2.04E-06 | C | T | RunxB | intron variant |
| X:20565405 | X:20436432 | SNP | 17C | 4.96E-06 | C | G | RunxB | missense variant |
| X:20699151 | X:20570178 | SNP | 28C | 5.68E-06 | G | T | run | intergenic variant |
| X:2152985 | X:2047018 | SNP | 28C | 8.55E-06 | C | G | bcn92 | intergenic variant |
| X:2152985 | X:2047018 | SNP | 28C | 8.55E-06 | C | G | Pgd | intergenic variant |
| X:2152985 | X:2047018 | SNP | 28C | 8.55E-06 | C | G | wapl | intron variant |
| X:5388919 | X:5282952 | SNP | 28C | 2.83E-06 | A | T | SK | intron variant |
| X:5507085 | X:5401118 | SNP | 28C | 9.82E-06 | C | T | CG4151 | intergenic variant |
| X:5507085 | X:5401118 | SNP | 28C | 9.82E-06 | C | T | CR45519 | non coding transcript/exon variant |
